# Discovery and Biosynthesis of Celluxanthenes, Antibacterial Arylpolyene Alkaloids From Diverse Cellulose‐Degrading Anaerobic Bacteria

**DOI:** 10.1002/anie.202503697

**Published:** 2025-04-14

**Authors:** Keishi Ishida, Jana Krabbe, Philippe R. Meisinger, Gulimila Shabuer, Sebastian Schieferdecker, Michael Cyrulies, Cedric Tank, Emma Barnes, Christian Paetz, Christian Hertweck

**Affiliations:** ^1^ Department of Biomolecular Chemistry Leibniz Institute for Natural Product Research and Infection Biology (Leibniz‐HKI) Beutenbergstraße 11a 07745 Jena Germany; ^2^ BioPilot Plant, Leibniz Institute for Natural Product Research and Infection Biology Leibniz‐HKI 07745 Jena Germany; ^3^ Research Group Biosynthesis/NMR Max Planck Institute for Chemical Ecology 07745 Jena Germany; ^4^ Institute of Microbiology Faculty of Biological Sciences Friedrich Schiller University Jena 07743 Jena Germany

**Keywords:** Alkaloids, Biosynthesis, Natural Products, Polyenes, Polyketides

## Abstract

Cellulose degradation by anaerobic bacteria plays an eminent role in the global carbon cycle and is a critical step in biofuel production. The anaerobic thermophile *Clostridium thermocellum* (now: *Acetivibrio thermocellus*) is particularly efficient at breaking down biomass and produces a “yellow affinity substance” (YAS), a pigment that has been implicated in signaling and conferring higher affinity of the cellulosome to YAS‐loaded cellulose. However, the nature and biosynthetic origin of YAS have remained elusive. Here, we show by isolation and structure elucidation that YAS is a complex of unusual arylpolyene alkaloids (celluxanthenes). Stable isotope labeling experiments reveal all biosynthetic building blocks for celluxanthene assembly. Through a targeted gene deletion, we identify the celluxanthene (*cex*) biosynthesis gene cluster and propose a biosynthetic model in which an arylpolyene generated by an iterative type I polyketide synthase (PKS) undergoes a head‐to‐head fusion with a tryptophan‐derived ketoacid to form a tetronate. Genome mining and metabolic profiling revealed that diverse cellulolytic anaerobes harbor *cex* gene loci and produce celluxanthene congeners. Celluxanthenes show antibiotic activity against Gram‐positive bacteria including clinically relevant strains. This study solves the long‐standing enigma surrounding the nature of YAS and lays the groundwork for elucidating the precise biological roles of these intricate pigments.

## Graphical abstract

The structure and biosynthetic origin of the enigmatic “yellow affinity substance” (YAS) from the anaerobe thermophile *Clostridium thermocellum* (now: *Acetivibrio thermocellus*) were elucidated. YAS is a complex of unusual antibacterial arylpolyene alkaloids (celluxanthenes) that are produced by various cellulolytic anaerobes.

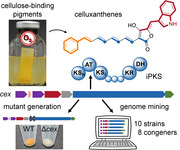

## Introduction

The most abundant organic material on Earth is cellulose, the primary component of plant cell walls that contributes to stability.^[^ ^1^ ^]^ This polysaccharide represents an enormous carbon sink that plays a major role in the global carbon cycle^[^ ^2^, ^3^ ^]^ and is increasingly appreciated as an alternative to fossil energy sources.^[^ ^4^, ^5^ ^]^ As a prerequisite, cellulose must first be hydrolyzed into its sugar building blocks.^[^ ^6^, ^7^, ^8^ ^]^ Due to its crystallinity, cellulose is extremely difficult to break down into its sugars, and only a limited number of microorganisms are capable of tapping into this energy source.^[^ ^9^, ^10^ ^]^

The best‐characterized cellulolytic bacteria belong to the phylum Firmicutes and the class Clostridia.^[^ ^9^, ^10^ ^]^ These meso‐ and thermophilic bacteria are ubiquitous in cellulose‐containing anaerobic habitats.^[^ ^11^ ^]^ To date, *Clostridium thermocellum* (*syn. Hungateiclostridium thermocellum* and *Acetivibrio thermocellus*)^[^ ^12^ ^]^ is the most efficient single biomass decomposer that has been characterized^[^ ^13^, ^14^, ^15^ ^]^ and is capable of fermenting cellulose to ethanol directly.^[^ ^16^ ^]^ One reason for this exceptional effectiveness at solubilizing cellulose and even lignocellulose is its multi‐component, self‐assembling cellulosome complex, which was discovered in the early 1980s and has since been the most studied of all cellulolytic anaerobes.^[^ ^17^, ^18^, ^19^, ^20^, ^21^ ^]^

In addition to this intricate enzyme complex, when growing on cellulose, *C. thermocellum* produces an unknown yellow‐orange, water‐insoluble pigment, which shows a strong affinity to crystalline cellulose.^[^ ^22^, ^23^, ^24^ ^]^ It has also been observed that this so‐called “yellow affinity substance” (YAS) precedes the production of cellulases,^[^ ^24^ ^]^ and that the cellulosome has a higher affinity to YAS‐loaded cellulose than to native cellulose.^[^ ^17^ ^]^ It has also been speculated that YAS functions as a signaling substance that informs cells of the presence of cellulose and induces the production of cellulase.^[^ ^24^ ^]^ However, to date the structure of YAS and the genetic basis for its biosynthesis have remained elusive, thus hampering in‐depth functional studies. Here, we reveal that YAS is a complex of pigment congeners, elucidate their structures, identify the corresponding biosynthetic gene cluster (BGC), provide first insight into their biosynthesis, and demonstrate by genome mining and metabolic profiling that this trait is widespread among important cellulolytic anaerobic bacteria.

## Results and Discussion

**Figure 1:**
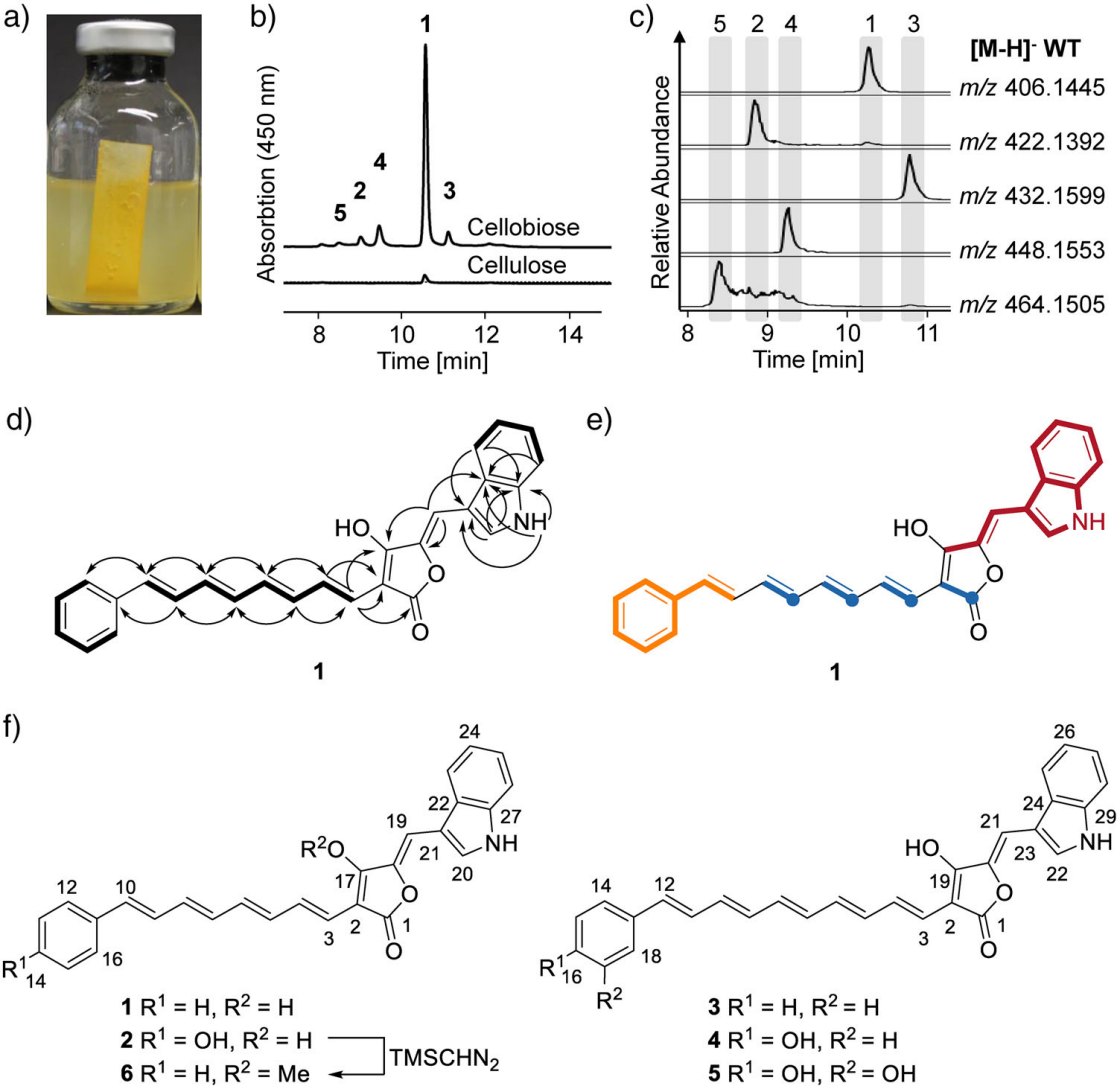
Pigment production of *C. thermocellum* and structure elucidation. a) Pigment attached to cellulose (filter paper). b) HPLC‐UV/Vis profile of ethyl acetate extracts of *C. thermocellum* after growth in cellulose or cellobiose based medium. c) EICs of compounds **1**–**5** from an ethyl acetate extract of *C. thermocellum* grown in cellobiose‐based medium. d) COSY correlations (bold lines), and HMBC correlations (arrows) of compound **1**. e) Summarized results of stable isotope labeling studies showing the biosynthetic building blocks for compound **1**: phenylacetate (orange), acetate via malonate (blue), and tryptophan (red). f) Structures of celluxanthenes A–E (**1**–**5**).

According to previous reports and our own experience,^[^ ^24^ ^]^ we have learned that the isolation of the YAS poses various challenges. First, due to its strong binding to cellulose surfaces (Figure 1a), the pigment is not readily extractable when grown in cellulose‐based media (Figure 1b). Second, despite their misleading color intensity the pigments are produced in only small amounts. Third, YAS proved to be highly unstable and prone to degradation. To address the first challenge, we cultured *C. thermocellum* in media containing alternative carbon sources. By supplementation with cellobiose, bacterial growth and pigment production could be restored. This allowed us to obtain yellow ethyl acetate extracts from cultured media (Figure 1b). Analysis of crude extracts by HPLC‐MS (Figure 1c) revealed the presence of five congeners (**1**–**5**) with similar MS/MS fragmentation patterns (Figures S1–S11).

To obtain sufficient amounts for structure elucidation, we upscaled the fermentation to 10L and optimized the extraction protocol using 2% XAD16 resin. After cultivation, the resin was removed by filtration and subsequently extracted with methanol, methanol/acetone, and acetone, with sonication, all in the absence of light and oxygen. The organic extract was subjected to reversed‐phase preparative HPLC.

The molecular formula (C_27_H_20_NO_3_) of the major congener **1** was deduced from HRMS analysis, which showed a pseudo‐molecular ion with a *m/z* 406.1445 [M─H]^‒^. The ^1^H‐NMR spectrum of **1** shows mainly aromatic and olefinic signals. One singlet proton (*δ*_H_ = 11.39, C20‐NH), which was obtrusively downfield shifted, appeared to be an amino proton of an indole moiety. The presence of an indole moiety was confirmed by a combination of ^1^H‐^1^H COSY correlations from a doublet proton H23 (*δ*_H_ = 7.06) to a doublet proton H26 (*δ*_H_ = 7.39) and eight key HMBC correlations between a singlet proton H20 (*δ*_H_ = 7.69) / C21 (*δ*_C_ = 109.4), C22 (*δ*_C_ = 126.6), and C27 (*δ*_C_ = 109.4), C20‐NH / C21, C22, and C27 (*δ*_C_ = 135.7), H23 / C27, H26 / C22. Three HMBC correlations from H19 to C17 (*δ*_C_ = 171.3), C18 (*δ*_C_ = 145.5), and C22 indicated that this moiety is adjacent to an olefinic carbon C19 (*δ*_C_ = 92.4). Two doublets of olefinic protons H12 / H16 (*δ*_H_ = 7.42) and a triplet of olefinic protons H13 / H15 (*δ*_H_ = 7.30), which are correlated to H14 (*δ*_H_ = 7.17) according to ^1^H‐^1^H COSY analysis, suggested the presence of a mono‐substituted benzene ring. One ^1^H‐^1^H COSY spin system from H3 to H10 and ^3^ *J*‐HMBC correlations pointed to a tetraene moiety and C10 connected to the benzene ring. Finally, by three ^3^ *J*‐HMBC correlations from H4 (*δ*_H_ = 6.89) to C2 (*δ*_C_ = 99.2) and H3 to C1 (*δ*_C_ = 177.0) and C17, the tetraene fragment was connected to the indole moiety via a tetronate moiety (Figures 1d and S12–S21, Table S1). The identity of the tetronate moiety was confirmed by methylation of the hydroxy group at C17 (**6**) using trimethylsilyldiazomethane and NMR analysis (Figures S22–S31 and Table S2).

Owing to their instability and low production of the other four congeners (**2**‒**5**), their structure elucidation proved to be challenging. However, judging from the molecular formula C_29_H_23_NO_3_ (*m/z* 432.1599 [M─H]^‒^) and extensive 2D NMR analysis (Figures S32–S43 and Table S3), we concluded that the polyene structure of **3** appeared to be one double bond longer than that of **1**. The molecular formulas of **2** (C_27_H_21_NO_4_) and **4** (C_29_H_23_NO_4_), deduced from HR‐MS, suggested that these compounds bear an additional oxygen functionality compared to **1** and **3**, respectively. Extensive 2D NMR analyses of **2** and **4**, including ^1^H‐^1^H COSY, HSQC, and HMBC, showed that **2** and **4** have *p*‐hydroxybenzene residues in lieu of the benzene rings of **1** and **3** (Figures S44–S67, Table S4–S5). The structure of the minor congener **5**, which is a homolog of **4** with an additional hydroxy group, was inferred mainly from HRMS and MS/MS fragmentation patterns (Figures S9–S11). With reference to their color and affinity to cellulose, we named compounds **1**–**5** celluxanthenes A–E (Figure 1f).

The molecular scaffold of the celluxanthene is intriguing. Known bacterial aryl polyene polyketides consist of a linear alkene chain that is capped with a phenyl ring and a carbonyl head group in form of a carboxylic acid ester or amide group as, for example, polyenomycin A,^[^ ^25^ ^]^ xanthomonadins,^[^ ^26^ ^]^ flexirubin,^[^ ^27^ ^]^ and bromoalterochromide A.^[^ ^28^ ^]^ Celluxanthenes are the first examples of polyene pigments that contain an indole heterocycle as part of the chromophore and represent a novel family of bacterial aryl polyene alkaloids. In addition, celluxanthenes bear a tetronate moiety, which occurs in various natural products,^[^ ^29^ ^]^ either as a free head group of a linear alkyl chain (e.g., thiazone A)^[^ ^30^ ^]^ or as part of a polycyclic scaffold (e.g., abyssomycin C).^[^ ^31^ ^]^ Celluxanthenes are the first examples of *γ*‐ylidene tetronates that are connected to an indole ring via a methine bridge.

To further confirm the proposed structures and to gain a first insight into the biosynthetic building blocks, we performed stable‐isotope labeling studies with [2,3,5,6‐D_4_]‐*p*‐hydroxybenzoic acid, [D_7_]‐phenylacetic acid, [1,3–^13^C_2_]‐malonic acid, [1,2–^13^C_2_]‐acetic acid, and [^13^C_11_]‐l‐tryptophan. HPLC‐MS and MS/MS analysis of the culture extracts indicated that phenylacetic acid (but not hydroxybenzoate), acetate (via malonate), and l‐tryptophan were incorporated into **1** (Figures S68–S71). Supplemented malonic acid is likely not taken up by the cells. Thus, we concluded that **1** is assembled from phenylacetate, four malonate units, and one l‐tryptophan‐derived building block (Figure 1e). Since [2,3,5,6‐D_4_]‐*p*‐hydroxybenzoic acid was not incorporated into the hydroxylated compounds **2** and **4**, we reasoned that their biosynthesis involves *p*‐hydroxyphenyl acetic acid.

**Figure 2:**
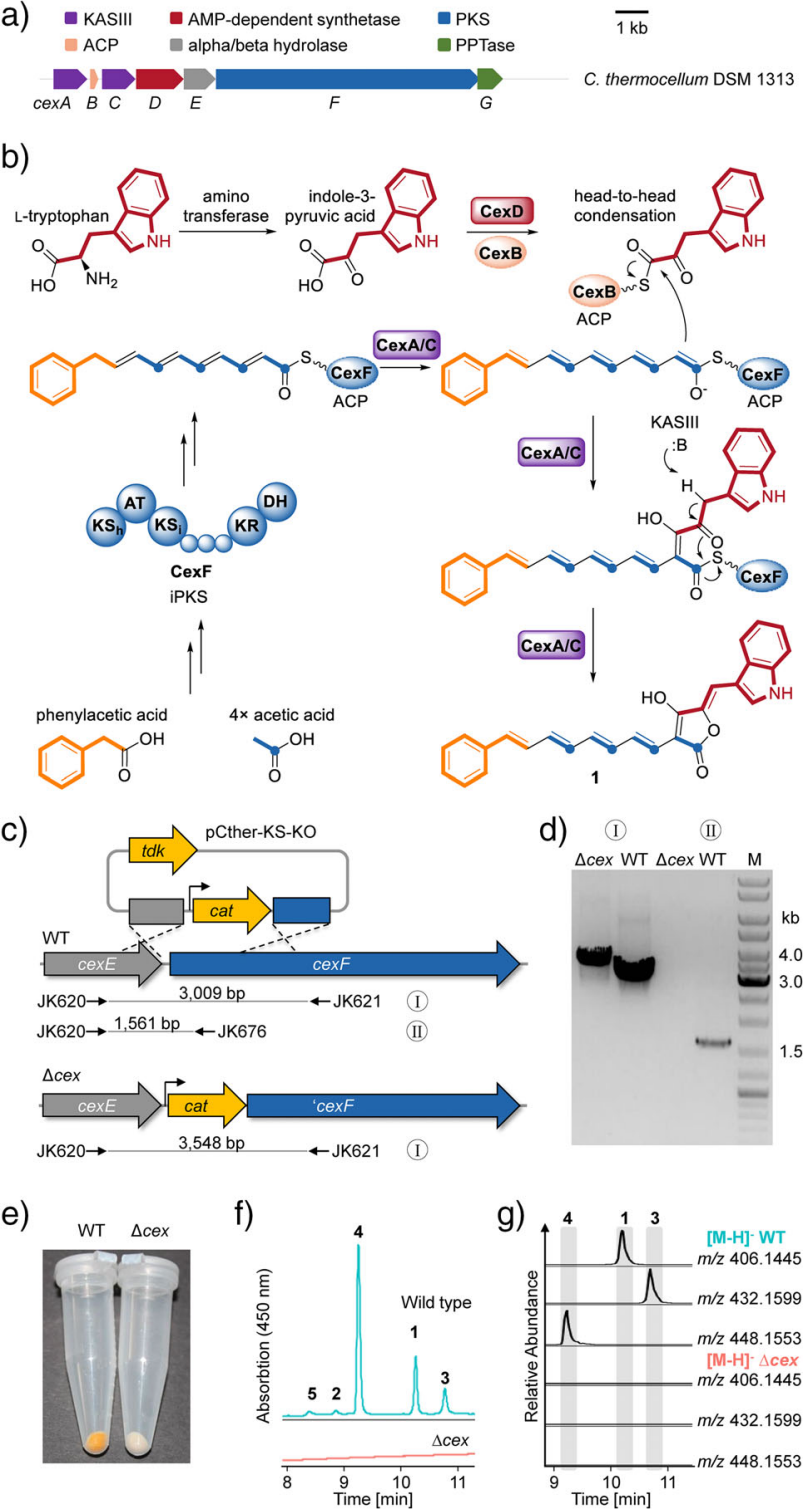
Identification of the celluxanthene BGC, and biosynthetic model. a) Genetic architecture of the *cex* gene cluster from *C. thermocellum* DSM 1313. b) Scheme showing a model of celluxanthene biosynthesis based on stable isotope labeling and deduced gene functions. KAS, β‐ketoacyl ACP synthase, KS, ketosynthase, AT, acyl transferase, KR, ketoreductase, DH, dehydratase, ACP, acyl carrier protein. c) Mutant construction by double crossover. d) PCR‐based verification of the Δ*cex* mutant. The uncropped gel image is shown in Figure S72B. e) Pigmentation of *C. thermocellum* wild type and mutant. f) HPLC UV profiles. g) EICs of extracts.

The arylpolyene structures of **1**–**5** suggest that their biosynthesis involves a type I polyketide synthase (PKS). Mining the genome of *C. thermocellum* with AntiSMASH,^[^ ^32^ ^]^ only one suitable candidate for a celluxanthene biosynthesis gene cluster (*cex*) was identified (Table S6). The *cex* gene locus contains genes putatively coding for a PKS with an unusual architecture (CexF), a phosphopantetheinyl transferase (PPTase, CexG), an AMP‐dependent synthetase (CexD), two β‐ketoacyl‐ACP synthases (KASIII, CexAC), an acyl carrier protein (ACP, CexB), and an alpha/beta hydrolase (CexE) (Figure 2a). A homology search of CexF indicated that it belongs to the small family of bacterial iterative PKSs (iPKS), which are involved in the biosynthesis of important specialized metabolites such as polyunsaturated fatty acids,^[^ ^33^, ^34^ ^]^ enediynes,^[^ ^35^, ^36^, ^37^, ^38^ ^]^ PoTeMs,^[^ ^39^ ^]^ and arylpolyenes.^[^ ^26^, ^40^, ^41^, ^42^ ^]^

These deduced gene functions would fit to a plausible biosynthetic model, which is in agreement with the isotope labeling experiments. Accordingly, an arylpolyene chain would be assembled by an iterative PKS (CexF) and merged with a trp‐derived building block, most likely via an α‐keto intermediate formed by transamination. The presence of ligase CexD and *cis*‐ and *trans*‐acting ACPs suggests that this process is thiotemplated. These two ACP‐bound intermediates would be fused by a head‐to‐head condensation catalyzed by both or one of the two KASIIIs (CexA and CexC) in analogy to α‐pyrone formation.^[^ ^43^, ^44^ ^]^ However, due to the presence of an α‐oxo species, likely in the form of indole‐3‐pyruvyl‐*S*‐ACP, the arylpolyene‐indolyl hybrid would be cyclized into a tetronate upon a global double bond shift and release from CexF. Several tetronate formations using KASIII have been reported that use glyceryl‐*S*‐ACP and β‐ketoacyl‐*S*‐ACP as reaction partners.^[^ ^44^, ^45^ ^]^ These substrates clearly differ from indole‐3‐pyruvyl‐*S*‐ACP and alkenylacyl‐*S*‐ACP in celluxanthene biosynthesis. In our proposed pathway, the O‐nucleophile that is responsible for chain release consists of an enolate instead of a simple hydroxy group as for glyceryl‐*S*‐ACP (Figure 2b).

To correlate the *cex* locus with the production of the celluxanthene derivatives, we aimed to generate a mutant deficient in the essential PKS gene. However, targeted gene deletions or disruptions in clostridia, especially thermophilic clostridia are notoriously difficult.^[^ ^46^ ^]^ After elaborating different methods for the genetic manipulation of *C. thermocellum*, we successfully generated a double‐crossover mutant by homologous recombination (Figure 2c and d, Figure S72).^[^ ^47^ ^]^ This mutant visibly lacks the yellow pigmentation of the wild type (Figure 2e), which was confirmed by HPLC analysis (Figure 2f and 2g).

According to a previous report,^[^ ^48^ ^]^ *Ruminococcus flavefaciens* DSM 25089 was assumed to produce the same or a similar pigment as *C. thermocellum*. Knowledge of the arylpolyene structures and the genetic basis for their biosynthesis allowed us to revisit this proposal. Notably, the genome of *R. flavefaciens* DSM 25089 does not have any *cex*‐like gene cluster, and none of the celluxanthene congeners could be detected in its cultures.

**Figure 3:**
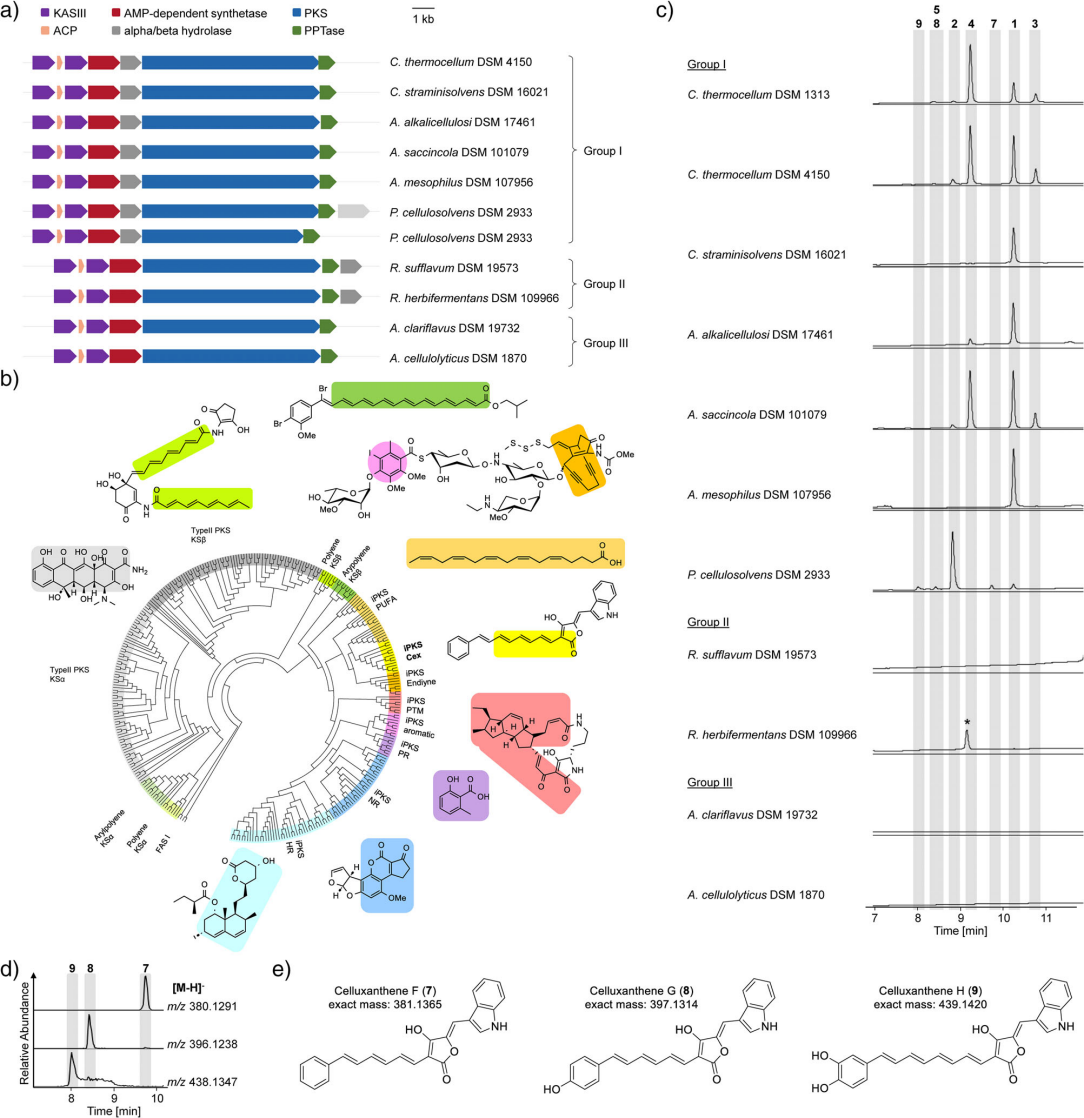
Unearthing the potential of cellulolytic anaerobes to produce celluxanthenes. a) Genome mining and comparison of *cex*‐like gene clusters from other cellulose‐degrading anaerobic bacteria. b) Phylogenetic analysis of iPKSs from *cex* gene clusters and other types of PKSs based on KS sequences. c) Detection of celluxanthene congeners in diverse cellulolytic anaerobes by HPLC‐MS. d) EICs of **7**–**9**. e) Proposed structures of celluxanthenes F−H (**7**–**9**). Asterisk indicates a compound that is unrelated to celluxanthenes (verified by MS/MS).

The discovery of the highly specialized arylpolyene biosynthetic pathway prompted us to search for *cex*‐like BGCs in other anaerobes. By BLAST analysis using the CexF sequence as a handle, we detected orthologous iterative PKS genes in numerous genomes of cellulolytic anaerobic bacteria (Figure 3a). These include another *C. thermocellum* strain (DSM 4150), *Clostridium straminisolvens* (DSM 16021), *Acetivibrio alkalicellulosi* (DSM 17461), *Acetivibrio saccincola* (DSM 101079), *Acetivibrio mesophilus* (DSM 107956), *Pseudobacteroides cellulosolvens* (DSM 2933), *Ruminiclostridium herbifermentans* (DSM 109966), *Ruminiclostridium sufflavum* (DSM 19573,) *Acetivibrio clariflavus* (DSM 19732), and *Acetivibrio cellulolyticus* (DSM 1870). Notably, in the cladogram, the KS domains of their iPKSs fall into the same clade as that of CexF (Figure 3b and Table S7), and the genetic context of the *cexF* orthologues is highly similar. Yet, there are slight differences in the architectures of the *cex*‐like BGCs. BGCs of group I are highly conserved; in group II, the position of the α/β‐hydrolase gene has changed; and in group III, the α/β‐hydrolase gene is lost.

To investigate the potential of these bacteria to produce celluxanthene or related arylpolyenes, we cultured all strains independently and monitored for PDA signals of polyenes (λ 420–450 nm) and/or for the known masses of **1**–**5** (Figures S73–S79). In this way, we detected similar pigment production in most strains of group I, in which the *cex* gene cluster is practically identical with the one of *C. thermocellum* (DSM 1313) (Figure 3c). Interestingly, only one strain of group I (*Pb. cellulosolvens*) shows a distinct profile. This strain produces **2** as the main compound, and instead of **3**–**5** we detected three other compounds (**7**–**9**). Their molecular composition, deduced from HRMS (*m/z* 380.1291 [M‐H]^−^, 396.1238 [M‐H]^−^, and 438.1347 [M‐H]^−^) (Figures 3d and S80–S85) and MS/MS fragmentation (Figure S86) suggested that celluxanthenes F (**7**) and G (**8**) have a shorter polyene linker (triene), and that celluxanthene H (**9**) is a congener with a catechol moiety (Figure 3e). Notably, strains of groups II and III, where the α/β‐hydrolase is either repositioned or absent, do not produce any of the celluxanthene derivates under the same conditions (Figure 3c). Thus, it appears that the α/β‐hydrolase plays a regulatory role. Taken together, our findings show that celluxanthenes are widely distributed in cellulolytic anaerobic bacteria, which suggests an important role of this pigment for these biomass decomposers.

Unexpectedly, we did not observe any pronounced differences in growth behavior and cellulose consumption between the wild type and the Δ*cex* mutant when grown on cellulose (Figures S87–S88). Under the conditions tested, these results do not support the idea of a signaling function of the pigments as previously hypothesized and may suggest other biological roles.^[^ ^24^ ^]^ Since various tetronates have been shown to exhibit moderate antibacterial activities,^[^ ^49^, ^50^ ^]^ we performed antimicrobial agar diffusion assays using isolated **1** at a concentration of 1 mg mL^−1^ (2.45 mM). Whereas selected fungi and Gram‐negative bacteria were not sensitive towards **1**, we detected moderate activities of **1** against Gram‐positive bacteria including clinically relevant genera (Table S11). Evaluation of minimal inhibition concentrations (MICs) showed approximate MIC values of 100 µg mL^−1^ (245 µM) for methicillin‐resistant *Staphylococcus aureus* (MRSA) and 25–50 µg mL^−1^ (61.25–122.5 µM) for *Mycobacterium vaccae*. The antibacterial activity of the cellulose‐binding pigments may play a role as a means to ward off competitors and to claim an important carbon source, similar to clostrubin produced by pectinolytic anaerobes.^[^ ^51^, ^52^ ^]^ Future functional studies will shed more light on the ecological function of celluxanthenes.

## Conclusion

This study solves the long‐standing riddle of the identity of the enigmatic “yellow affinity substance” of *C. thermocellum*, which is not only ecologically important but also has a high translational value in biotechnology. Our successful isolation and structure elucidation reveal that YAS is not a single compound but a complex of congeners, named celluxanthenes, which share an unprecedented arylpolyene alkaloid architecture.^[^ ^53^ ^]^ The unusual celluxanthene complex is an important addition to the known specialized metabolites from anaerobes, which remain an underexplored source of bioactive compounds,^[^ ^54^ ^]^ many of which have unique structures.^[^ ^51^, ^52^, ^55^, ^56^, ^57^, ^58^, ^59^ ^]^ Thenovel celluxanthene scaffold may inspire the design of compounds with high cellulose affinity. Our biosynthetic studies point to a previously undescribed convergent pathway, an unusual head‐to‐head fusion of an arylpolyene with a tryptophan‐derived ketoacid to form a tetronate‐containing scaffold, and uncovers a new family of iterative polyketide synthases, the first of their kind in anaerobes. The identification and assignment of the *cex* gene cluster by targeted gene deletion provides a valuable mutant for functional studies of this chemotype and sets the basis for bioengineering approaches. Furthermore, we have demonstrated that thisknowledge can be used for genome mining and showed that various cellulolytic anaerobic bacteria of different genera employ celluxanthenes. Our finding that celluxanthene exhibits antibiotic activity against Gram‐positive bacteria suggests a novel ecological function of cellulose‐binding pigments to secure the niche and claim an important food source. This study not only provides fundamental insights into the chemical ecology of cellulolytic bacteria but also establishes a foundation for future research into the functional roles of celluxanthenes in microbial metabolism, signaling, and plant biomass utilization.

## Supporting Information

The authors have cited additional references in the supporting information.^[^ ^60^, ^61^, ^62^, ^63^, ^64^, ^65^, ^66^, ^67^, ^68^, ^69^ ^]^

## Conflict of Interests

The authors declare no conflict of interest.

## Supporting information



Supporting Information

## Data Availability

The data that support the findings of this study are available in the supplementary material of this article.
