# Supplementary material for: Discovery and Biosynthesis of Celluxanthenes, Antibacterial Arylpolyene Alkaloids From Diverse Cellulose‐Degrading Anaerobic Bacteria
Source: Angew Chem Int Ed Engl. 2025 Apr 14;64(24):e202503697. doi: 10.1002/anie.202503697 (PMC12144888; doi:10.1002/anie.202503697)
Supplement: Supplementary file 1 — Supporting Information [file ANIE-64-e202503697-s001.pdf]

Table of Contents

General methods
Clostridium thermocellum culture conditions
Fermentation for isolation of celluxanthenes
Isolation of celluxanthenes (**1–4**)
Methylation of celluxanthene A (**1**).
General analytical procedures
Plasmid construction
Preparation of *C. thermocellum* mutant strain
Cultivation of additional anaerobic strains for potential celluxanthene production in 1L-fermenter
Phylogenetic analysis

Supplemental Tables

Table S1. ^{1}H (600 MHz) and ^{13}C (150 MHz) NMR data of celluxanthene A (**1**) in DMSO-d_6.
Table S2. ^{1}H (750 MHz) and ^{13}C (176 MHz) NMR data of 17-methoxycelluxanthene A (**6**) in CD_2Cl_2.
Table S3. ^{1}H (600 MHz) and ^{13}C (150 MHz) NMR data of celluxanthene B (**2**) in DMSO-d_6.
Table S4. ^{1}H (600 MHz) and ^{13}C (150 MHz) NMR data of celluxanthene C (**3**) in DMSO-d_6.
Table S5. ^{1}H (600 MHz) and ^{13}C (150 MHz) NMR data of celluxanthene D (**4**) in DMSO-d_6.
Table S6. Celluxanthene biosynthesis genes and genes in flanking regions.
Table S7. Keto synthase (KS) proteins used for Maximum Likelihood phylogenetic tree.
Table S8. Strains used in this study
Table S9. Plasmids used in this study.
Table S10. Primers used in this study.
Table S11. Inhibitory effects of celluxanthene A against several bacterial and fungal strains.

Supplemental Figures

Figure S1. Negative HR-MS spectrum of celluxanthene A (**1**).
Figure S2. Positive HR-MS spectrum of celluxanthene A (**1**).
Figure S3. Negative HR-MS spectrum of celluxanthene B (**2**).
Figure S4. Positive HR-MS spectrum of celluxanthene B (**2**).
Figure S5. Negative HR-MS spectrum of celluxanthene C (**3**).
Figure S6. Positive HR-MS spectrum of celluxanthene C (**3**).
Figure S7. Negative HR-MS spectrum of celluxanthene D (**4**).
Figure S8. Positive HR-MS spectrum of celluxanthene D (**4**).
Figure S9. Negative HR-MS spectrum of celluxanthene E (**5**).
Figure S10. Positive HR-MS spectrum of celluxanthene E (**5**).
Figure S11. Positive HR-MS/MS profile of celluxanthene A–E (**1–5**).
Figure S12. ^{1}H NMR spectrum of celluxanthene A (**1**) in DMSO-d_6 at 300K.
Figure S13. Extended ^{1}H NMR spectrum of celluxanthene A (**1**) in DMSO-d_6 at 300K.
Figure S14. ^{13}C NMR spectrum of celluxanthene A (**1**) in DMSO-d_6 at 300K.
Figure S15. Extended ^{13}C NMR spectrum of celluxanthene A (**1**) in DMSO-d_6 at 300K.
Figure S16. DEPTQ spectrum of celluxanthene A (**1**) in DMSO-d_6 at 300K.
Figure S17. ^{1}H-^{1}H COSY spectrum of celluxanthene A (**1**) in DMSO-d_6 at 300K.

Figure S18. Extended ^{1}H-^{1}H COSY spectrum of celluxanthene A (**1**) in DMSO-d_6 at 300K.
Figure S19. HSQC spectrum of celluxanthene A (**1**) in DMSO-d_6 at 300K.
Figure S20. HMBC spectrum of celluxanthene A (**1**) in DMSO-d_6 at 300K.
Figure S21. Extended HMBC spectrum of celluxanthene A (**1**) in DMSO-d_6 at 300K.
Figure S22. Negative HR-MS spectrum of 17-methoxycelluxanthene A (**6**).
Figure S23. Positive HR-MS spectrum of 17-methoxycelluxanthene A (**6**).
Figure S24. ^{1}H NMR spectrum of 17-methoxycelluxanthene A (**6**) in CD_2Cl_2 at 300K.
Figure S25. Extended ^{1}H NMR spectrum of 17-methoxycelluxanthene A (**6**) in CD_2Cl_2 at 300K.
Figure S26. DEPTQ spectrum of 17-methoxycelluxanthene A (**6**) in CD_2Cl_2 at 300K.
Figure S27. DQF-COSY spectrum of 17-methoxycelluxanthene A (**6**) in CD_2Cl_2 at 300K.
Figure S28. HSQC spectrum of 17-methoxycelluxanthene A (**6**) in CD_2Cl_2 at 300K.
Figure S29. HMBC spectrum of 17-methoxycelluxanthene A (**6**) in CD_2Cl_2 at 300K.
Figure S30. ROESY spectrum of 17-methoxycelluxanthene A (**6**) in CD_2Cl_2 at 300K.
Figure S31. Selected ^{1}H-^{1}H COSY (bold lines) and HMBC (arrows) correlations of **6**.
Figure S32. ^{1}H NMR spectrum of celluxanthene C (**3**) in DMSO-d_6 at 300K.
Figure S33. Extended ^{1}H NMR spectrum of celluxanthene C (**3**) in DMSO-d_6 at 300K.
Figure S34. ^{13}C NMR spectrum of celluxanthene C (**3**) in DMSO-d_6 at 300K.
Figure S35. Extended ^{13}C NMR spectrum of celluxanthene C (**3**) in DMSO-d_6 at 300K.
Figure S36. ^{1}H-^{1}H COSY spectrum of celluxanthene C (**3**) in DMSO-d_6 at 300K.
Figure S37. Extended ^{1}H-^{1}H COSY spectrum of celluxanthene C (**3**) in DMSO-d_6 at 300K.
Figure S38. HSQC spectrum of celluxanthene C (**3**) in DMSO-d_6 at 300K.
Figure S39. HMBC spectrum of celluxanthene C (**3**) in DMSO-d_6 at 300K.
Figure S40. Extended HMBC spectrum of celluxanthene C (**3**) in DMSO-d_6 at 300K.
Figure S41. ROESY spectrum of celluxanthene C (**3**) in DMSO-d_6 at 300K.
Figure S42. Extended ROESY spectrum of celluxanthene C (**3**) in DMSO-d_6 at 300K.
Figure S43. Selected ^{1}H-^{1}H COSY (bold lines) and HMBC (arrows) correlations of **3**.
Figure S44. ^{1}H NMR spectrum of celluxanthene B (**2**) in DMSO-d_6 at 300K.
Figure S45. Extended ^{1}H NMR spectrum of celluxanthene B (**2**) in DMSO-d_6 at 300K.
Figure S46. ^{13}C NMR spectrum of celluxanthene B (**2**) in DMSO-d_6 at 300K.
Figure S47. Extended ^{13}C NMR spectrum of celluxanthene B (**2**) in DMSO-d_6 at 300K.
Figure S48. ^{1}H-^{1}H COSY spectrum of celluxanthene B (**2**) in DMSO-d_6 at 300K.
Figure S49. Extended ^{1}H-^{1}H COSY spectrum of celluxanthene B (**2**) in DMSO-d_6 at 300K.
Figure S50. HSQC spectrum of celluxanthene B (**2**) in DMSO-d_6 at 300K.
Figure S51. HMBC spectrum of celluxanthene B (**2**) in DMSO-d_6 at 300K.
Figure S52. Extended HMBC spectrum of celluxanthene B (**2**) in DMSO-d_6 at 300K.
Figure S53. ROESY spectrum of celluxanthene B (**2**) in DMSO-d_6 at 300K.
Figure S54. Extended ROESY spectrum of celluxanthene B (**2**) in DMSO-d_6 at 300K.
Figure S55. Selected ^{1}H-^{1}H COSY (bold lines) and HMBC (arrows) correlations of **2**.
Figure S56. ^{1}H NMR spectrum of celluxanthene D (**4**) in DMSO-d_6 at 300K.
Figure S57. Extended ^{1}H NMR spectrum of celluxanthene D (**4**) in DMSO-d_6 at 300K.
Figure S58. ^{13}C NMR spectrum of celluxanthene D (**4**) in DMSO-d_6 at 300K.
Figure S59. Extended ^{13}C NMR spectrum of celluxanthene D (**4**) in DMSO-d_6 at 300K.
Figure S60. ^{1}H-^{1}H COSY spectrum of celluxanthene D (**4**) in DMSO-d_6 at 300K.
Figure S61. Extended ^{1}H-^{1}H COSY spectrum of celluxanthene D (**4**) in DMSO-d_6 at 300K.
Figure S62. HSQC spectrum of celluxanthene D (**4**) in DMSO-d_6 at 300K.
Figure S63. HMBC spectrum of celluxanthene D (**4**) in DMSO-d_6 at 300K.
Figure S64. Extended HMBC spectrum of celluxanthene D (**4**) in DMSO-d_6 at 300K.
Figure S65. ROESY spectrum of celluxanthene D (**4**) in DMSO-d_6 at 300K.
Figure S66. Extended ROESY spectrum of celluxanthene D (**4**) in DMSO-d_6 at 300K.

Figure S67. Selected ^{1}H-^{1}H COSY (bold lines) and HMBC (arrows) correlations of **4**.
Figure S68. Positive HR-MS profile of celluxanthene A (**1**) of EtOAc extracts obtained from isotope labeled substrate feeding experiments.
Figure S69. Positive HR-MS profile of celluxanthene B (**2**) of EtOAc extracts obtained from [2,3,5,6-D_4]-*p*-hydroxybenzoic acid feeding experiments.
Figure S70. Positive HR-MS/MS profile of celluxanthene A (**1**) of EtOAc extracts obtained from isotope labeled substrates feeding experiments.
Figure S71. Negative HR-MS profile of celluxanthene A (**1**) of EtOAc extracts obtained from [$^{13}C_{11}$]-L-tryptophan feeding experiments.
Figure S72. Generation of *C. thermocellum* Δ*cex* mutant.
Figure S73. PDA (420–450 nm) and (–)EIC profile of celluxanthene A–E (**1**–**5**) of *Clostridium thermocellum* DSM 1313 extract.
Figure S74. PDA (420–450 nm) and (–)EIC profile of celluxanthene A–E (**1**–**5**) of *Clostridium thermocellum* DSM 4150 extract.
Figure S75. PDA (420–450 nm) and (–)EIC profile of celluxanthene A–E (**1**–**5**) of *Clostridium straminisolvens* DSM 16021 extract.
Figure S76. PDA (420–450 nm) and (–)EIC profile of celluxanthene A–E (**1**–**5**) of *Acetivibrio alkalicellulosi* DSM 17461 extract.
Figure S77. PDA (420–450 nm) and (–)EIC profile of celluxanthene A–E (**1**–**5**) of *Acetivibrio saccincola* DSM 101079 extract.
Figure S78. PDA (420–450 nm) and (–)EIC profile of celluxanthene A–E (**1**–**5**) of *Acetivibrio mesophilus* DSM 107956 extract.
Figure S79. PDA (420–450 nm) and (–)EIC profile of celluxanthene A–B (**1**–**2**) and F–H (**7**–**9**) of *Pseudobacteroides cellulosolvens* DSM 2933 extract.
Figure S80. Negative HR-MS spectrum of celluxanthene F (**7**).
Figure S81. Positive HR-MS spectrum of celluxanthene F (**7**).
Figure S82. Negative HR-MS spectrum of celluxanthene G (**8**).
Figure S83. Positive HR-MS spectrum of celluxanthene G (**8**).
Figure S84. Negative HR-MS spectrum of celluxanthene H (**9**).
Figure S85. Positive HR-MS spectrum of celluxanthene H (**9**).
Figure S86. Positive HR-MS/MS profile of celluxanthene A, F–H (**1, 7**–**9**).
Figure S87. Growth curve of *C. thermocellum* DSM1313 wild type and Δ*cex* mutant strain in a 1L-fermenter on cellulose.
Figure S88. Cellulose consumption of *C. thermocellum* DSM1313 wild type and Δ*cex* mutant strain during growth in a 1L-fermenter on cellulose.

Supplemental References

General methods

Unless otherwise stated, all enzymes were purchased from New England Biolabs. Sequencing was done by Azenta/Genewiz. Oligonucleotide primer synthesis was performed by Eurofins Genomics. Media components were purchased from Sigma, Roth and Difco. If not stated otherwise, composition of media can be found at the DSMZ web page. All strains, plasmids, and oligonucleotide primers used in this study are listed in Table S8, Table S9, and Table S10.

Clostridium thermocellum culture conditions

Clostridium thermocellum DSM 1313 and DSM 4150 were obtained from DSMZ GmbH (Braunschweig). Strains were grown anaerobically in modified CTFUD-rich,[60] MJ defined medium,[61] or M122 in sealed Schott or glass serum bottles at 55 °C without shaking. As C-source either cellobiose or cellulose was used. Mutant strains were kept with 6 µg mL^{-1} thiamphenicol.

Fermentation for isolation of celluxanthenes

Production strains were grown in batch culture in bioreactors at volumes ranging from 500 mL (BIOSTAT Q plus, Sartorius Stedim) to 5 L (BIOSTAT B, Sartorius Stedim). Anaerobic conditions were maintained by continuous sparging the bioreactors with nitrogen for at least 3 h prior to culture inoculation and during fermentation. An aliquot (10% culture volume) of a 18 h culture in the same media was used to inoculate the fermentations. Cultures were incubated at constant temperature with stirring at 200 rpm. pH was controlled at 7.4 by automatic addition of 10% NaOH or 2 M HCl. For celluxanthene extraction, 2% XAD 16 adsorber resin was added. Most celluxanthenes were extracted from *C. thermocellum* DSM 4150 cultures grown in MJ defined medium[61] with 9 g L^{-1} glucose and 3 g L^{-1} cellobiose at 55 °C. Only celluxanthene B (**2**) was extracted from *Pseudobacteroides cellulosolvens* DSM 2933 culture grown in M520 at 37 °C.

General analytical procedures

NMR spectra were measured on Bruker Avance DRX 500 MHz or 600 MHz spectrometers with cryo probe in DMSO-d_6 or CD_2Cl_2. Spectra were referenced to the residual solvent peak DMSO-d_6 (δ_H = 2.49 ppm, δ_C.39.5 ppm) and CD_2Cl_2 (δ_H = 5.32 ppm, δ_C.54.0 ppm). Methods used in this study are shown below.

For 17-methoxycelluxanthene A (**6**):
gROESYes: Phase-sensitive 2D ROESY with ES (excitation sculpting) using 180 water-selective pulse (roesyesgpph).
DQF-COSY: Phase-sensitive 2D COSY with DQF (cosydfph).
gCOSYmqf: Magnitude-mode ge-2D COSY with multiple-quantum filter (cosygpmfqfvg3)
gHMBC: Magnitude-mode ge-2D HMBC (hmbcgpndqf).
gHSQCme: Phase-sensitive ge-2D multiplicity-edited HSQC using PEP (Preservation of Equivalent Pathway) and adiabatic inversion and refocusing pulses with gradients in back-inept (hsqcedetgpsisp2.2).
gDEPTQ: DEPTQ for quaternary carbons using gradients, adiabatic pulses and NOE (deptqgpsp.2).

For celluxanthenes A–D (**1–4**):
gCOSY: Magnitude-mode ge-2D COSY (cosygpqf)

gHSQC: Phase-sensitive ge-2D HSQC using echo-antiecho (hsqcetgp)
gHMBClp: Magnitude-mode ge-2D HMBC using low-pass J-filter (hmbcgplpndqf)
ROESY: Phase-sensitive 2D T-ROESY with presaturation (roesyphpr.2)
LC-HRMS measurements were performed using an Exactive Q Orbitrap high performance benchtop device with an electrospray ion source and an Accela HPLC system (Thermo Fisher Scientific, Bremen, Germany) consisting of an Autosampler equipped with a column oven, a 1250 Pump and a PDA Detector.

Isolation of celluxanthenes

Large scale cultivation and isolation of celluxanthines were performed many times to get better NMR spectral data due to instability and impurities. Here we only described the isolation procedures to be shown in NMR spectral data for celluxanthene A–D (**1–4**).

Orange colored XAD16 resins obtained from *Acetivibrio thermocellus* DSM1313 (5 L × 3) was extracted with methanol (600 mL) in an ultrasonic bath at room temperature for 5 min. After glass-filtration, resins were extracted with methanol / acetone (300 mL / 300 mL) in an ultrasonic bath at room temperature for 5 min. The resins obtained by glass-filtration were further extracted with acetone (600 mL) in an ultrasonic bath at room temperature for 5 min and then filtered through glass-filter. All extracts were combined and concentrated under the reduced pressure. The residue was dissolved in ethyl acetate and water. Water phase was extracted with ethyl acetate further twice. Organic phase was dried over sodium sulfate and concentrated under the reduced pressure.

The extract was subjected to reversed-phase HPLC (Phenomenex synergi Fusion-RP, C18, particle size 5 µm, pore size 80 Å, 21.2 × 250 mm) with a gradient system, solvent A (MiliQ water containing 0.1% trifuloroacetic acid), solvent B (83% aq. acetonitirile), 60% B for 10 min, to 100% B in 20 min, and kept for 10 min, at a frow-rate 16 mL min^{-1} to yield crude celluxanthene A (**1**, 5.2 mg) and crude celluxanthene C (**3**, 2.8 mg). The dark orange solid containing **1** was subjected to reversed-phase HPLC (Nucleodur Sphinx RP, C18, particle size 5 µm, pore size 110 Å, 10 × 250 mm) with a gradient system, solvent A (MiliQ water containing 0.1% trifluoroacetic acid), solvent B (83% aq. acetonitrile), 50% B for 10 min, to 100% B in 25 min, and kept for 15 min, at a frow-rate 6 mL min^{-1} to yield **1** (800 µg). Another dark orange solid containing **3** was subjected to reversed-phase HPLC (Phenomenex Fusion-RP, C18, particle size 5 µm, pore size 80 Å, 10 × 250 mm) with a gradient system, solvent A (MiliQ water containing 0.1% trifluoroacetic acid), solvent B (83% aq. acetonitrile), 50% B for 10 min, to 100% B in 25 min, and kept for 15 min, at a frow-rate 6 mL min^{-1} to yield **3** (600 µg).
Orange colored XAD16 resins obtained from *Acetivibrio thermocellus* DSM4150 (5 L) was extracted with the same method as above (solvent volume; each 200 mL). The two steps reversed-phase HPLCs were performed by the same procedure as those of **1** purification to yield celluxanthene D (**4**, 540 µg).

Orange colored XAD16 resins obtained from *Pseudobacteriodes cellulosolvens* DSM2933 (5 L) was extracted with the same method as above (solvent volume; each 200 mL). The two steps reversed-phase HPLCs were performed by the same procedure as those of celluxanthene A (**1**) purification to yield celluxanthene B (**2**, 400 µg).

Celluxanthene A (**1**): dark orange solid. HR-ESIMS, *m/z* 406.1444 $(M - H)^-$ (calcd. for $C_{27}H_{20}NO_3$: 406.1438), *m/z* 408.1587 $(M + H)^+$ (calcd. for $C_{27}H_{22}NO_3$: 408.1594).
Celluxanthene B (**2**): dark orange solid. HR-ESIMS, *m/z* 422.1396 $(M - H)^-$ (calcd. for $C_{27}H_{20}NO_4$: 422.1387) , *m/z* 424.1538 $(M + H)^+$ (calcd. for $C_{27}H_{22}NO_4$: 424.1543).

Celluxanthene C (**3**): dark orange solid. HR-ESIMS, *m/z* 432.1601 (M – H)⁻ (calcd. for $C_{29}H_{22}NO_3$: 432.1594) , *m/z* 434.1743 (M + H)⁺ (calcd. for $C_{29}H_{24}NO_3$: 434.1751).
Celluxanthene D (**4**): dark orange solid. HR-ESIMS, *m/z* 448.1554 (M – H)⁻ (calcd. for $C_{29}H_{22}NO_4$: 448.1543) , *m/z* 450.1695 (M + H)⁺ (calcd. for $C_{29}H_{24}NO_4$: 450.1700).

Methylation of celluxanthene A (1)

To crude celluxanthene A (**1**, 2.5 mg) in dry MeOH (500 µL), trimethylsilyldiazomethane (2.2 mL, 2 M in ether) was added and then stirred at room temperature for 2 h. After the reaction was quenched with 20 µL of acetic acid, the solvent was removed under the nitrogen gas stream. The residue was subjected to reversed-phase HPLC (Phenomenex fusion RP, 21.2 × 250 mm) using a gradient system; solvent A (MiliQ water containing 0.1% TFA), solvent B (acetonitrile), 50% B for 10 min, to 100% B in 25 min, and then kept for 10 min, at a flow rate 16 mL min^{-1} to yield crude 17-methoxy celluxanthene A. This yellow material was subjected to Chromabond SiOH (500 mg) and resin was washed with dichloromethane and eluted with dichloromethane / methanol to yield 17-methoxycelluxanthene A (**6**, 200 µg).

Labeling studies/feeding experiments

For labelling studies *C. thermocellum* DSM 4150 was used and grown in MJ defined medium. 46.5 mL of MJ medium was supplemented with 1 mL of 5 mg mL^{-1} of the investigated labelled substrate ([2,3,5,6-D_4]-*p*-hydroxybenzoic acid, [D_7]-phenylacetic acid, [1,3-$^{13}C_2$]-malonic acid, [1,2-$^{13}C_2$]-acetic acid), inoculated with 2.5 mL of a *C. thermocellum* culture at OD_{600}=0.6 and grown at 55 °C for 24 h. To a 2 L fermentation of *C. thermocellum* DSM 4150 in MJ defined medium[2], 5 mL of a sterile filtered solution of [$^{13}C_{11}$]-L-tryptophane (50 mg mL^{-1} in water) was supplemented.Ethyl acetate extracts were analyzed for the appearance of stable isotope labeled celluxanthenes.

Plasmid construction

For plasmid construction and storage *Escherichia coli* TOP10 as well as the *dcm*⁻ strain *E. coli* NEB Express were used and grown in lysogeny broth (LB) shaken at 150 rpm or on LB agar plates at 30 °C with appropriate antibiotic selection (chloramphenicol, 25 µg mL^{-1}; ampicillin, 100 µg mL^{-1}).
PCR was done using Phusion HF DNA polymerase. All plasmid constructions were done using the NEBuilder HiFi DNA Assembly Master Mix. For the amplification of homologous regions, the genomic DNA of *C. thermocellum* DSM1313 was used. All plasmids were verified by sequencing.
The plasmid construction was started from pDGO-68. First, the *hpt* gene was removed by *Xma*I/*Stu*I digest and replaced by a multiple cloning site. This was done by assembling of the digested plasmid with the self-annealed primers JK516 and JK517. The obtained plasmid was then cut with *Eco*RV to introduce the F1 region obtained using primers JK518/JK519. Next, this plasmid was *Pvu*II digested and assembled with the F2 region that was amplified with primers JK520/JK521. In the meantime, we realized that the *tdk* gene (needed for counter selection) in pDGO-68 has been mutated and thus inactivated. Therefore, we transferred the F1-cat-F2-cassette to pDGO145, a medium copy derivative with an active *tdk* gene. This was done by *Aat*II/*Pvu*II digest of the subcloning vector as well as of pDGO145 followed by ligation using T4 DNA ligase. The ligation reaction was directly transferred to the *dcm*-negative strain *E. coli* NEB Express. The final plasmid was named pCth-KS-ko (Fig. S72A).

Preparation of *C. thermocellum* mutant strain

C. thermocellum was genetically manipulated by homologous recombination to inactivate the PKS gene (CLO1313_RS10625) putatively involved in celluxanthene biosynthesis by chromosomally integrating a chloramphenicol (CatR) resistance marker cassette. *C. thermocellum* transformation was performed as described by Olson DG and Lynd LR.[60] Obtained colonies were analyzed by colony PCR to check for a double cross over event using OneTaq 2xMasterMix at an annealing temperature of 55 °C. First, arm A was checked using primers JK620 and PgapD-seq_rv. In parallel, arm B was amplified by primers JK621 and Cth-cat-seq_fw. Only the mutant would result in a product of 1143 bp and 1408 bp, respectively. For final verification, so far positive colonies were analyzed using primers JK620 and JK621. The mutant would give 3548 bp while the wildtype would result in 3009 bp. This PCR product was also used for sequencing. In addition, the mutant was also checked for wildtype contamination using primers JK620 and JK676 (1561 bp).

Cultivation of additional anaerobic strains for potential celluxanthene production in 1L-fermenter

All small scale and pre-cultures were grown under an anaerobic atmosphere (N_2:H_2:CO_2, 85:5:10 vol:vol:vol) in a Whitley A35 anaerobic work station (Don Whitley Scientific) operating at 37 °C. Cultures grown at a different temperature were prepared within the chamber. Schott bottles used for this purpose were filled by max. 50%, sealed with rubber stoppers, and incubated without gas exchange outside of the chamber in a standard incubator. While pre-cultures were grown only on cellobiose, all fermenters were additionally supplemented with 0.05% cellulose in case it is needed to induce pigment production. Also, 2% XAD 16 resin was added for celluxanthene extraction. Cultures were grown until no NaOH was needed to keep initial pH. This indicates that no acid is produced and thus strains are not actively growing anymore.

Clostridium straminisolvens DSM 16021 (50 °C) as well as *Acetivibrio mesophilus* DSM 107956 (45 °C) were cultivated in M122. *Acetivibrio saccincola* DSM 101079 was grown in M255 at 55 °C. Same media as well as M122 was used for *Ruminiclostridium herbifermentans* DSM 109966 (45 °C), and *Ruminiclostridium sufflavum* DSM 19573 (35 °C). *Acetivibrio clariflavus* DSM 19732 (55 °C), *Acetivibrio cellulolyticus* DSM 1870 (37 °C), and *Pseudobacteroides cellulosolvens* DSM 2933 (37 °C) were all grown in M520. M436 was used for *Acetivibrio alkalicellulosi* DSM 17461 (37 °C), also grown in M1036, and *Ruminococcus flavefaciens* DSM 25089 (37 °C).

Phylogenetic analysis

The Keto synthase (KS) proteins were downloaded from NaPDos2[62] (https://npdomainseeker.sdsc.edu/napdos2/napdos_home_v2.html) and extracted from the NCBI server (https://www.ncbi.nlm.nih.gov/) additionally. The amino acid sequences of these proteins were aligned using the MAFFT[63] server (https://mafft.cbrc.jp/alignment/server/) with MAFFT ver. 7 using the default setting besides Scoring matrix for amino acid sequences; JTT100, Mafft-homologs; ON. Unrooted fast maximum-likelihood-based tree was generated using IQ-TREE[64] (https://www.hiv.lanl.gov/content/sequence/IQTREE/iqtree.html) with automatic model selection mode (ModelFinder) resulting in the amino acid replacement model LG+F+R5. Phylogenetic bootstrap analysis was performed by ultrafast approximate bootstrap with 1000 bootstrap replicates.[65] The tree was displayed using MEGA7.[66]

Determination of cellulose consumption in 1L-fermenter

In general, fermentation of wild-type and PKS mutant strain of *C. thermocellum* DSM 1313 was performed as described above (Fermentation for isolation of celluxanthenes). However, some slight changes were done in order to determine growth on and consumption of cellulose. First, precultures were grown anaerobically in 150 mL modified CTFUD-rich medium supplemented with cellobiose in sealed Schott bottles at 55 °C without shaking. When reaching an OD_{600} of approx. 0.9, three aliquots of 50 mL each were used to inoculate the degassed fermenters filled with 950 mL modified CTFUD-rich medium that contained 10 g L^{-1} cellulose as only carbon source. Cultures were grown for 96 h at 55 °C and stirring at 200 rpm. From each fermenter, three times approx. 10 mL culture were taken at 0 h and every 24 h. The exact volume was noted. After centrifugation for 10 min at 7500 × g the pellet containing cells and cellulose was treated with 5 mL of 10% formic acid (99%) for 1 h at 100 °C and allowed to cool down to room temperature. The suspension was filtered through a 0.45 µM nylon membrane with a disk diameter of 47 mm from GVS Magna (Fisher Scientific) and washed twice with 20 mL MilliQ water. The filter together with the undigested cellulose was dried completely at 105 °C. After cool down to room temperature, the cellulose carrying filters were weight at a precision scale. The average weight of 20 filters was subtracted from each sample. For each fermenter, the average result from first measurements (0 h) were used as initial cellulose concentration. The consumed cellulose (in g L^{-1}) was calculated as difference of this individual initial cellulose concentration and the remaining cellulose concentration for each day and sample.

Determination of bioactivity

Initial qualitative antibacterial and antifungal profiling of celluxanthene A (**1**) was carried out in an agar diffusion assay as previously described.[67] In addition, **1** was quantitatively tested by determination of minimal inhibitory concentration (MIC) against *M. vaccae* and MRSA.[68]

Supplemental Tables

Table S1. ^{1}H (600 MHz) and ^{13}C (150 MHz) NMR Data of celluxanthene A (**1**) in DMSO-d_6.

Position	^{1}H (ppm), J = Hz	^{13}C (ppm)	^{1}H-^{1}H COSY (^{1}H to ^{1}H)	HMBC (^{1}H to ^{13}C)
1		174.2		
2		90.7*		
3	6.28 (d 15.3)	127.1	H4	C1, C17
4	6.89 (m)	119.3	H3, H5	C2, C6
5	6.37 (dd 14.5, 11.6)	138.0	H4, H6	C3, C7
6	6.12 (m)	125.7	H5, H7	C4, C8
7	6.46 (dd 14.6, 11.3)	135.5	H6, H8	C5, C9
8	6.29 (m)	128.9	H7, H9	C6, C10
9	6.95 (dd 15.4, 10.8)	130.1	H8, H10	C7, C11
10	6.49 (d 15.4)	129.6	H9	C8, C12, C16
11		137.6		
12, 16	7.42 (d 7.6)	125.9	H13, H15	C10, C14
13, 15	7.30 (t 7.6)	128.6	H12, H14, H16	C13, C14, C15
14	7.17 (t 7.6)	126.9	H 13, H15	C12, C13, C15, C16
17		171.1		
18		145.6		
19	6.27 (s)	92.4		C17, C18, C22
20	7.69 (s)	126.4		C19, C21, C22, C27
20-NH	11.39 (s)			C21, C22, C27
21		109.4		
22		126.5		
23	7.79 (d 7.6)	118.5	H24	C21, C24, C27
24	7.06 (t 7.6)	119.3	H23, H25	C22, C26
25	7.12 (t 7.6)	121.5	H24, H26	C23, C26, C27
26	7.39 (d 7.6)	111.6	H25	C22, C24
27		135.7		

*: determined by HMBC.

Table S2. ^{1}H (700 MHz) and ^{13}C (176 MHz) NMR Data of 17-methoxycelluxanthene A (**6**) in CD_2Cl_2.

Position	^{1}H (ppm), *J* = Hz	^{13}C (ppm)	^{1}H-^{1}H COSY (^{1}H to ^{1}H)	HMBC (^{1}H to ^{13}C)
1		168.0		
2		103.9		
3	6.63 (d 15.5)	119.8	H4	C1, C2, C5, C17
4	7.39 (dd 15.9, 11.7)	133.3	H3, H5	C2, C6
5	6.43 (dd 14.8, 11.3)	134.7	H4, H6	C3, C7
6	6.53 (dd 14.1, 10.1)	135.1	H5, H7	C8
7	6.49 (m)	134.5	H6	
8	6.51 (dd 14.3, 10.2)	134.6	H9	C10
9	6.91 (dd 15.7, 9.7)	129.6	H8, H10	C7, C11
10	6.61 (d 15.7)	133.3	H9	C12, C16
11		137.9		
12, 16	7.42 (d 7.9)	127.0	H13, H15	C10, C12, C14, C16
13, 15	7.32(t 7.9)	129.2	H12, H14, H16	C11, C13, C15
14	7.22 (m)	128.1	H13, H15	C12, C13, C15, C16
17		161.8		
17-*O*Me	4.28 (s)	61.5		C17
18		140.2		
19	6.66 (s)	101.4		C17, C18, C20, C22
20	7.98 (s)	128.6		C19, C22, C27
20-NH	8.72 (s)			
21		110.5		
22		127.1		
23	7.81 (d 7.7)	119.0	H24	C22, C25, C27
24	7.23 (t 7.7)	121.5	H23, H25	C23, C26
25	7.27 (t 7.7)	123.5	H24, H26	C24, C27
26	7.46 (d 7.7)	112.1	H25	C23, C25
27		136.1		

Table S3. ^{1}H (600 MHz) and ^{13}C (150 MHz) NMR Data of celluxanthene B (**2**) in DMSO-d_6.

Position	^{1}H (ppm), J = Hz	^{13}C (ppm)	^{1}H-^{1}H COSY (^{1}H to ^{1}H)	HMBC (^{1}H to ^{13}C)
1		168.4		
2		98.6		
3	6.43 (d 15.9)	121.3	H4	C1, C2, C5, C17
4	7.09 (m)	128.6	H3	C2, C5, C6
5	6.38 (m)	134.6		
6	6.38 (m)	132.55*		
7	6.40 (m)	132.51*		C5, C9
8	6.39 (m)	133.7	H9	C10
9	6.76 (m)	126.8	H8, H10	C7
10	6.50 (d 15.6)	132.9	H9	C8, C12, C16
11		128.8		C9, C13, C15
12, 16	7.28 (d 8.8)	128.2	H13, H15	C10, C12, C14, C16
13, 15	6.72	116.1	H12, H16	C11, C13, C15
14		157.7		
14-OH	9.58 (s)			C13, C14, C15
17		164.9**		
18		140.2		
19	6.83 (s)	100.5		C17, C18, C20, C22
20	7.84 (d 2.6)	129.1	20-NH	C21, C22, C27
20-NH	11.73 (brs)		H20	C20, C21, C22, C27
21		109.2		
22		126.9		
23	7.81 (d 7.8)	118.9	H24	C21, C25, C27
24	7.13 (t 7.8)	120.7	H23	C22, C26
25	7.13 (t 7.8)	122.7	H26	C23, C27
26	7.45 (d 7.8)	112.6	H25	C22, C24
27		136.4		

*exchangeable. **: determine by HMBC.

Table S4. ^{1}H (600 MHz) and ^{13}C (150 MHz) NMR Data of celluxanthene C (**3**) in DMSO-d_6.

Position	^{1}H (ppm), J = Hz	^{13}C (ppm)	^{1}H-^{1}H COSY (^{1}H to ^{1}H)	HMBC (^{1}H to ^{13}C)
1		177.8		
2		90.8		
3	6.27 (d 14.9)	127.5	H4	C1, C5, C19
4	6.88 (dd 14.9, 11.3)	119.33	H3, H5	C2, C6
5	6.37 (m)	138.3	H4, H6	
6	6.09(dd 14.6, 11.2)	125.5	H5, H7	C4, C8
7	6.38 (m)	135.5	H6, H8	C5
8	6.23 (m)	128.68	H7, H9	C10
9	6.46 (dd 14.7, 11.2)	134.8	H8, H10	C7, C11
10	6.36 (m)	130.6	H9. H11	C8, C12
11	6.97 (dd 15.4, 10.9)	129.8	H10, H12	C9, C13
12	6.54 (d 15.4)	130.5	H11	C10, C14, C18
13		137.4		
14, 18	7.44 (d 7.6)	126.0	H15, H17	C12, C16
15, 17	7.30 (d 7.6)	128.65	H14, H16, H18	C13, C14, C15, C17, C18
16	7.18 (t 7.6)	127.1	H15, H17	C14, C18
19		171.3		
20		145.8		
21	6.23 (s)	92.0		C1, C20
22	7.68 (s)	126.3	22-NH	C23, C24, C29
22-NH	11.37 (s)		H22	C23, C24, C29
23		109.4		
24		126.5		
25	7.79 (d 7.8)	118.5	H26	C23, C27, C29
26	7.06 (td 7.5, 1.2)	119.29	H25, H27	C25, C28
27	7.11 (td 7.5, 1.2)	121.5	H26, H28	C24, C29
28	7.38 (d 8.0)	111.6	H27	C23, C25
29		135.7		

Table S5. ^{1}H (600 MHz) and ^{13}C (150 MHz) NMR Data of celluxanthene D (**4**) in DMSO-d_6.

Position	^{1}H (ppm), *J* = Hz	^{13}C (ppm)	^{1}H-^{1}H COSY (^{1}H to ^{1}H)	HMBC (^{1}H to ^{13}C)
1		168.1		
2		97.8		
3	6.43 (d 15.3)	121.3	H4	C1, C2, C5, C19
4	7.07 (dd 15.1, 9.7)	127.1	H3	C2, C5, C6
5	6.39 (m)	134.8		
6		131.7		
7	6.39 (m)	132.3		C9
8	6.37 (m)	133.2		C6
9	6.40 (m)	132.7		C11
10	6.38 (m)	133.5	H11	
11	6.76 (dd 15.4, 9.2)	126.3	H10, H12	C9, C13
12	6.51 (d 15.4)	132.3	H11	C10, C14, C18
13		128.3		
14, 18	7.28 (d 8.8)	127.7	H15, H17	C12, C14, C16, C18
15, 17	6.71 (d 8.8)	115.6	H14, H18	C13, C14, C15, C17, C18
16		157.3		
16-OH	9.60 (s)			C15, C16, C17
19		163.1		
20		142.3		
21	6.81 (s)	99.7		C19, C20, C22, C24
22	7.83 (d 2.7)	128.3	22-NH	C23, C24, C29
22-NH	11.72 (s)		H22	C23, C24, C29
23		108.8		
24		126.4		
25	7.81 (d 7.9)	118.4	H26	C23, C27, C29
26	7.13 (t 7.9)	120.2	H25, H27	C24, C28
27	7.17 (t 7.9)	122.2	H26, H28	C25, C29
28	7.44 (d 7.9)	112.1	H27	C24, C26
29		135.9		

Table S6. Celluxanthene biosynthesis genes and genes in flanking regions.

Gene	Locus Tag	Length	Protein ID	Enzyme Annotation
trmL	CLO1313_RS10660	495 nt	WP_003512148.1	tRNA (uridine(34)/cytosine(34)/5-carboxymethylaminomethyluridine(34)-2'-O)- methyltransferase TrmL
dgc	CLO1313_RS10655	1518 nt	WP_003517873.1	diguanylate cyclase
cexA	CLO1313_RS10650	1035 nt	WP_003512152.1	ketoacyl-ACP synthase III
cexB	CLO1313_RS10645	246 nt	WP_003517875.1	acyl carrier protein
cexC	CLO1313_RS10640	1038 nt	WP_003512155.1	ketoacyl-ACP synthase III
cexD	CLO1313_RS10635	1485 nt	WP_003512157.1	acyl-CoA ligase
cexE	CLO1313_RS10630	972 nt	WP_003517877.1	alpha/beta hydrolase
cexF	CLO1313_RS10625	8265 nt	WP_003512162.1	type I polyketide synthase
cexG	CLO1313_RS10620	726 nt	WP_003512164.1	4'-phosphopantetheinyl transferase superfamily protein
gap	CLO1313_RS10615	1011 nt	WP_003512173.1	type I glyceraldehyde-3-phosphate dehydrogenase
pgk	CLO1313_RS10610	1185 nt	WP_173668647.1	phosphoglycerate kinase
tpiA	CLO1313_RS10605	756 nt	WP_003512174.1	triose-phosphate isomerase

Table S7. Keto synthase (KS) proteins used for Maximum Likelihood phylogenetic tree (besides KS proteins downloaded from NaPDos2).

Protein	Strain	Accession Number
Cex-Acce	*Acetivibrio cellulolyticus* CD2	WP_010250325.1
Cex-Accl	*Acetivibrio clariflavus* DSM 19732	WP_014255050.1
Cex-Acme	*Acetivibrio mesophilus* N2K1	WP_128705978.1
Cex-Acsa	*Acetivibrio saccincola* A7, GGR1	WP_105368633.1
Cex-Acst	*Acetivibrio straminisolvens* JCM 21531	WP_265444051.1
Cex-Acth	*Acetivibrio thermocellus* DSM 1313, AD2, YS, M3, LQRI, PAL5	WP_003512162.1
Cex-Beca	*Bellilinea caldifistulae* GOMI-1	WP_061917867.1
Cex-Caba	*Calditrichaeota bacterium*	RPI03472.1
Cex-Loar	*Longilinea arvoryzae* KOME-1	WP_075074776.1
Cex-Psce	*Pseudobacteroides cellulosolvens* DSM 2933	WP_036942210.1
Cex-Ruhe	*Ruminiclostridium herbifermentans* MA18	QNU66867.1
Cex-Rusu	*Ruminiclostridium sufflavum* DSM 19573	WP_110463164.1
ApeO-Xd	*Xenorhabdus doucetiae* FRM16, DSM 17909	CDG19162.1
ApeO-Ab7F28	*Acinetobacter baumannii* NCGM 237	WP_000681797.1
ArcK-Vp	*Variovorax paradoxus* B4	WP_021005014.1
ApeO-Fj	*Flavobacterium johnsoniae* UW101	WP_012023173.1

Table S8. Strains used in this study.

Strain	Genotype	Medium	Temp.	Source
Clostridium thermocellum	DSM 1313	CTFUD, MJ, M122	55 °C	DSMZ
Clostridium thermocellum Δ*cex*	Δ*CLO1313_RS10625*::Cm^R			This study
Clostridium thermocellum	DSM 4150	MJ	55 °C	DSMZ
Clostridium straminisolvens	DSM 16021	M122	50 °C	DSMZ
Acetivibrio alkalicellulosi	DSM 17461	M1036, M436	37 °C	DSMZ
Acetivibrio cellulolyticus	DSM 1870	M520	37 °C	DSMZ
Acetivibrio clariflavus	DSM 19732	M520	55 °C	DSMZ
Acetivibrio mesophilus	DSM 107956	M122	45 °C	DSMZ
Acetivibrio saccincola	DSM 101079	M255	55 °C	DSMZ
Pseudobacteroides cellulosolvens	DSM 2933	M520	37 °C	DSMZ
Ruminiclostridium herbifermentans	DSM 109966	M122, M255	45 °C	DSMZ
Ruminiclostridium sufflavum	DSM 19573	M122, M255	35 °C	DSMZ
Ruminococcus flavefaciens	DSM 25089	M436	37 °C	DSMZ

CmR – chloramphenicol resistance

Table S9. Plasmids used in this study.

Name	Description	Marker	Size	Reference
pDGO-68	basic plasmid for markerless deletion/insertion with *hpt*, requires Δ*hpt* background, contains (mutated/inactive) *tdk* for counterselection, pUC ori	AmpR, CmR	7.0 kb	[69]
pDGO145	basic plasmid for markerless deletion/insertion with *hpt*, requires Δ*hpt* background, contains *tdk* for counterselection, p15A-ori	AmpR, CmR	7.0 kb	[69]
pCth-KS-ko	knockout plasmid for KS (*CLO1313_RS10625*) in *C. thermocellum*	AmpR, CmR	8.4 kb	this study

AmpR – ampicillin resistance, CmR – chloramphenicol resistance

Table S10. Primers used in this study.

Primer	Sequence (5' → 3'), primer binding side, *RES*	Use
JK516	CTATTCAGGAATTGTCAGATAGGCCTAATGACTGGCTTTTAT AA*CCCGGGC**CTCGAG*AAAACAAAAGG	Deletion of *hpt* from pDGO145
JK517	CCTTTTGTTTT*CTCGAGGCCCGGG*TTATAAAAGCCAGTCATT AGGCCTATCTGACAATTCCTGAATAG	Deletion of *hpt* from pDGO145
JK518	GGCGTATCACGAGGCGA*TCTAGA*ATGAATGAAGCAAGC	Amplification of F1 region of *cexF*
JK519	CCATGCCTATTCCCAC*GATATC*ATTGGAAAGCTTCGGCAC	Amplification of F1 region of *cexF*
JK520	CATACCTGGCCCAGTAGTTCA*GGCGCC*TTCGCCGACCGGCTC	Amplification of F2 region of *cexF*
JK521	GATTTTTTCACTACTATTAG*CAGCTG*TCGGGTGCCACCGCTT CTC	Amplification of F2 region of *cexF*
JK620	TGAATTTCCGCGAAACAATG	Screening for *cexF* mutants
JK621	GTAAAGAGCCGCAACCTGAG	Screening for *cexF* mutants
JK676	GGTAATGCTCTATCTTTGC	Screening of *cexF* mutants for wild-type contamination
PgapD-seq_rv	CTGCACACAATATTCATCAG	Screening for *cexF* mutants
Cth-cat-seq_fw	ACCGCTATCTTTACAGGTAC	Screening for *cexF* mutants

Table S11. Inhibitory effects of celluxanthene A against several bacterial and fungal strains.

Strain	Zone of inhibition in mm		
	Celluxanthene A (1 mg mL^{-1}; 2.45 mM)	Ciprofloxacin (5 µg mL^{-1}; 3 mM) / Amphotericin B (10 mg mL^{-1}; 1.1 mM)	Methanol
Bacillus subtilis 6633 B1	11	28	0
Staphylococcus aureus 511 B3	10	17	0
Staphylococcus aureus 134/94 R9 (MRSA)	10	0	0
Mycobacterium vaccae 10670 M4	12	21p	0
Pseudomonas aeruginosa K799/61 B7	0	25	0
Escherichia coli 458 B4	0	25	0
Enterococcus faecalis 1528 R10 (VRE)	0	16	0
Sporobolomyces salmonicolor 549 H4	0	18p	10
Candida albicans H8	0	20	0
Penicillium notatum JP36 P1	0	17p	10

p – partial inhibition

Supplemental Figures

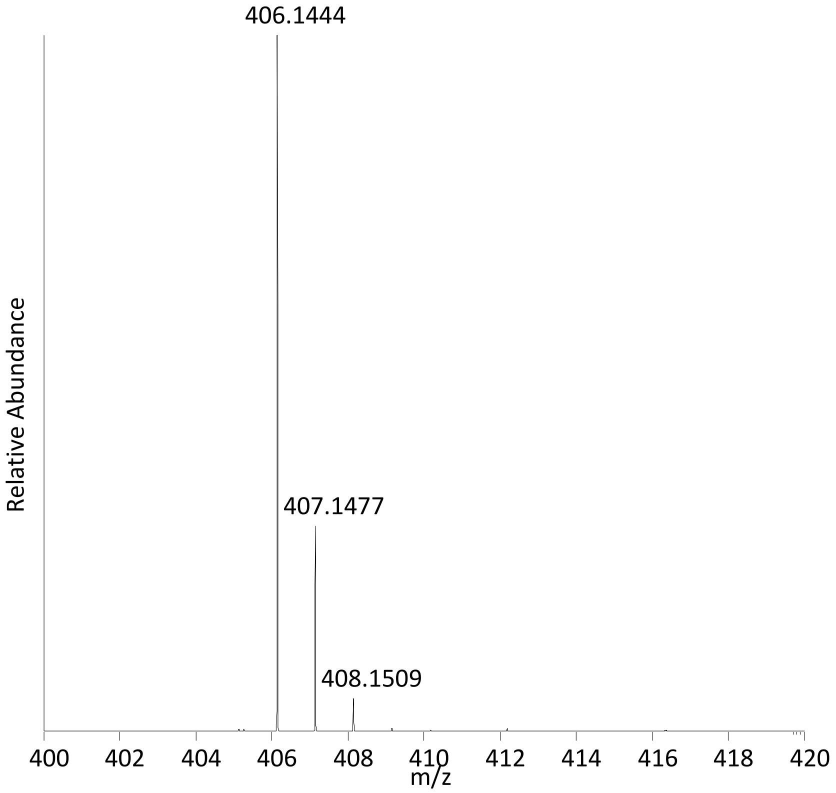

Figure S1. Negative HR-MS spectrum of celluxanthene A (**1**).

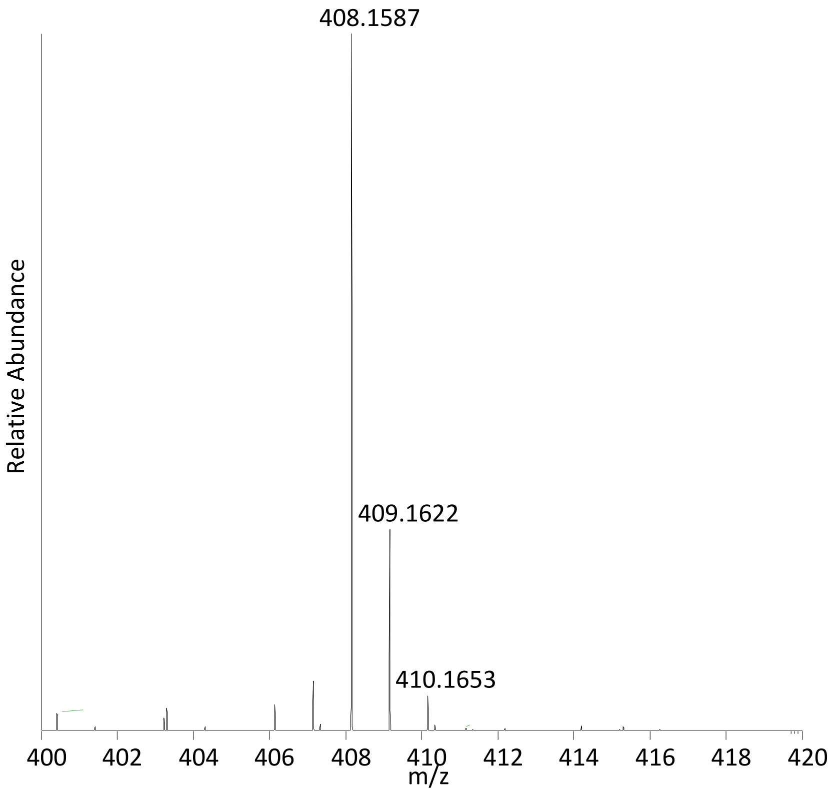

Figure S2. Positive HR-MS spectrum of celluxanthene A (**1**).

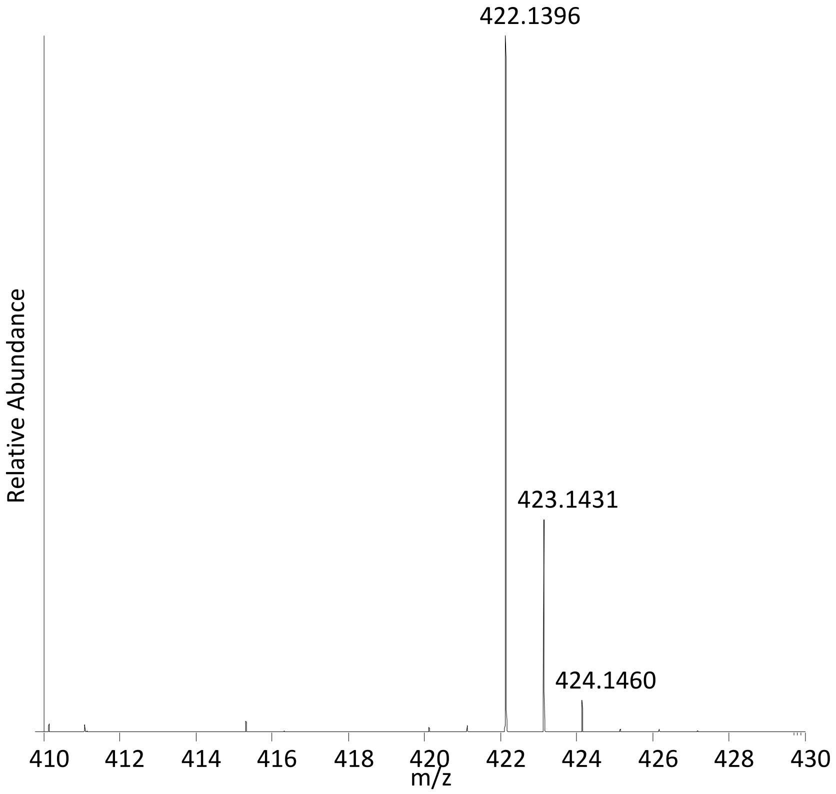

Figure S3. Negative HR-MS spectrum of celluxanthene B (**2**).

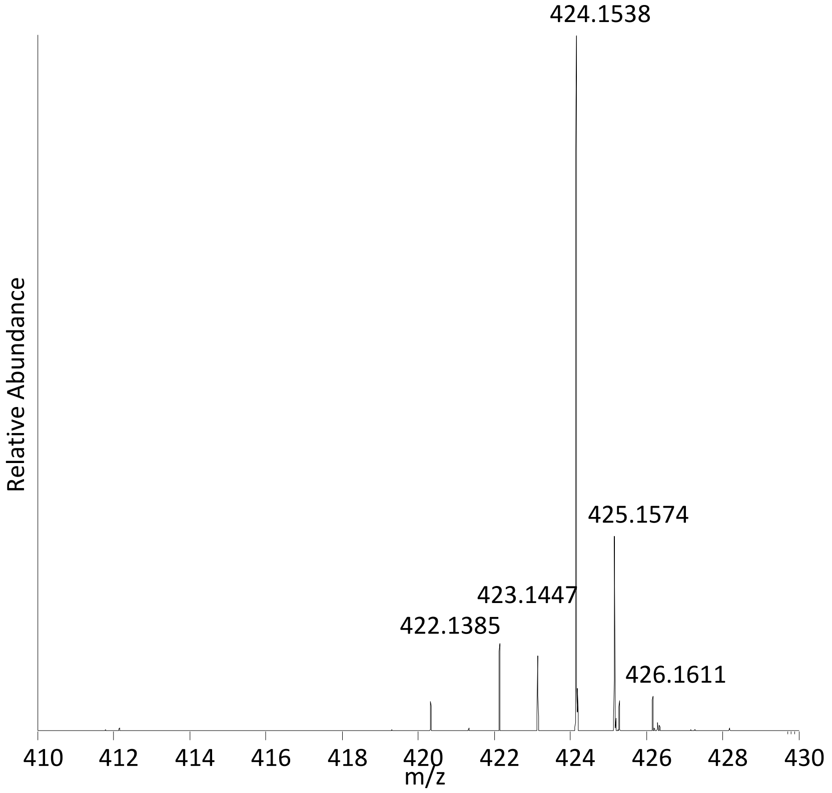

Figure S4. Positive HR-MS spectrum of celluxanthene B (**2**).

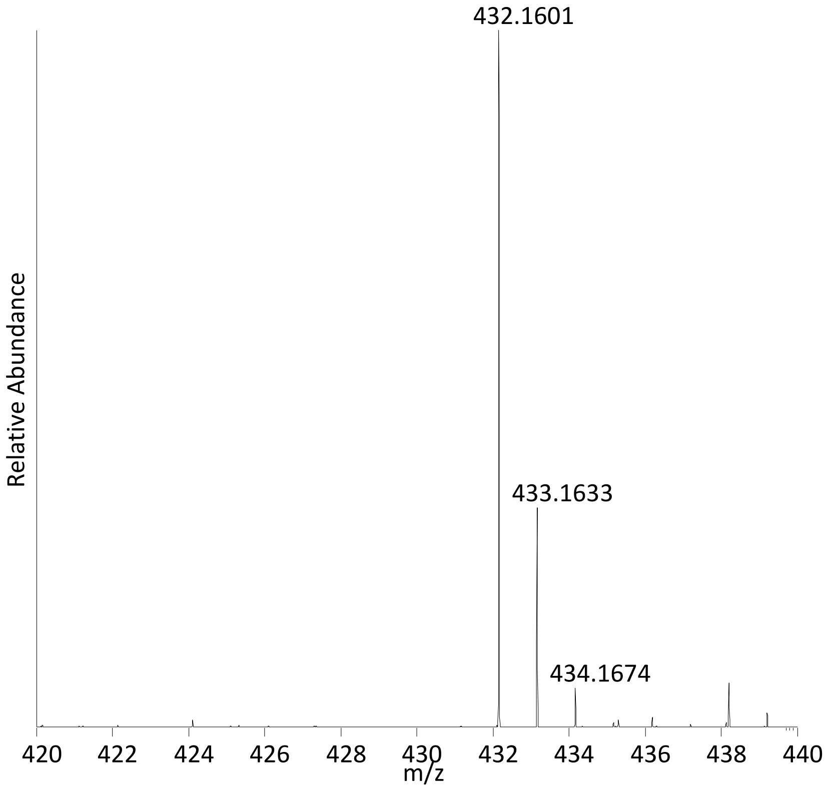

Figure S5. Negative HR-MS spectrum of celluxanthene C (**3**).

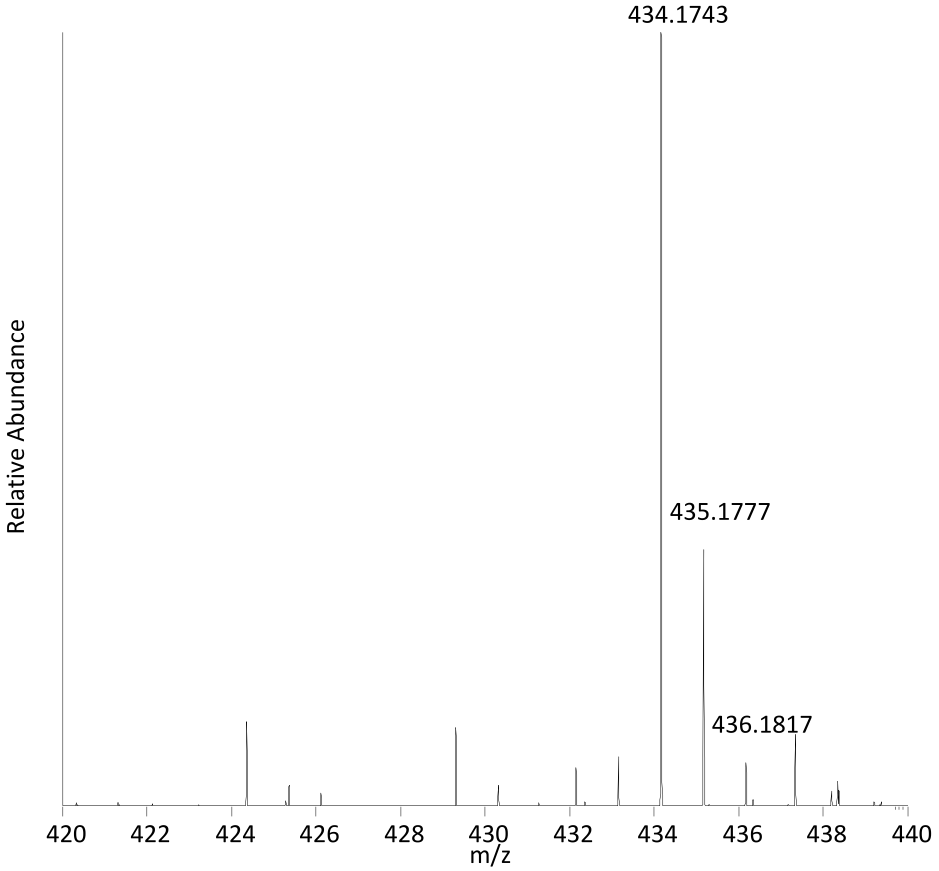

Figure S6. Positive HR-MS spectrum of celluxanthene C (**3**).

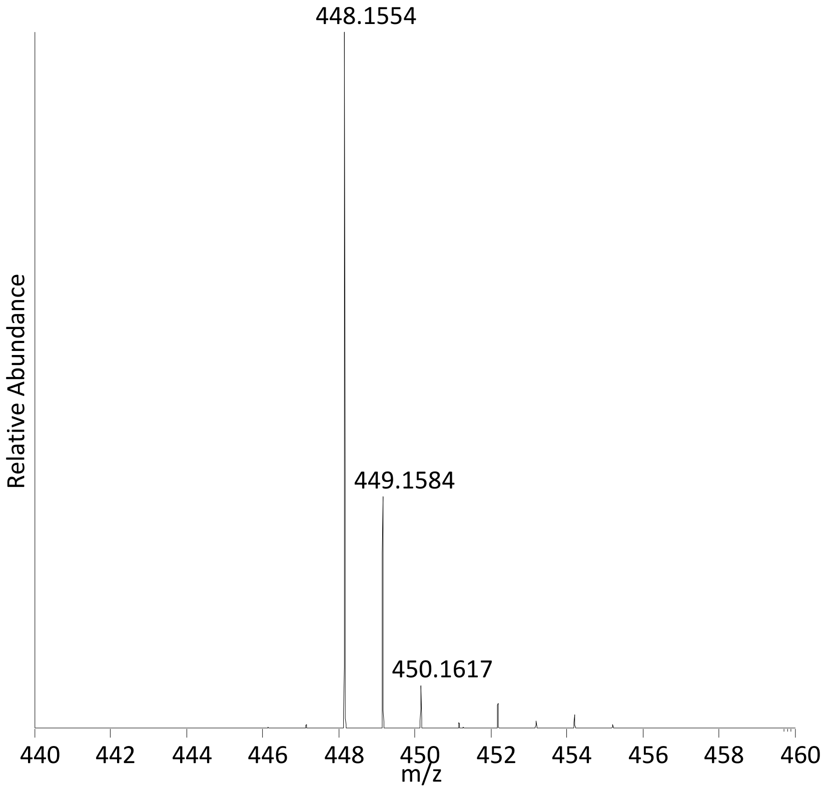

Figure S7. Negative HR-MS spectrum of celluxanthene D (**4**).

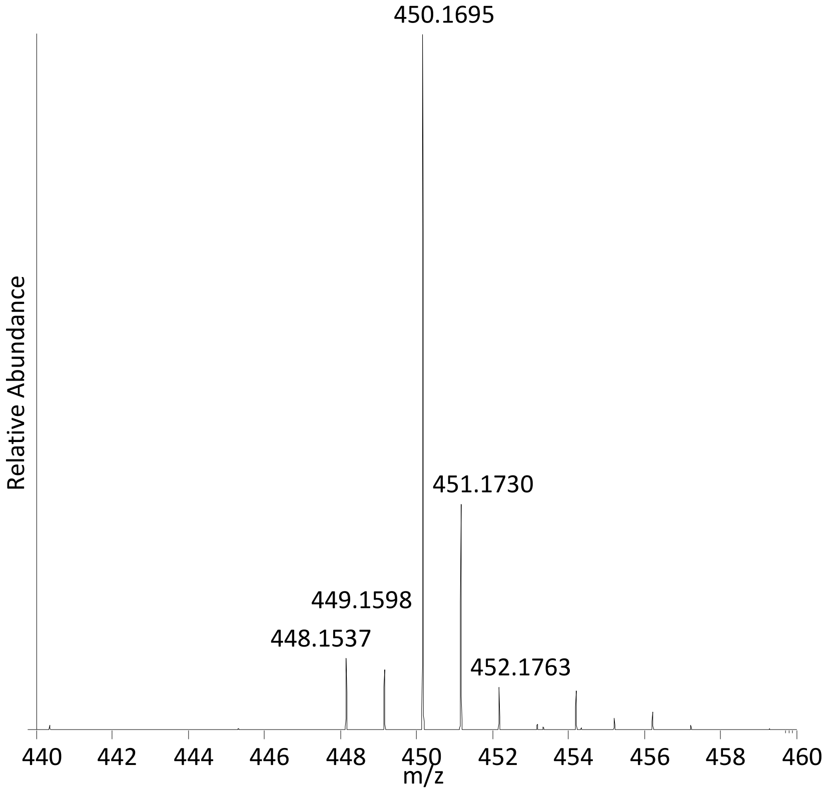

Figure S18. Positive HR-MS spectrum of celluxanthene D (**4**).

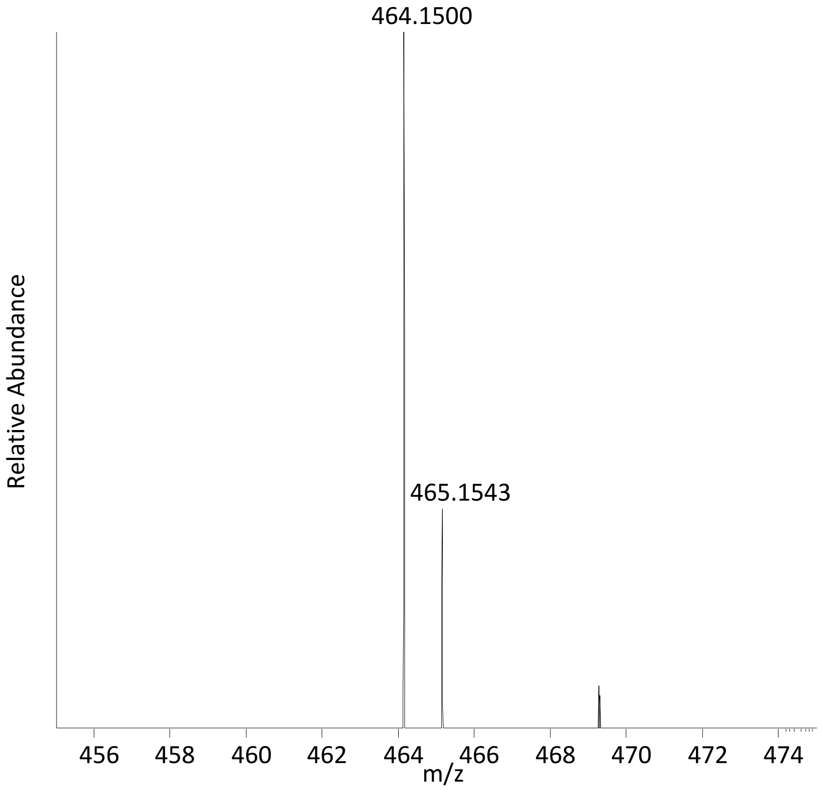

Figure S9. Negative HR-MS spectrum of celluxanthene E (**5**).

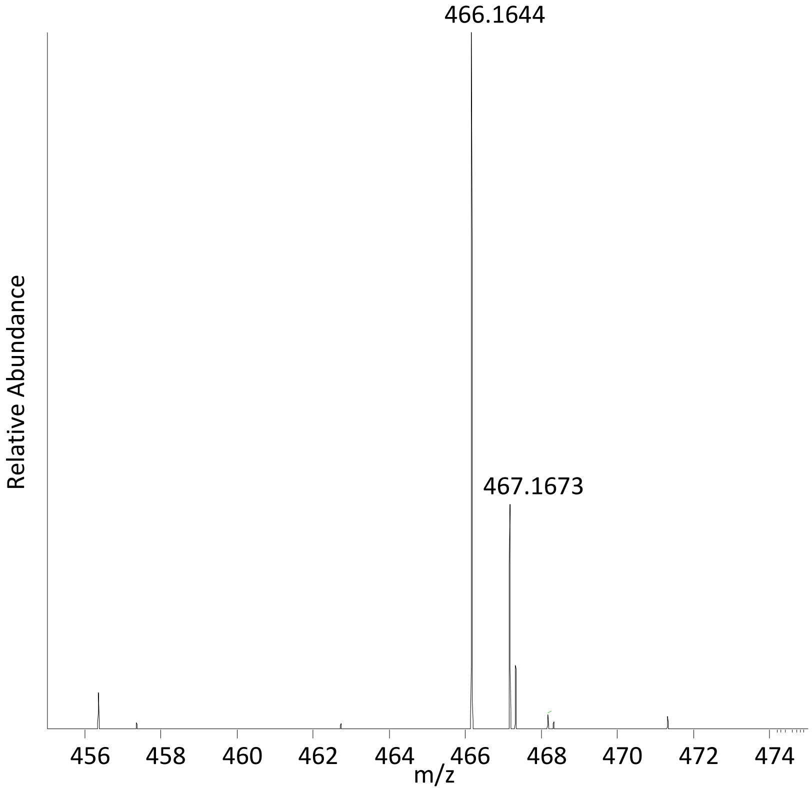

Figure S10. Negative HR-MS spectrum of celluxanthene E (**5**).

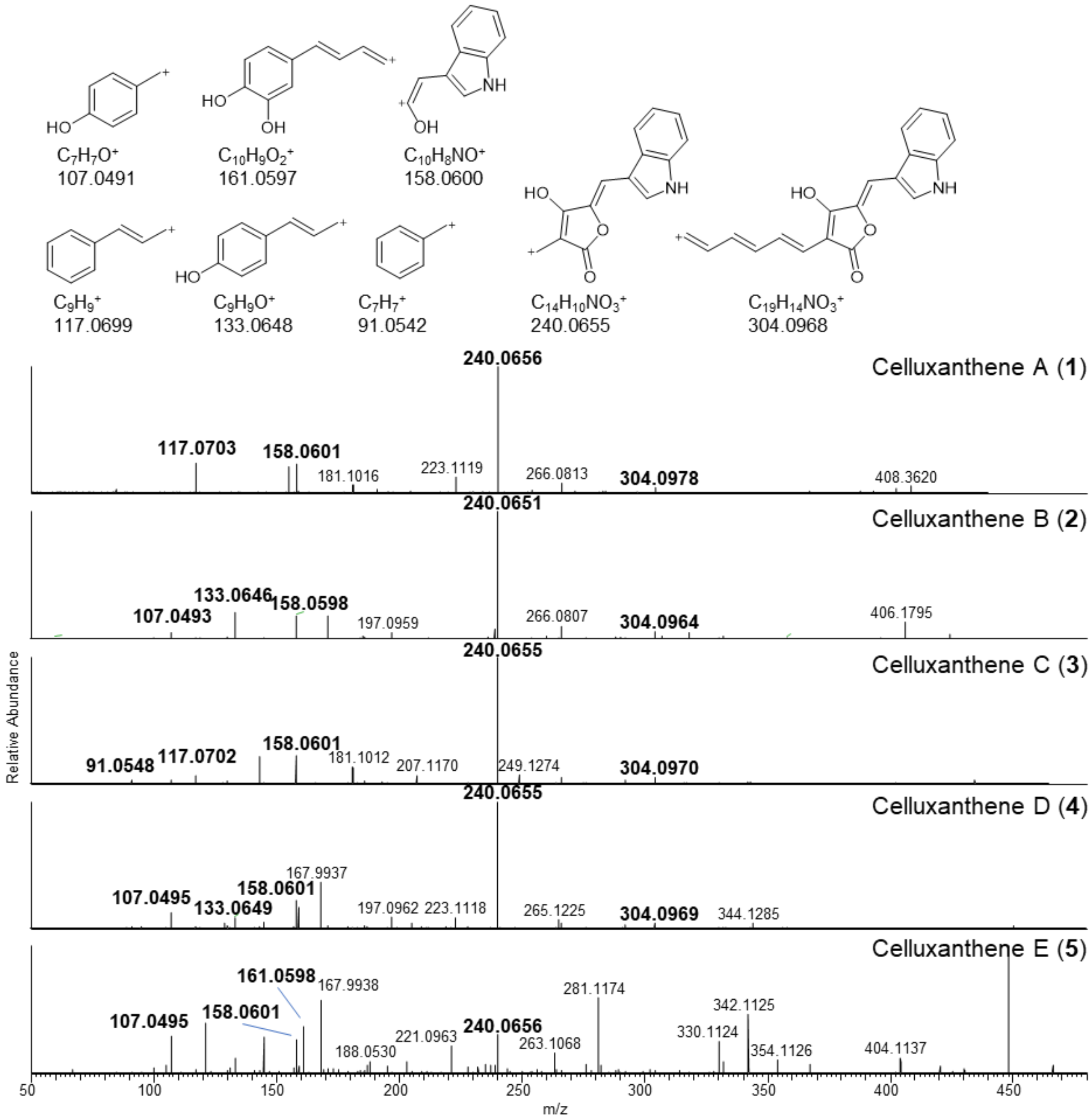

Figure S11. Positive HR-MS/MS profile of celluxanthene A–E (**1–5**).

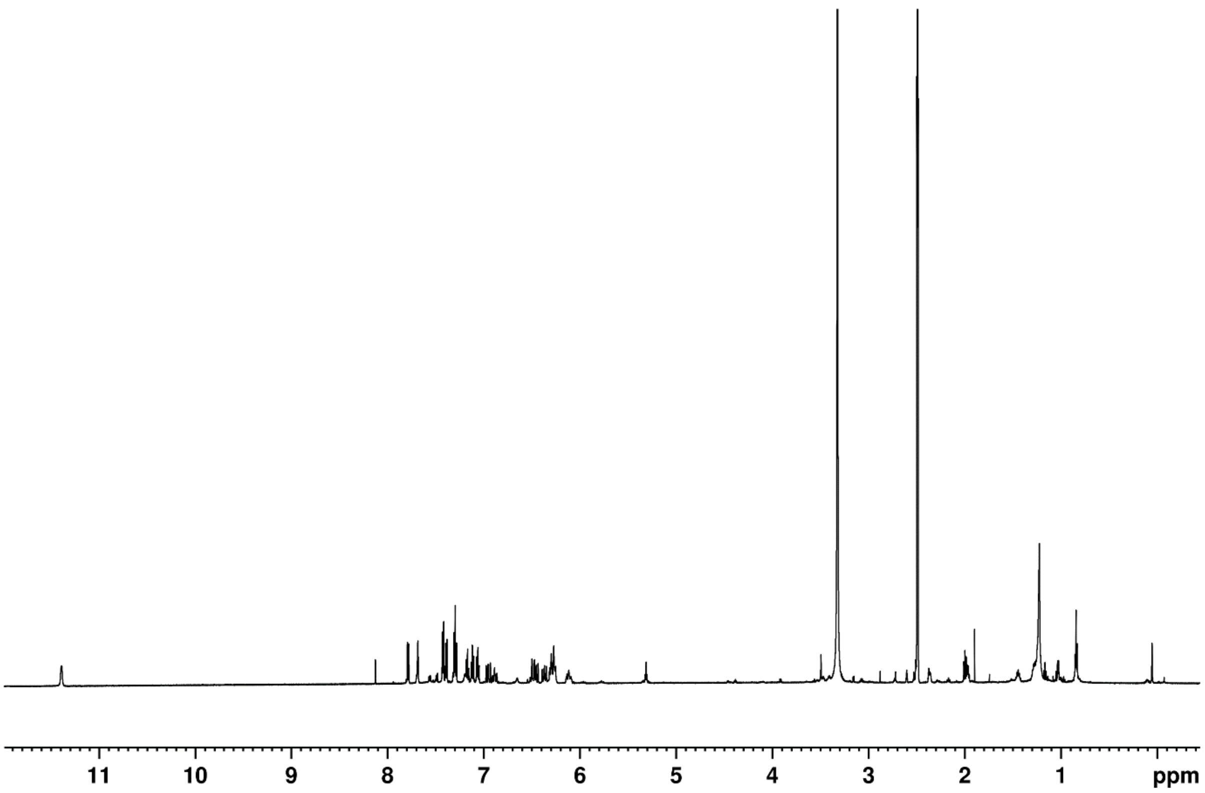

Figure S12. ^{1}H NMR spectrum of celluxanthene A (**1**) in DMSO-d_6 at 300K.

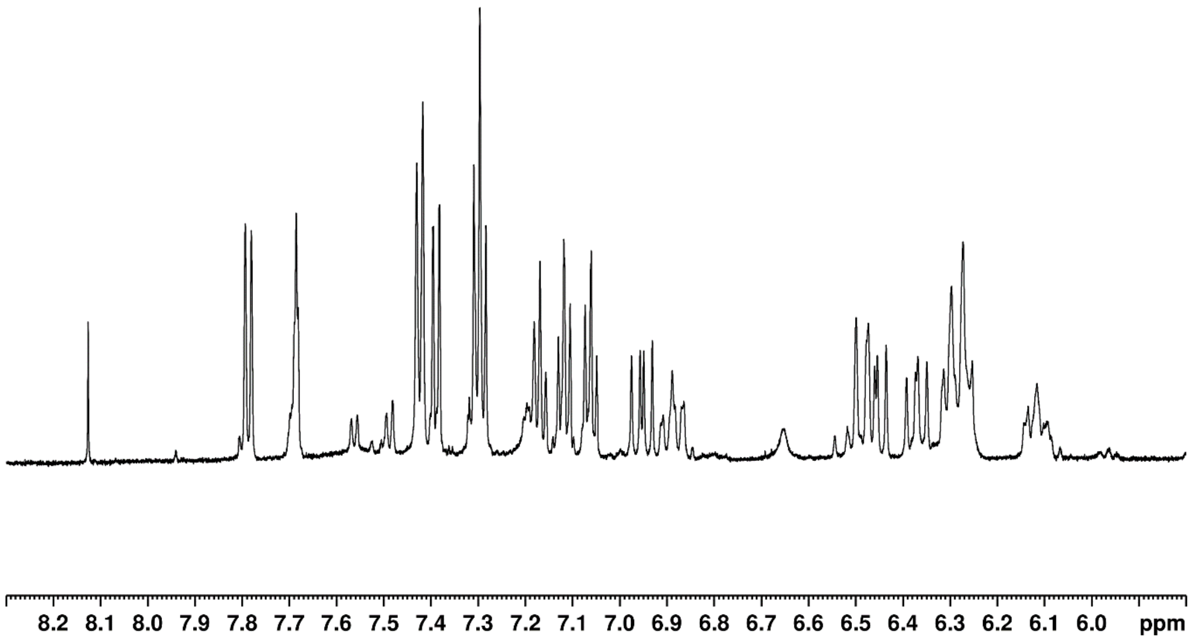

Figure S13. Extended ^{1}H NMR spectrum of celluxanthene A (**1**) in DMSO-d_6 at 300K.

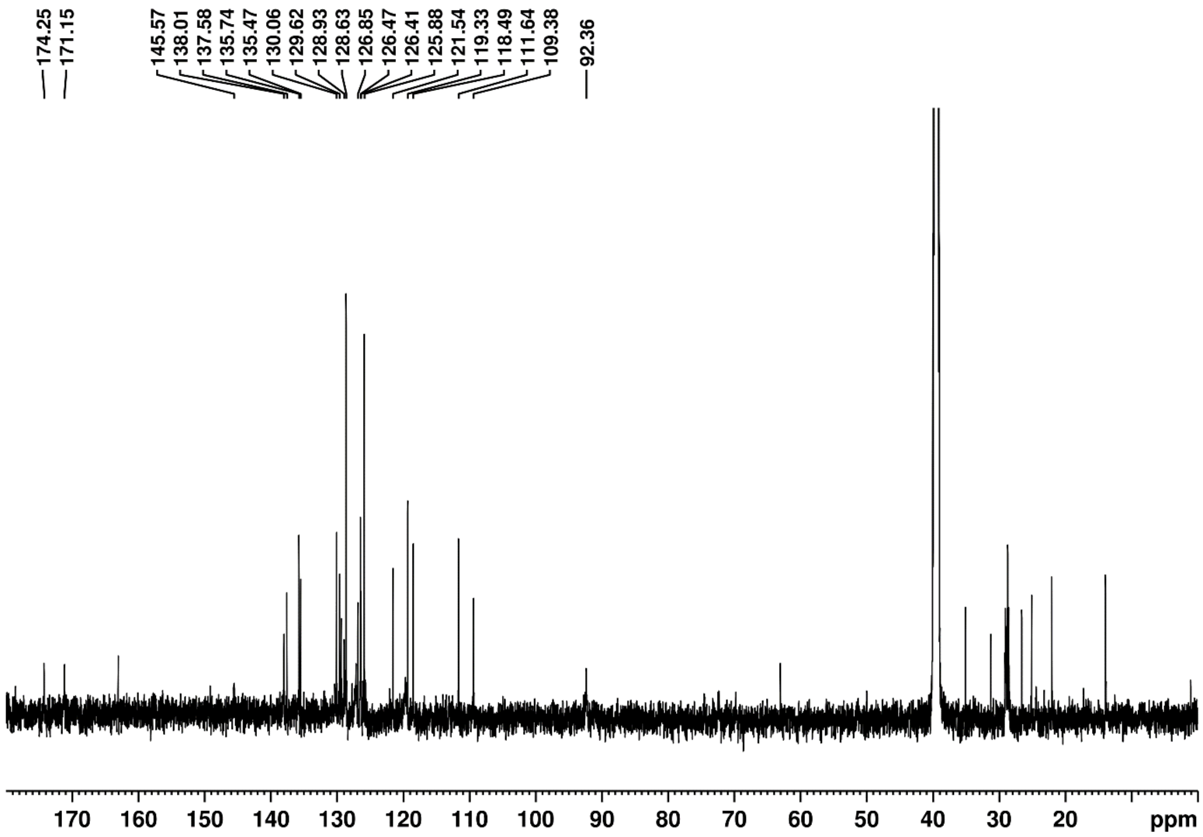

Figure S14. ^{13}C NMR spectrum of celluxanthene A (**1**) in DMSO-d_6 at 300K.

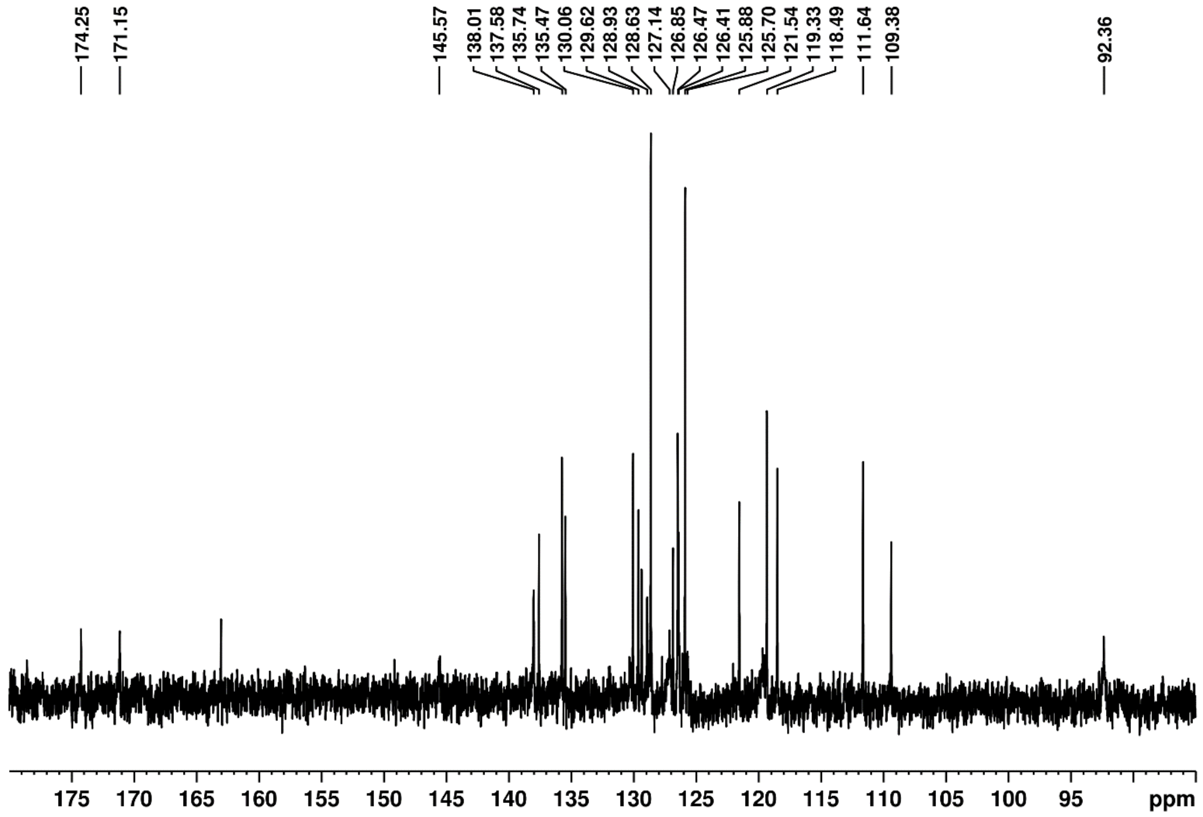

Figure S15. Extended ^{13}C NMR spectrum of celluxanthene A (**1**) in DMSO-d_6 at 300K.

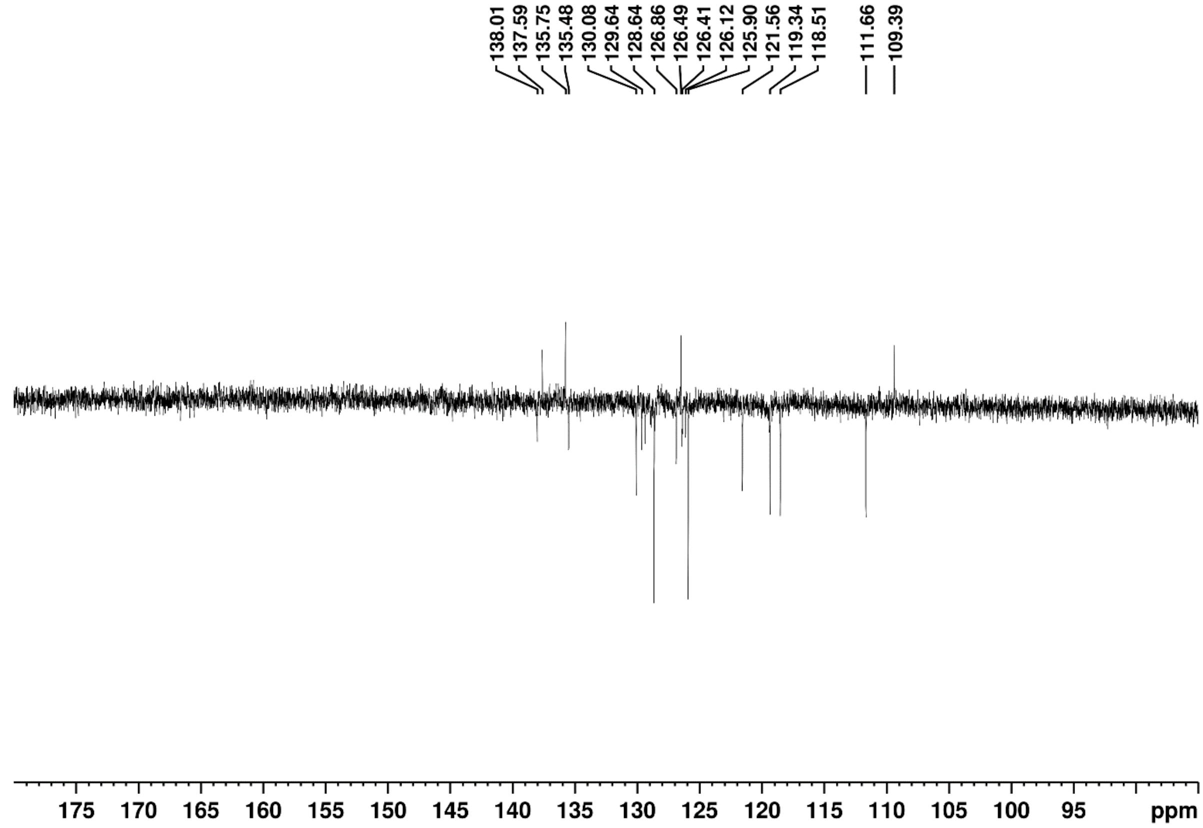

Figure S16. DEPTQ spectrum of celluxanthene A (**1**) in DMSO-d_6 at 300K.

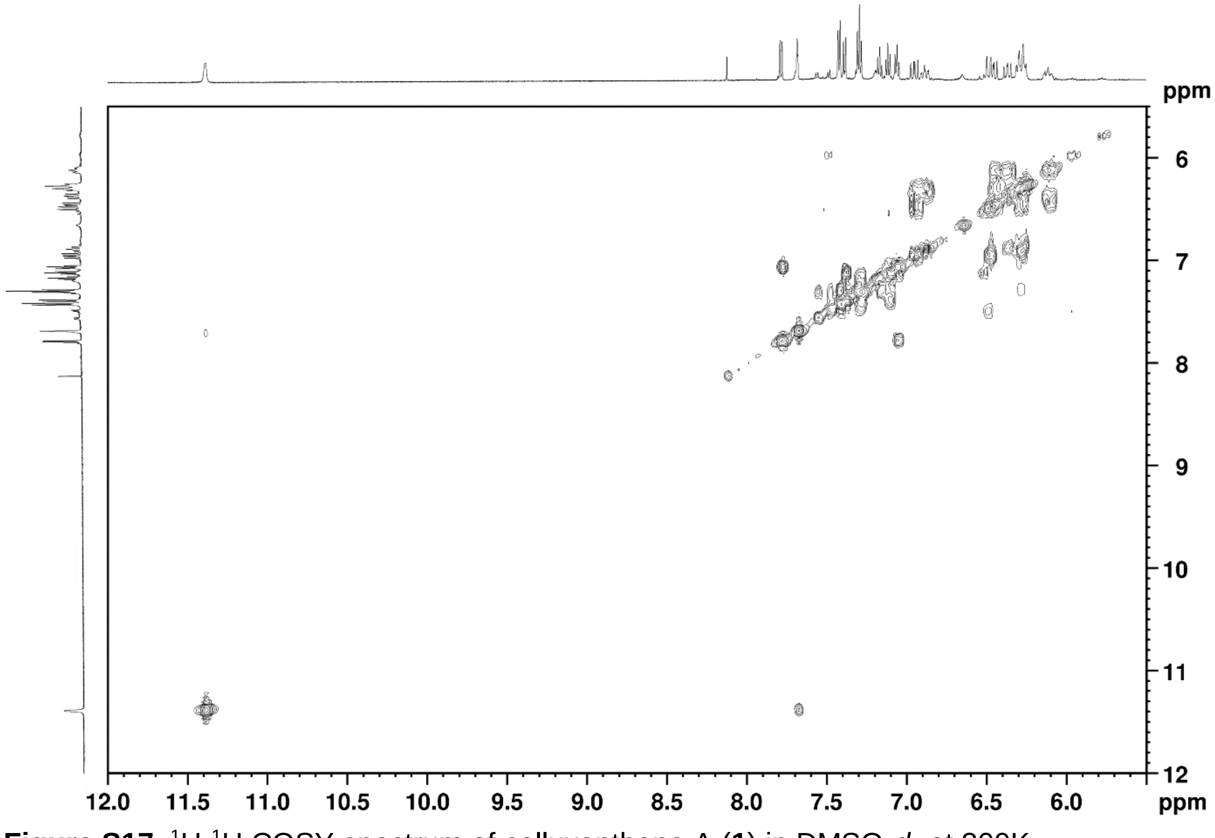

Figure S17. ^{1}H-^{1}H COSY spectrum of celluxanthene A (**1**) in DMSO-d_6 at 300K.

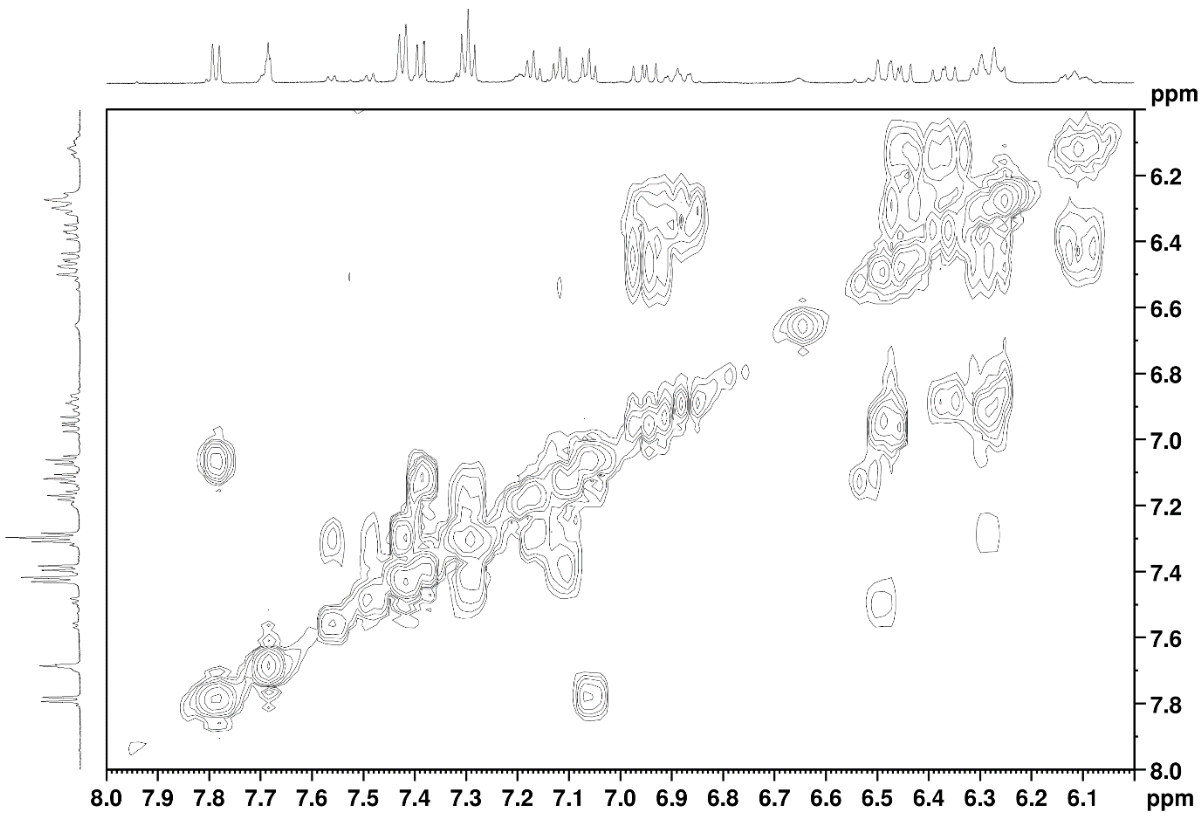

Figure S18. Extended ^{1}H-^{1}H COSY spectrum of celluxanthene A (**1**) in DMSO-d_6 at 300K.

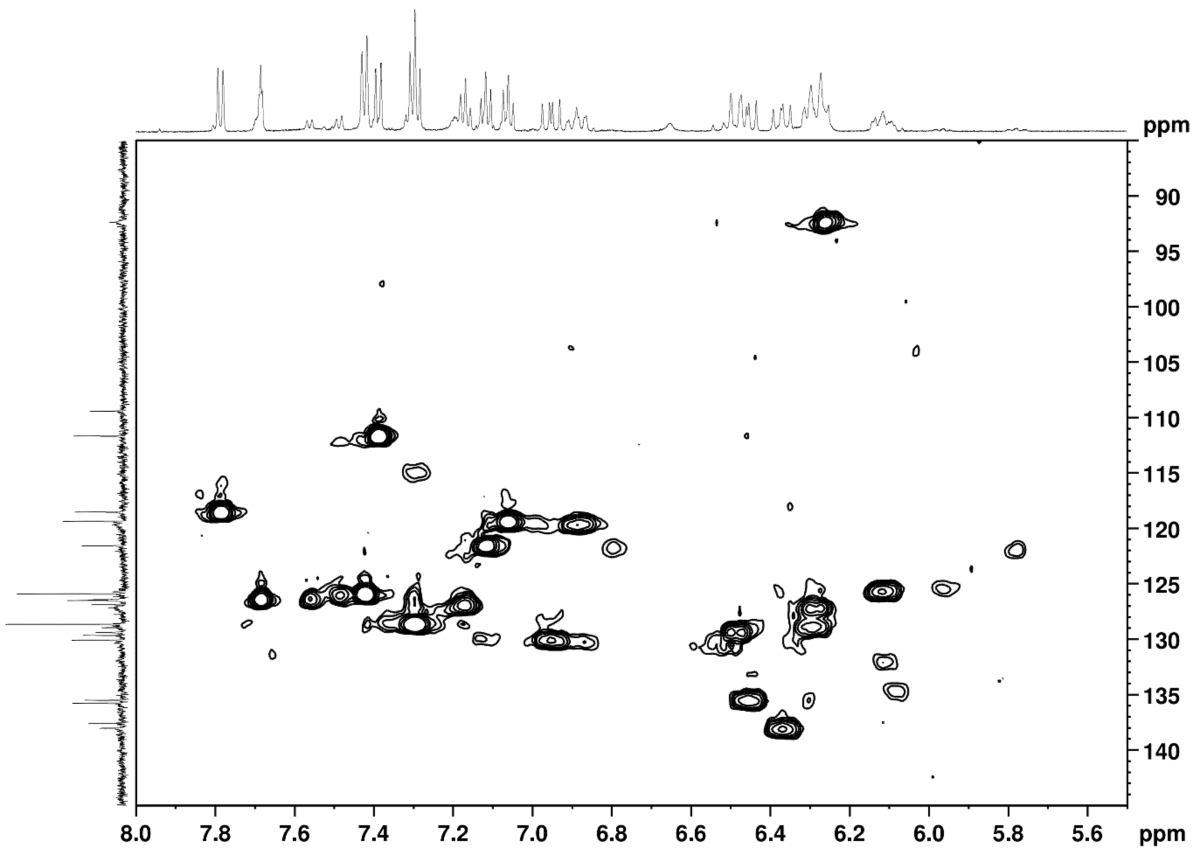

Figure S19. HSQC spectrum of celluxanthene A (**1**) in DMSO-d_6 at 300K.

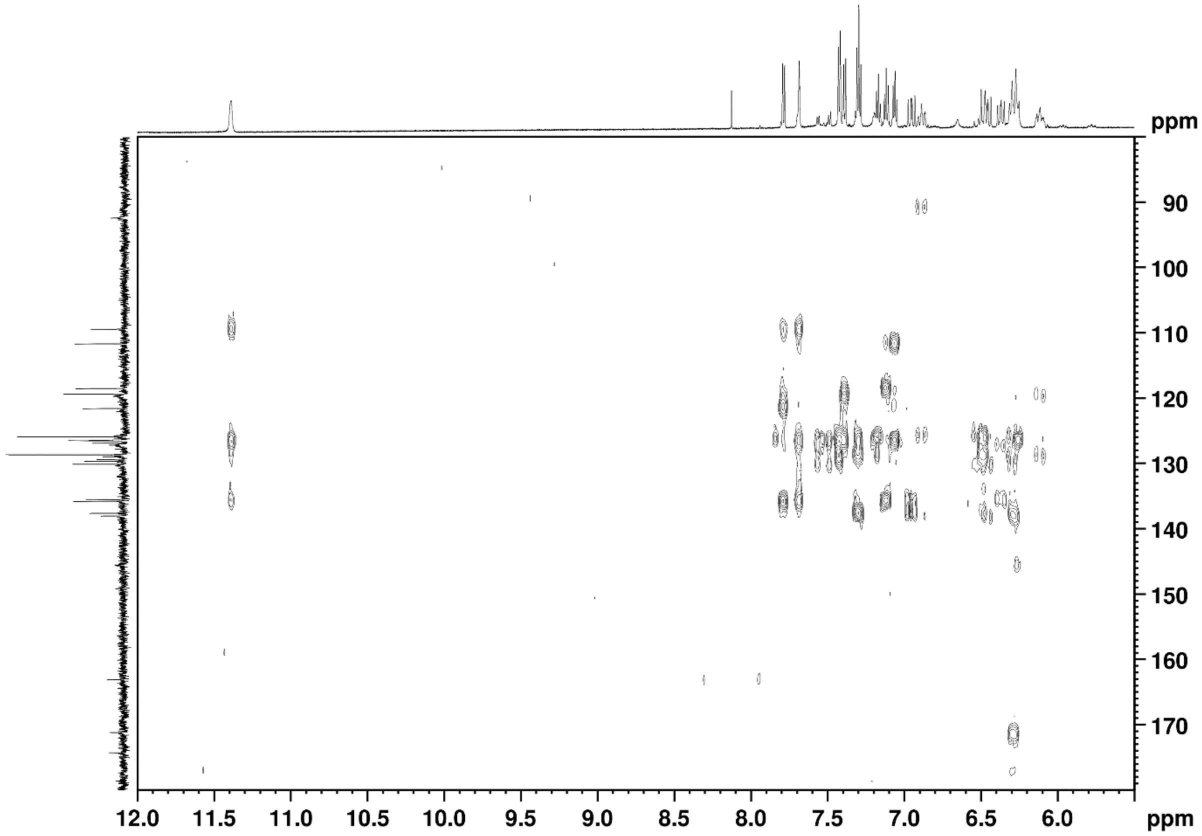

Figure S20. HMBC spectrum of celluxanthene A (**1**) in DMSO-d_6 at 300K.

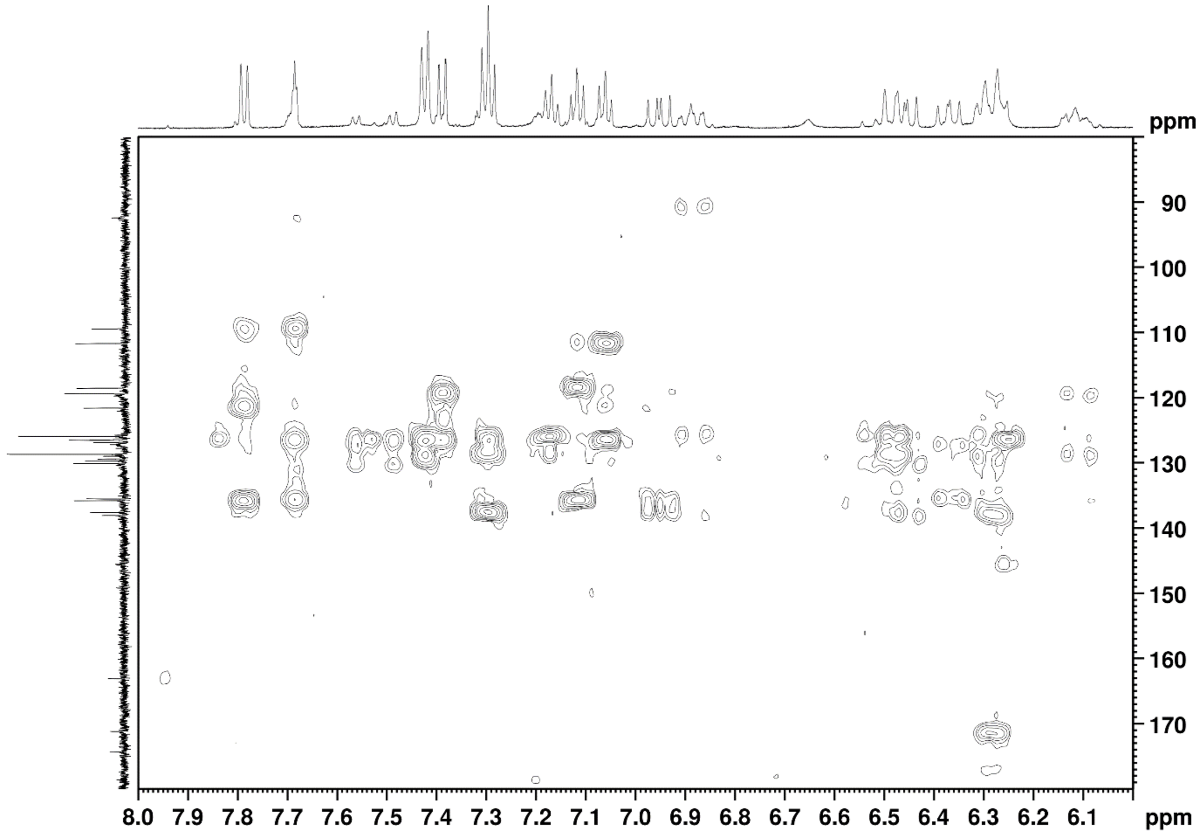

Figure S21. Extended HMBC spectrum of celluxanthene A (**1**) in DMSO-d_6 at 300K.

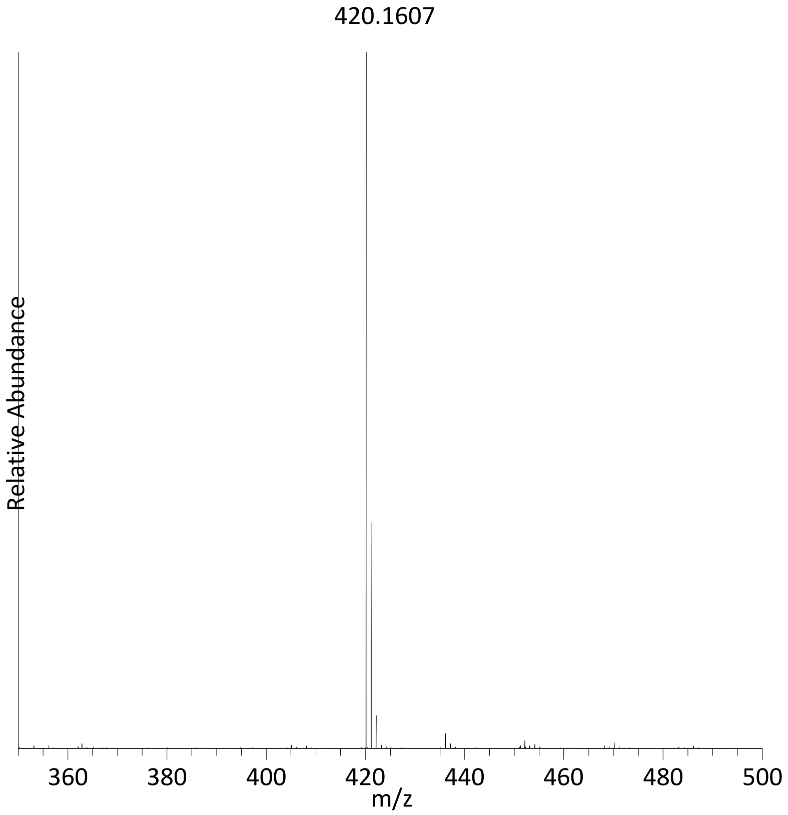

Figure S22. Negative HR-MS spectrum of 17-methoxycelluxanthene A (**6**).

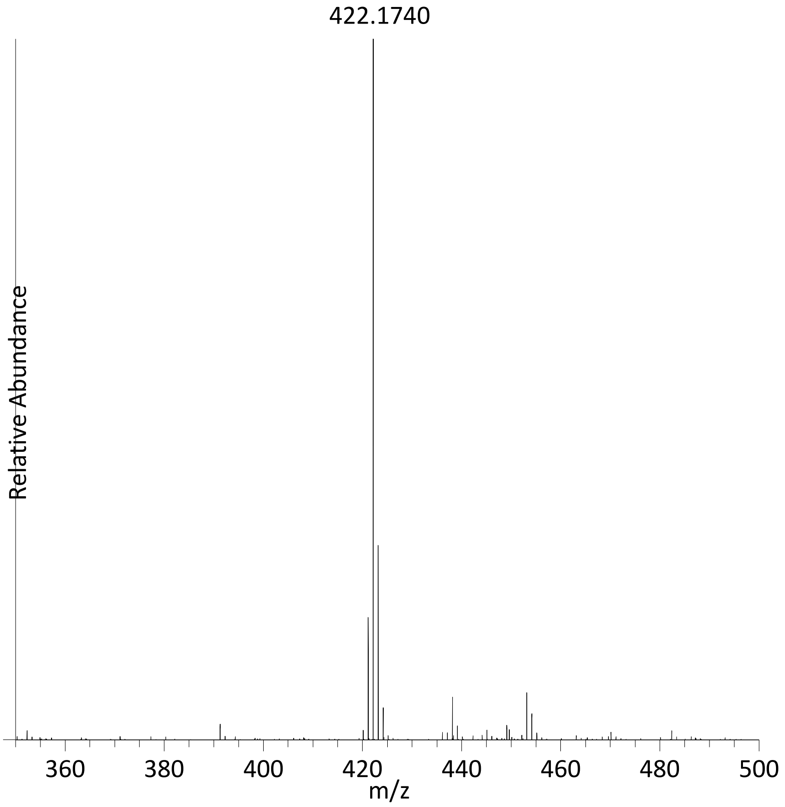

Figure S23. Positive HR-MS spectrum of 17-methoxycelluxanthene A (**6**).

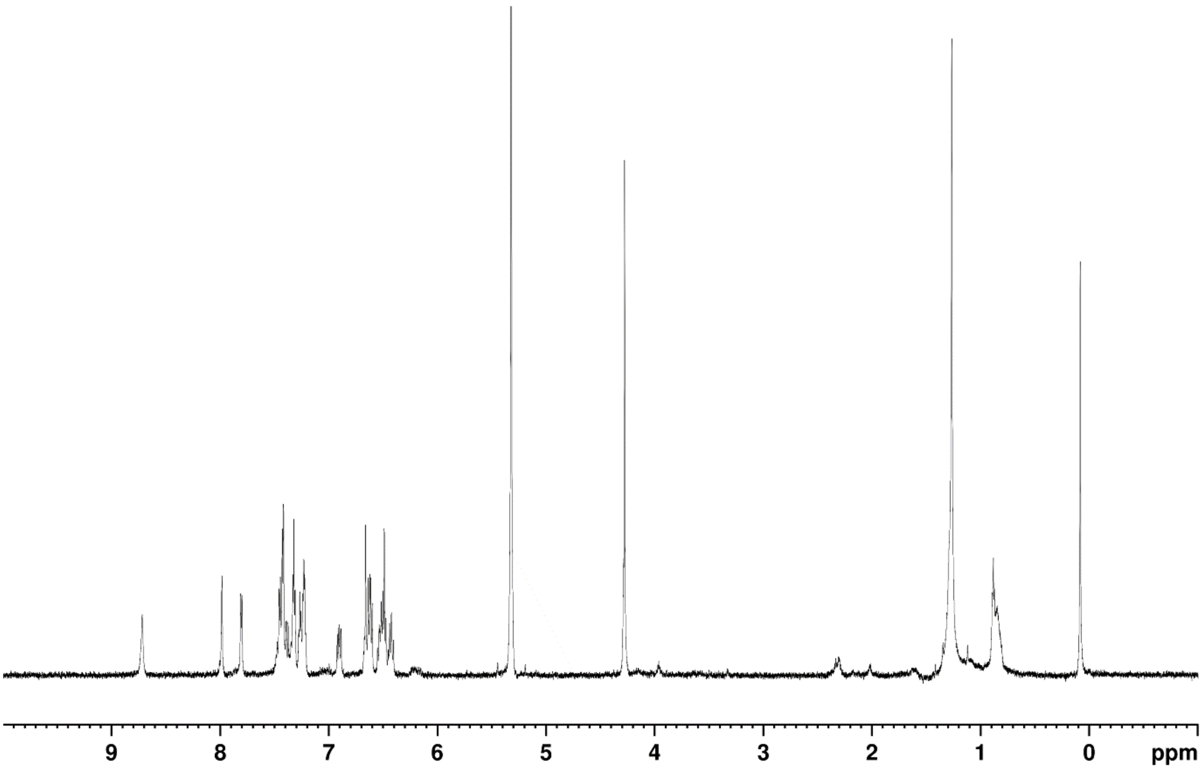

Figure S24. ^{1}H NMR spectrum of 17-methoxycelluxanthene A (**6**) in CD_2Cl_2 at 300K.

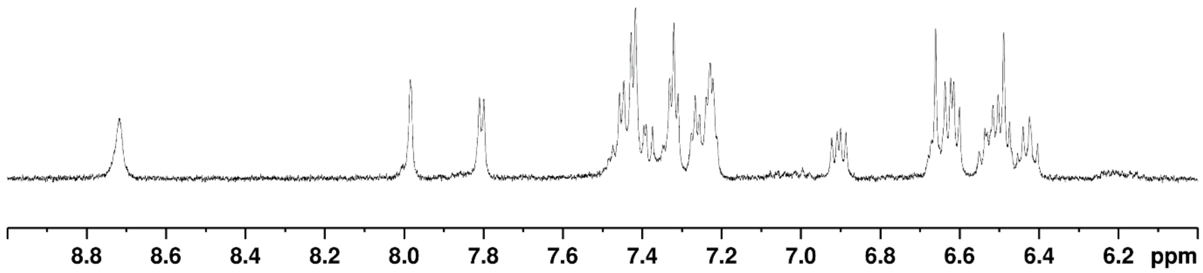

Figure S25. Extended ^{1}H NMR spectrum of 17-methoxycelluxanthene A (**6**) in CD_2Cl_2 at 300K.

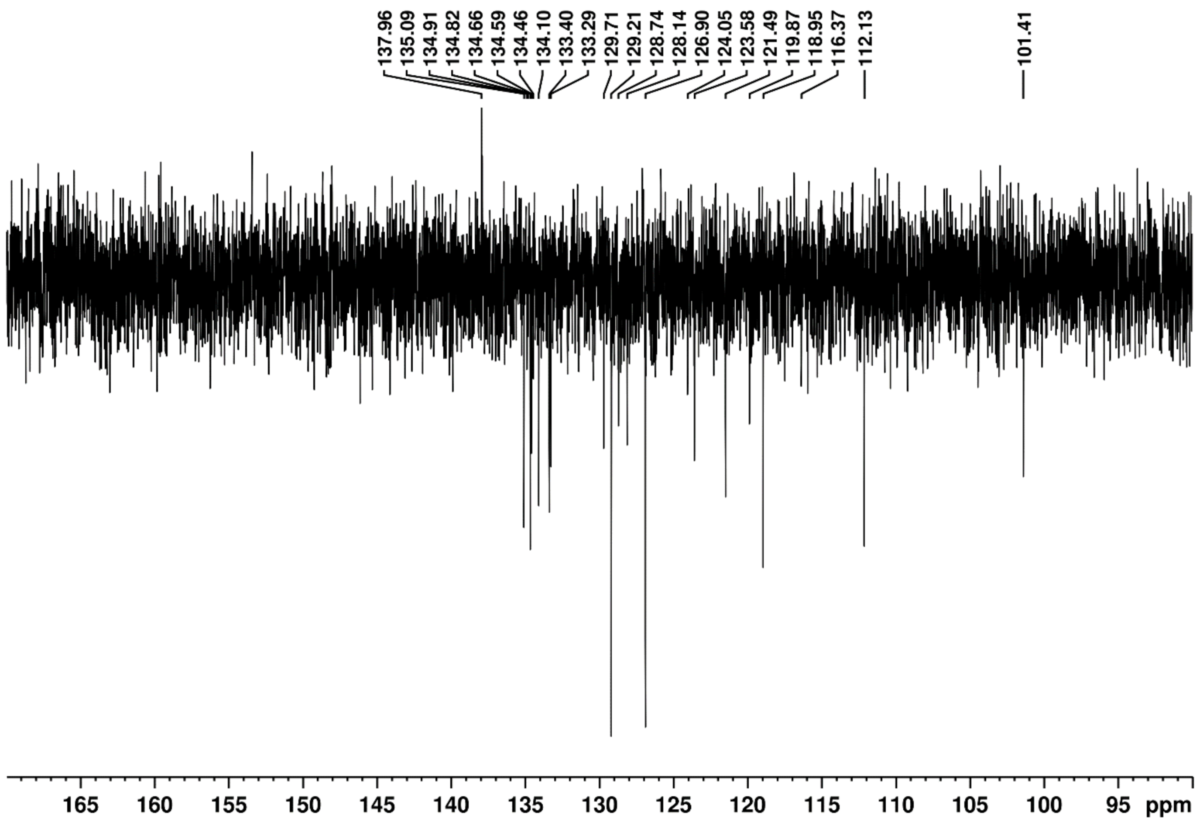

Figure S26. DEPTQ spectrum of 17-methoxycelluxanthene A (**6**) in CD_2Cl_2 at 300K.

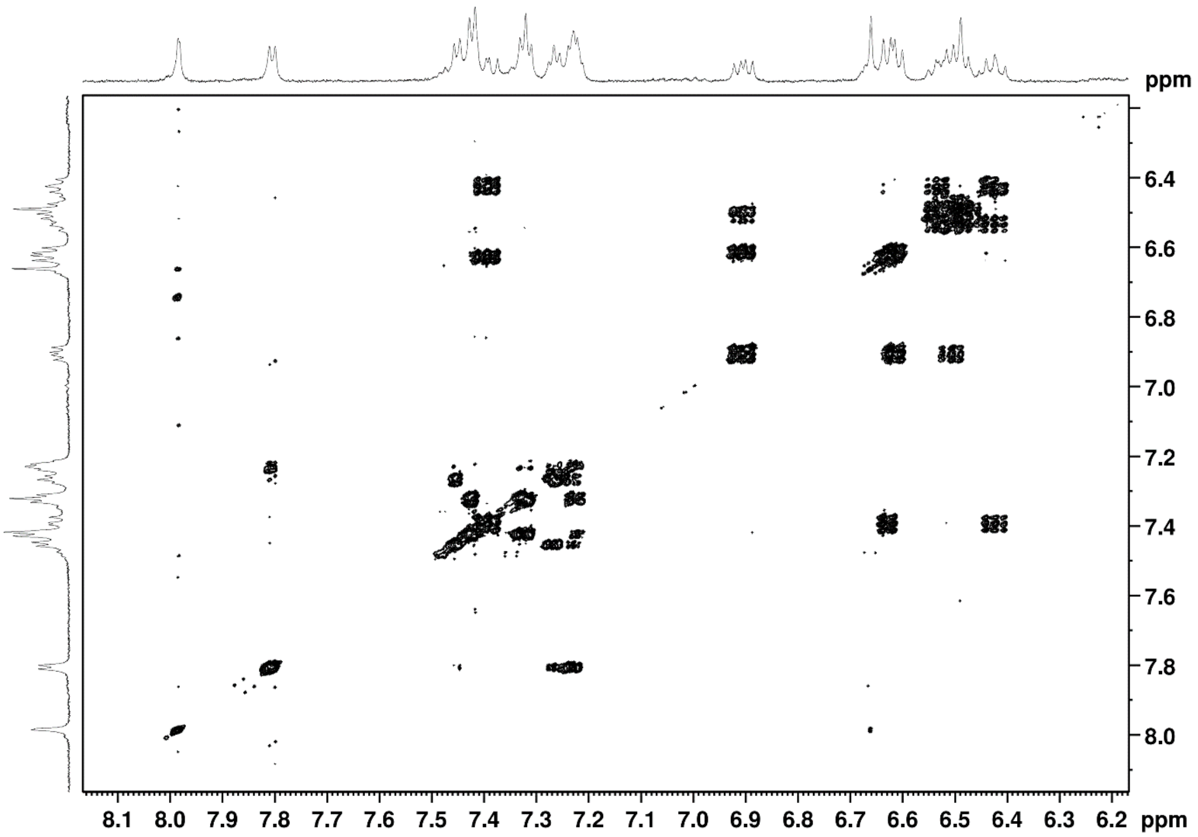

Figure S27. DQF-COSY spectrum of 17-methoxycelluxanthene A (**6**) in CD_2Cl_2 at 300K.

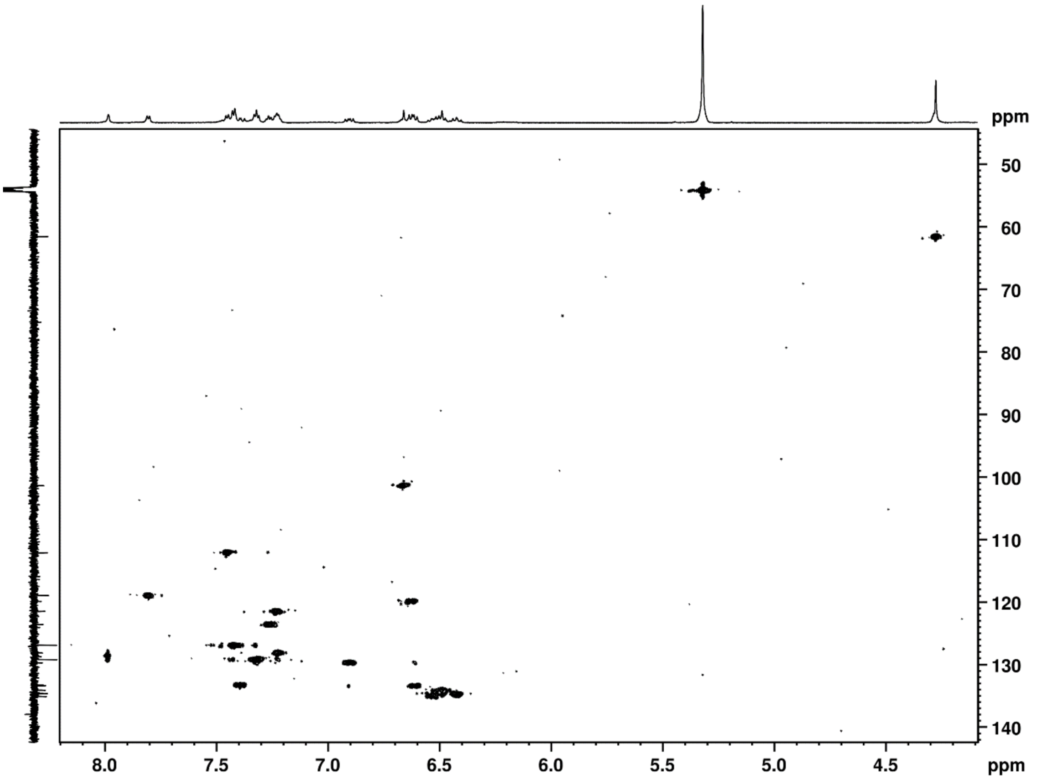

Figure S28. HSQC spectrum of 17-methoxycelluxanthene A (**6**) in CD_2Cl_2 at 300K.

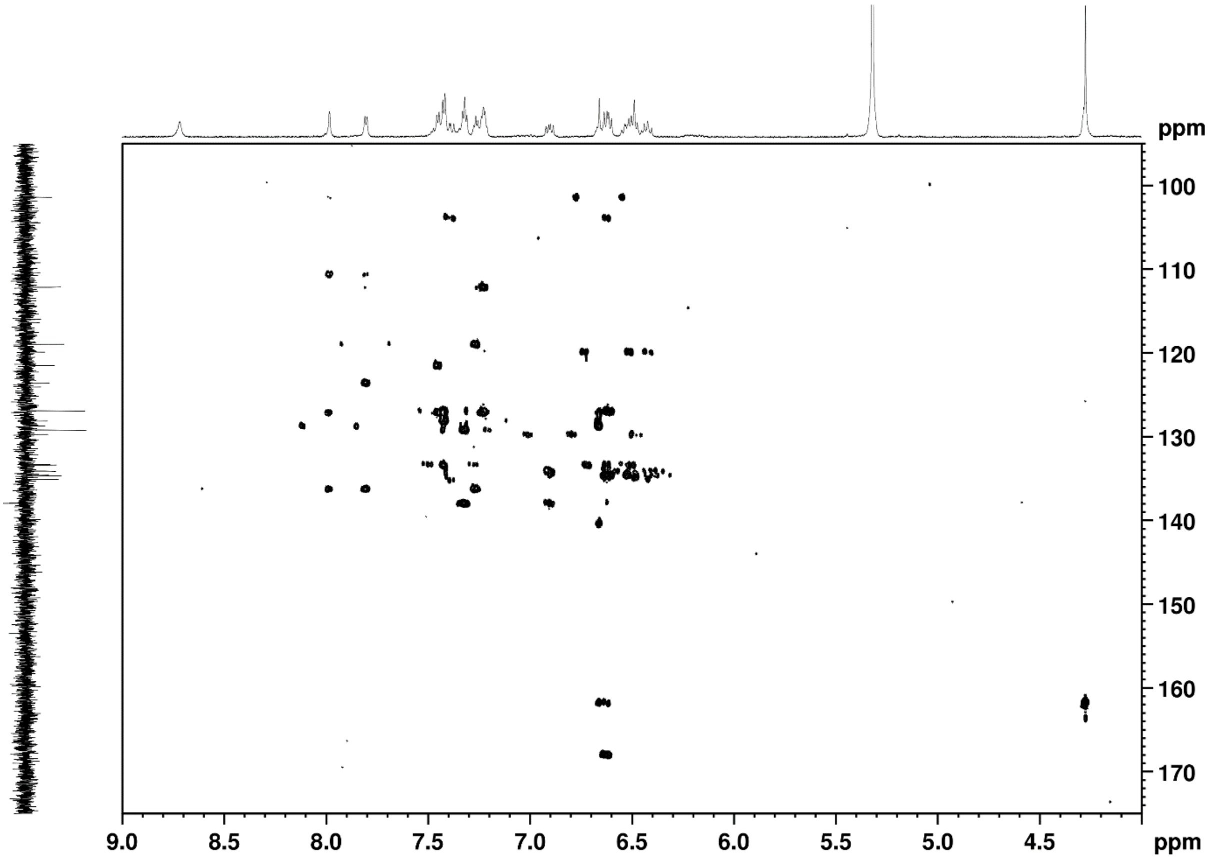

Figure S29. HMBC spectrum of 17-methoxycelluxanthene A (**6**) in CD_2Cl_2 at 300K.

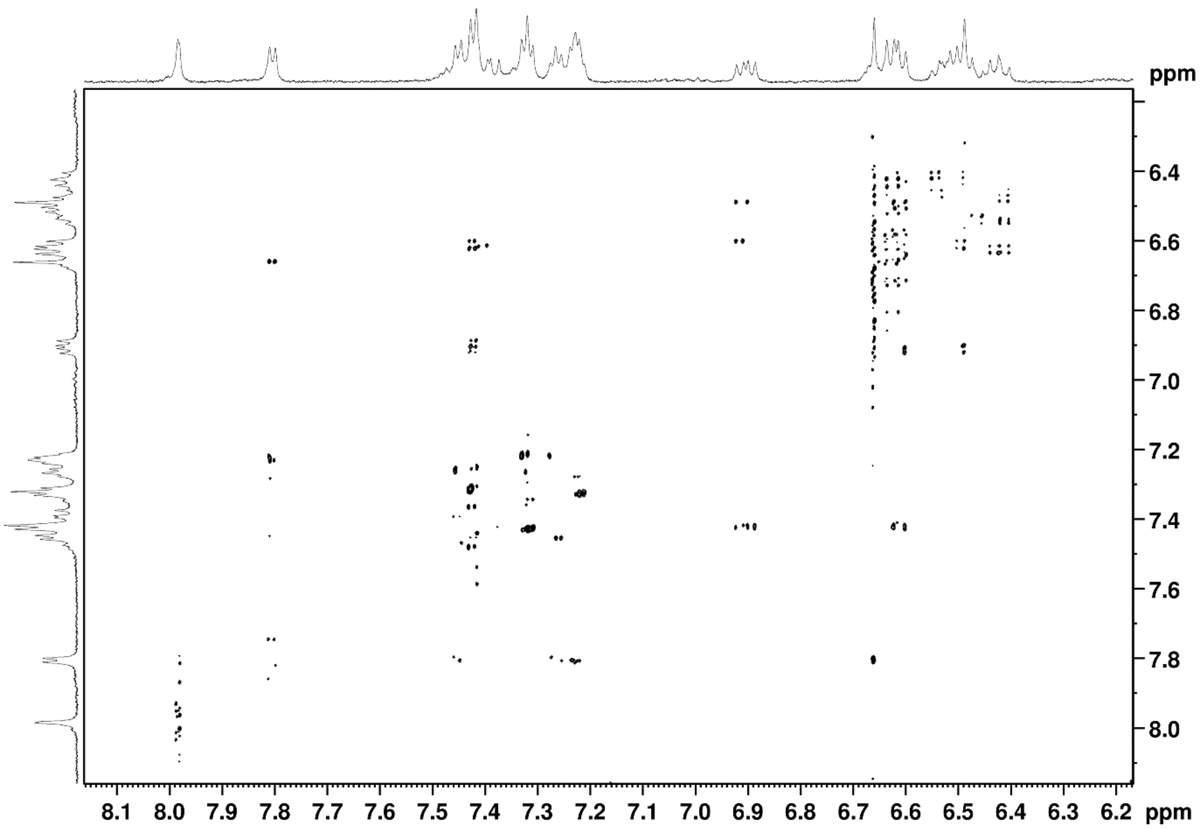

Figure S30. ROESY spectrum of 17-methoxy celluxanthene A (**6**) in CD_2Cl_2 at 300K.

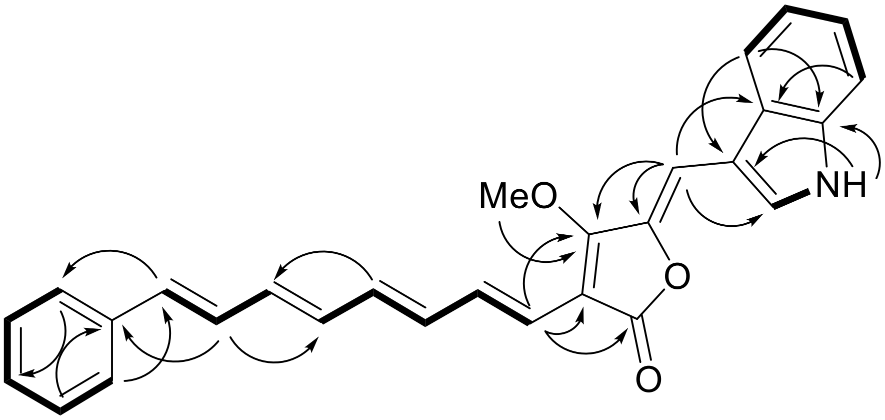

Figure S31. Selected ^1H-^1H COSY (bold lines) and HMBC (arrows) correlations of **6**.

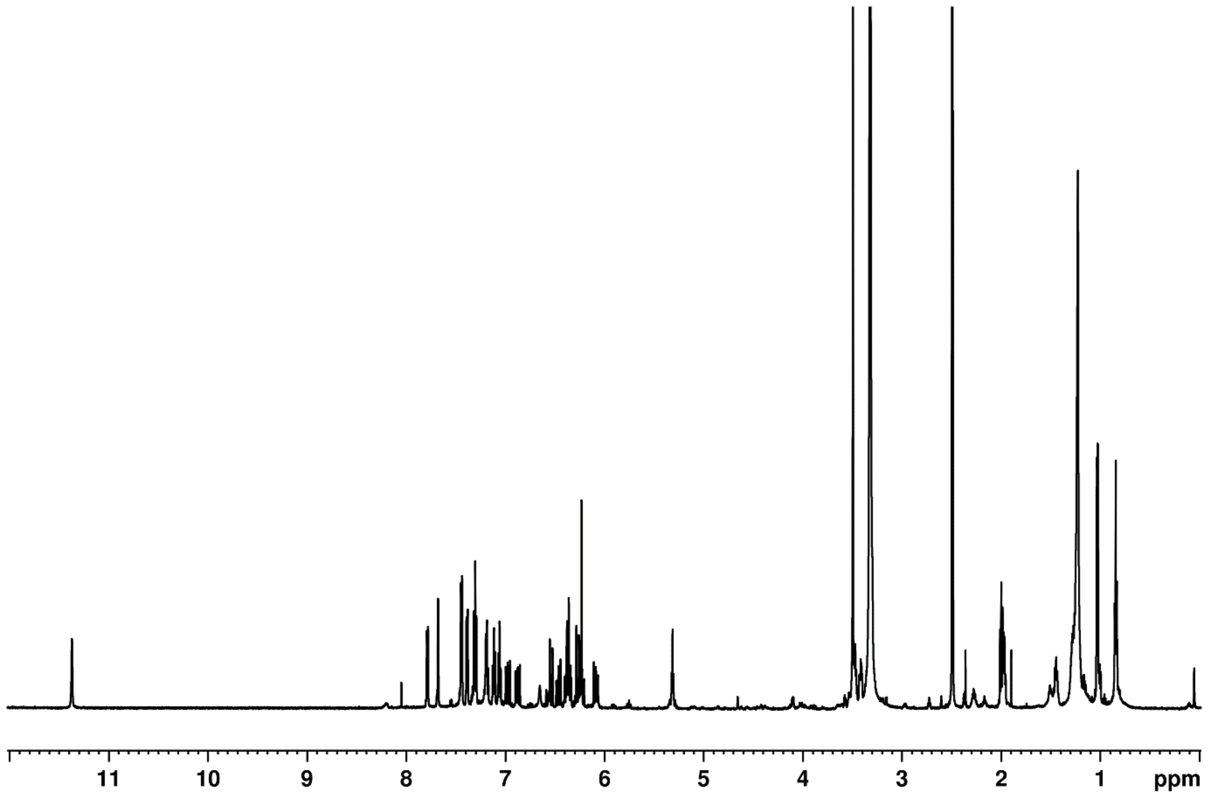

Figure S32. ^{1}H NMR spectrum of celluxanthene C (**3**) in DMSO-d_6 at 300K.

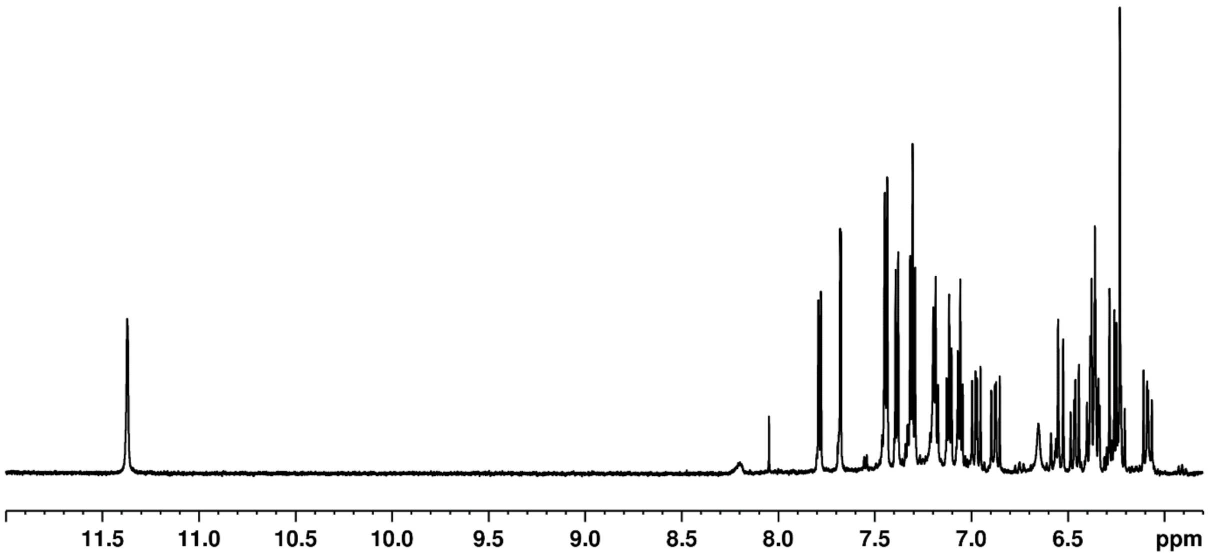

Figure S33. Extended ^{1}H NMR spectrum of celluxanthene C (**3**) in DMSO-d_6 at 300K.

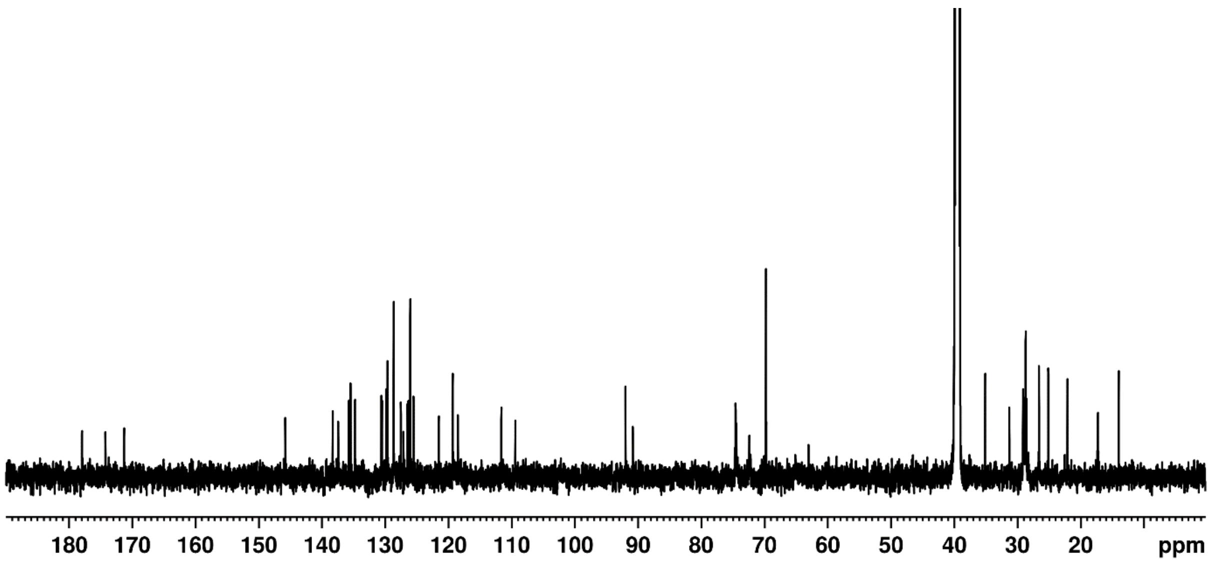

Figure S34. ^{13}C NMR spectrum of celluxanthene C (**3**) in DMSO-d_6 at 300K.

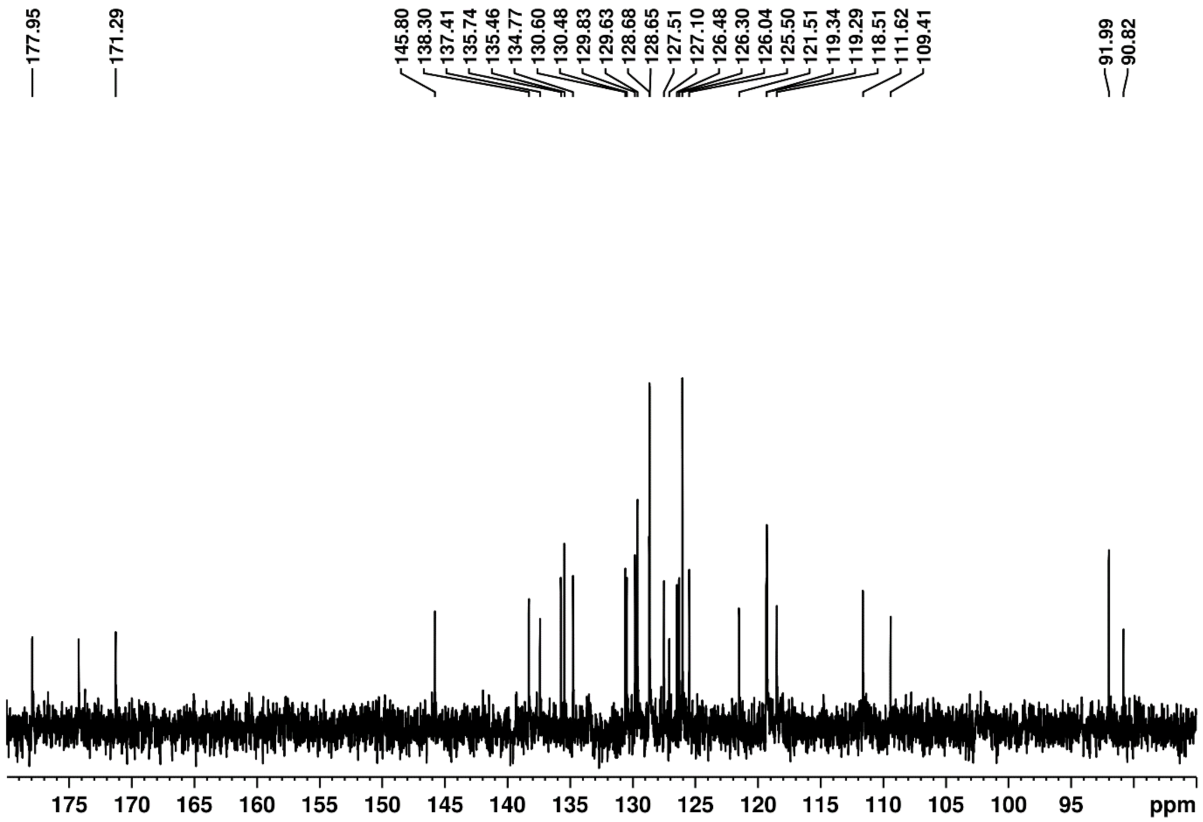

Figure S35. Extended ^{13}C NMR spectrum of celluxanthene C (**3**) in DMSO-d_6 at 300K.

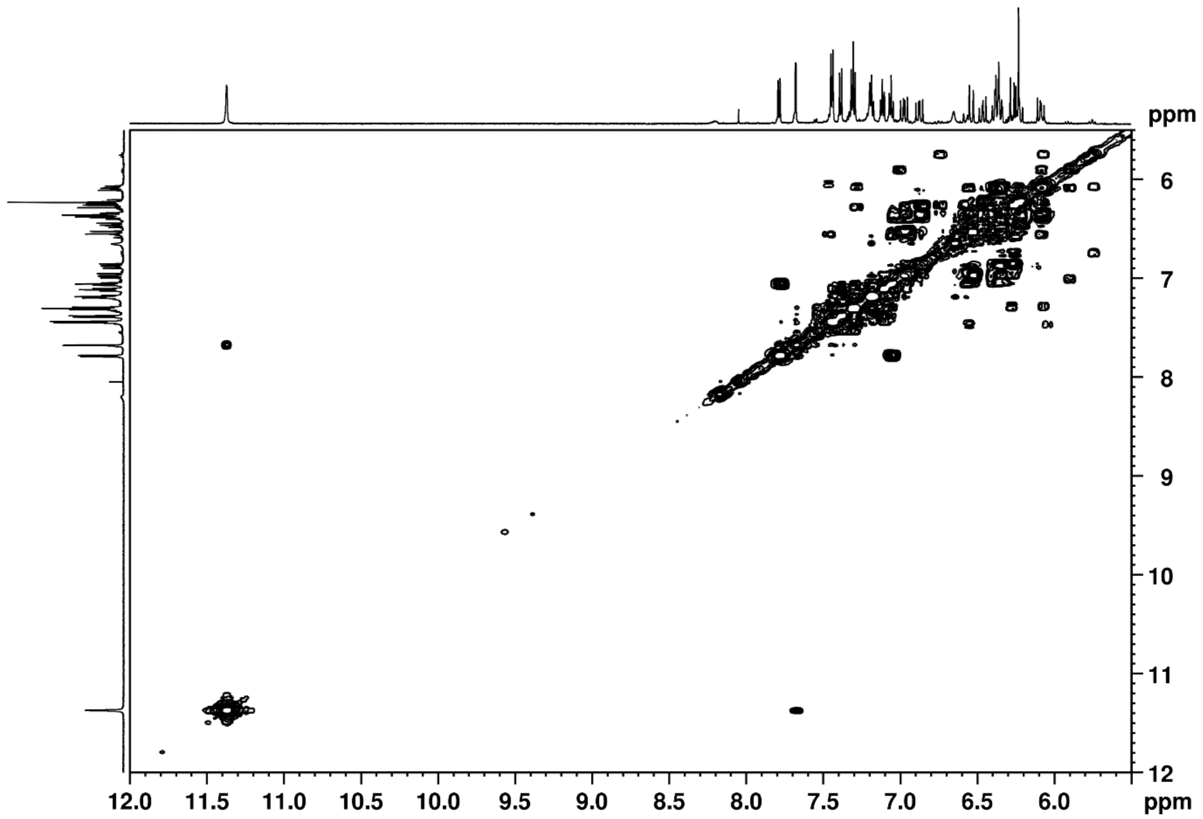

Figure S36. ^{1}H-^{1}H COSY spectrum of celluxanthene C (**3**) in DMSO-d_6 at 300K.

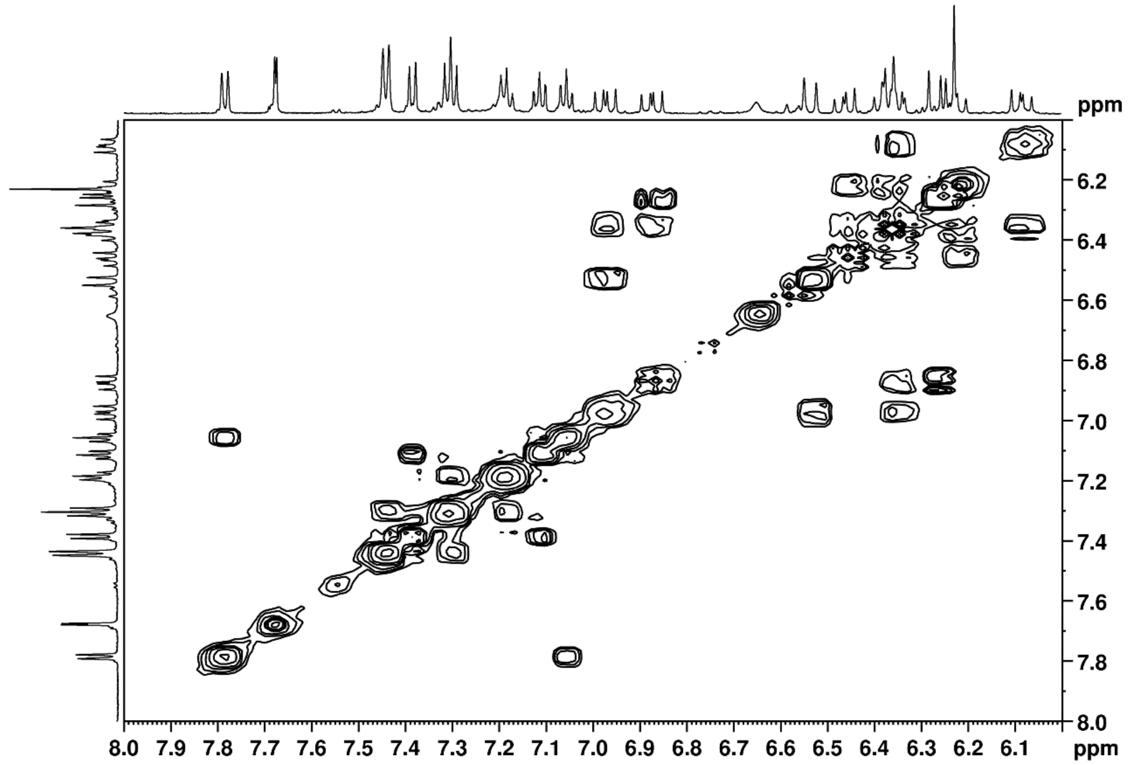

Figure S37. Extended ^{1}H-^{1}H COSY spectrum of celluxanthene C (**3**) in DMSO-d_6 at 300K.

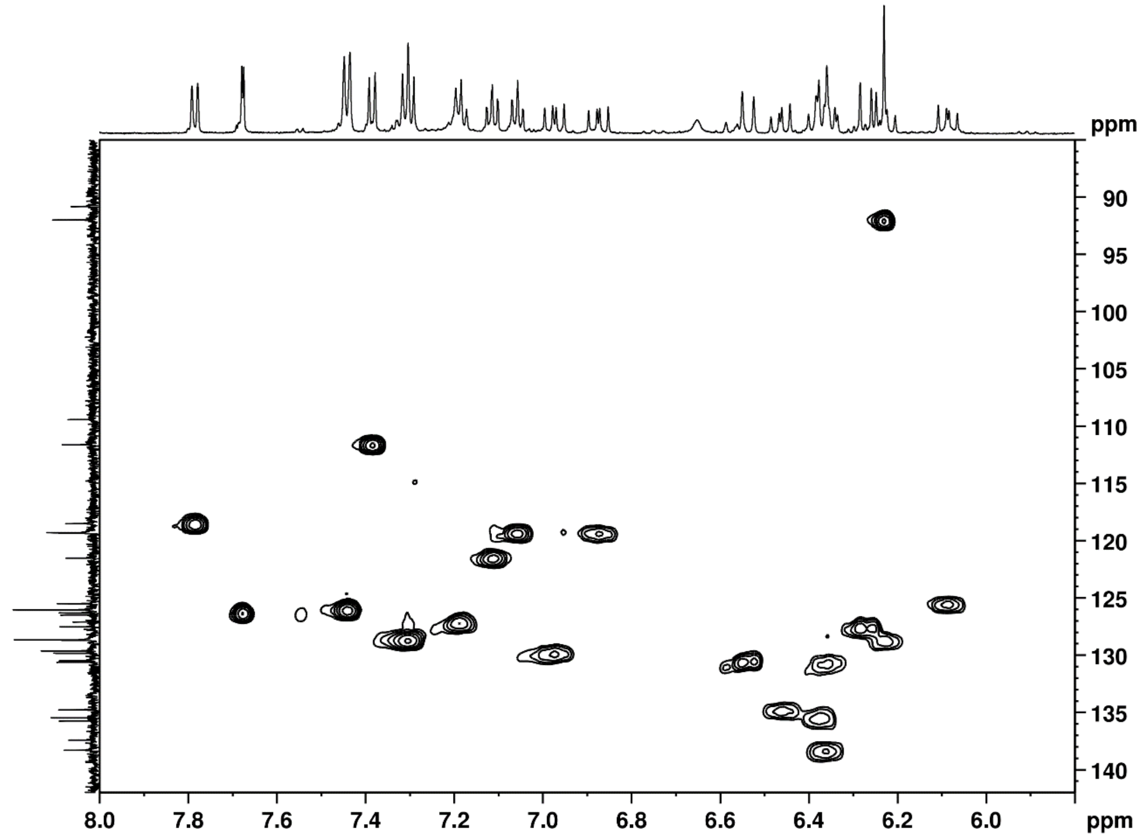

Figure S38. HSQC spectrum of celluxanthene C (**3**) in DMSO-d_6 at 300K.

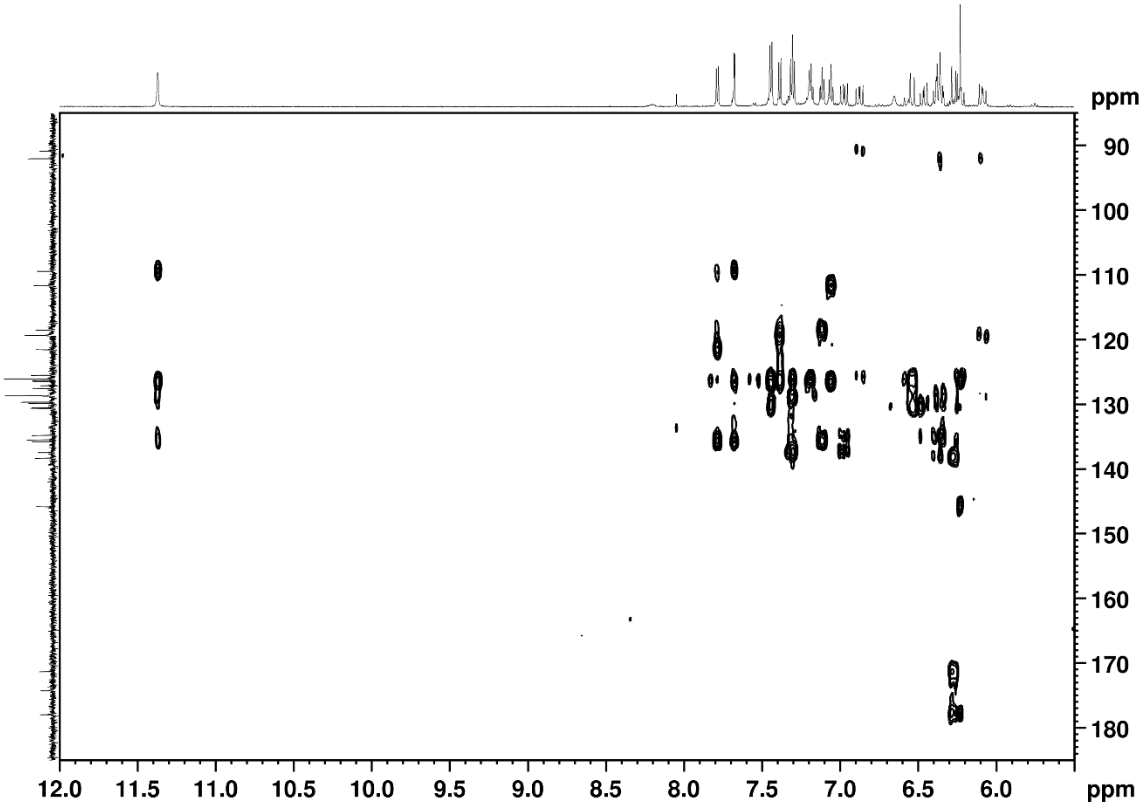

Figure S39. HMBC spectrum of celluxanthene C (**3**) in DMSO-d_6 at 300K.

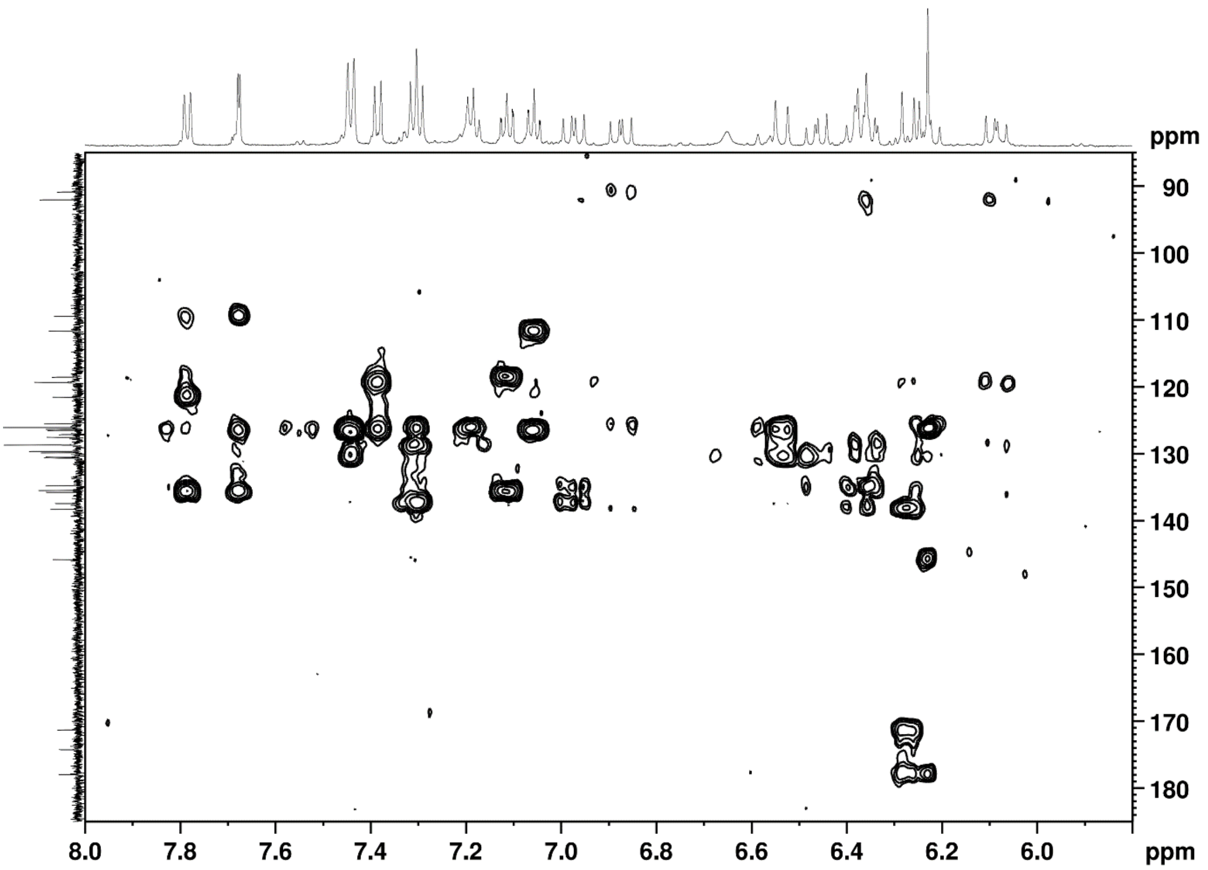

Figure S40. Extended HMBC spectrum of celluxanthene C (**3**) in DMSO-d_6 at 300K.

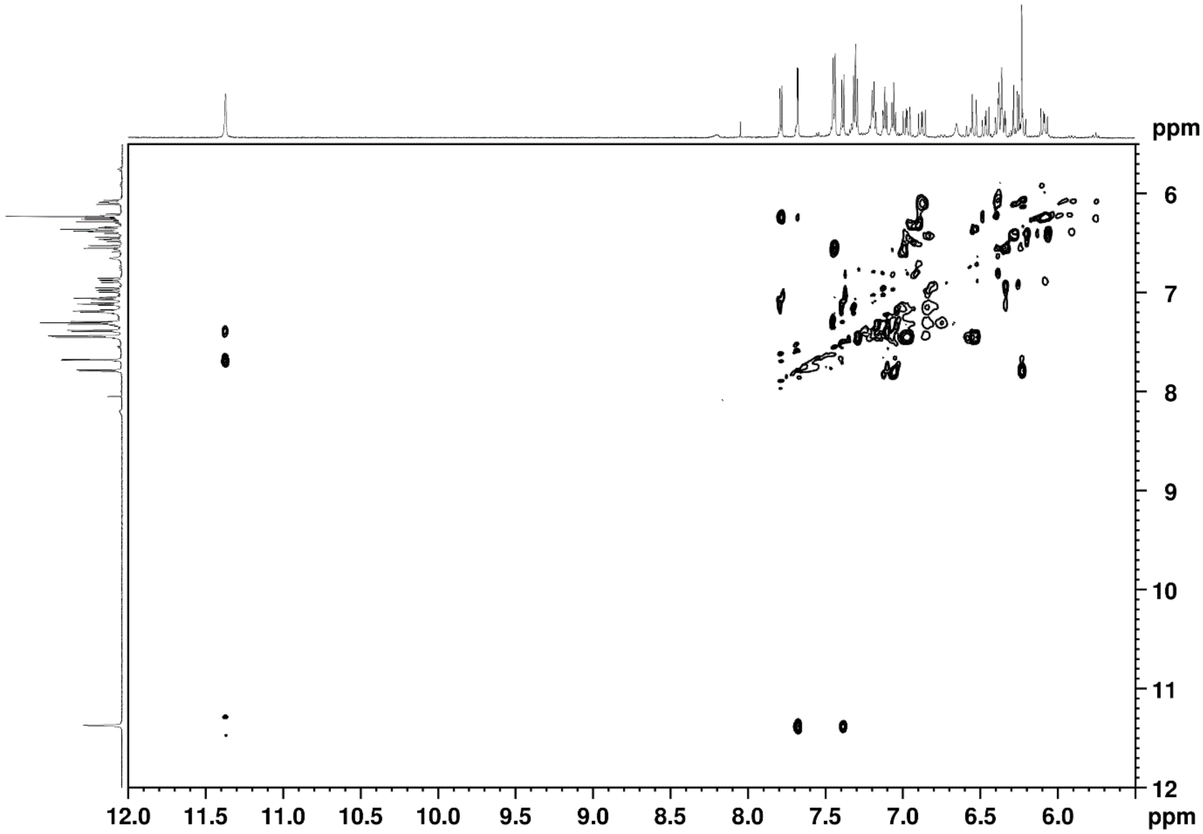

Figure S41. ROESY spectrum of celluxanthene C (**3**) in DMSO-d_6 at 300K.

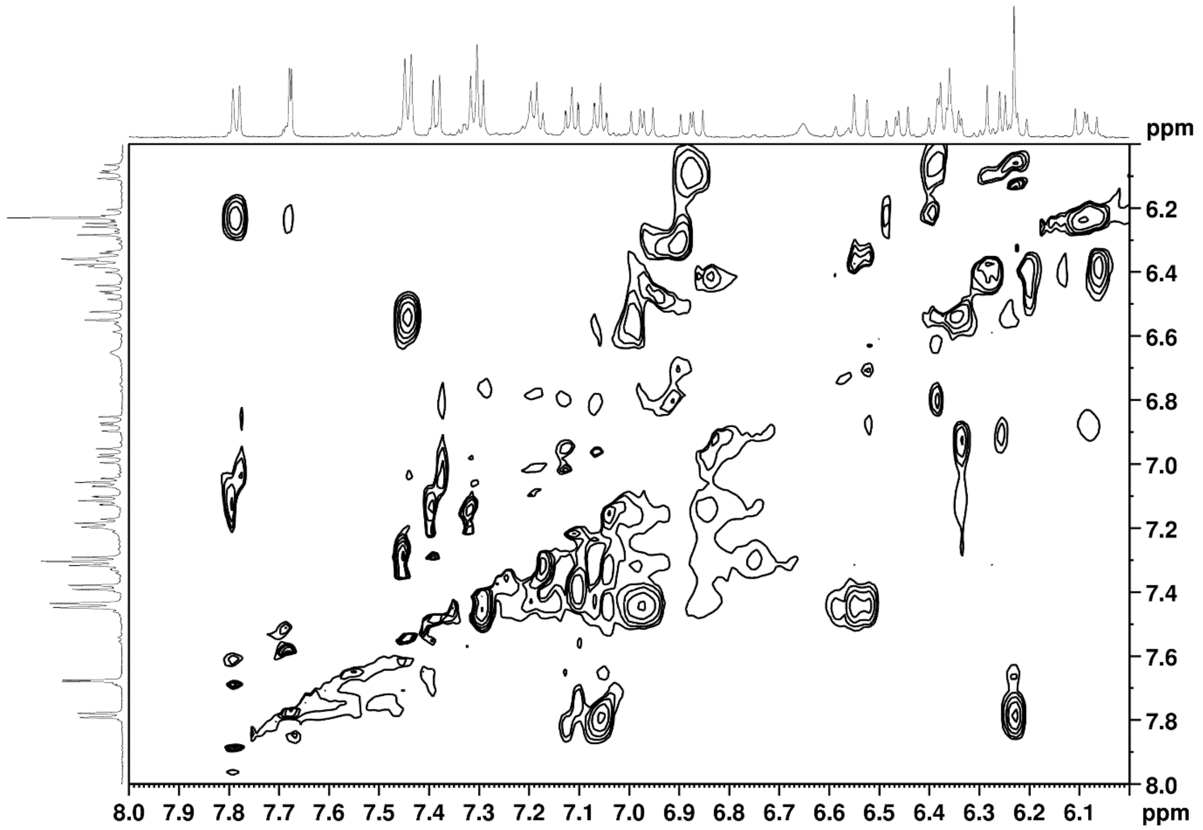

Figure S42. Extended ROESY spectrum of celluxanthene C (**3**) in DMSO-d_6 at 300K.

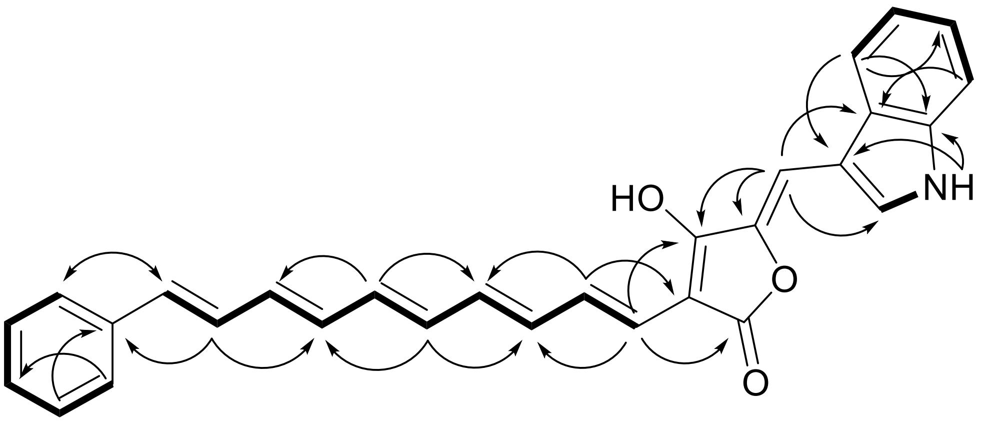

Figure S43. Selected ^{1}H-^{1}H COSY (bold lines) and HMBC (arrows) correlations of **3**.

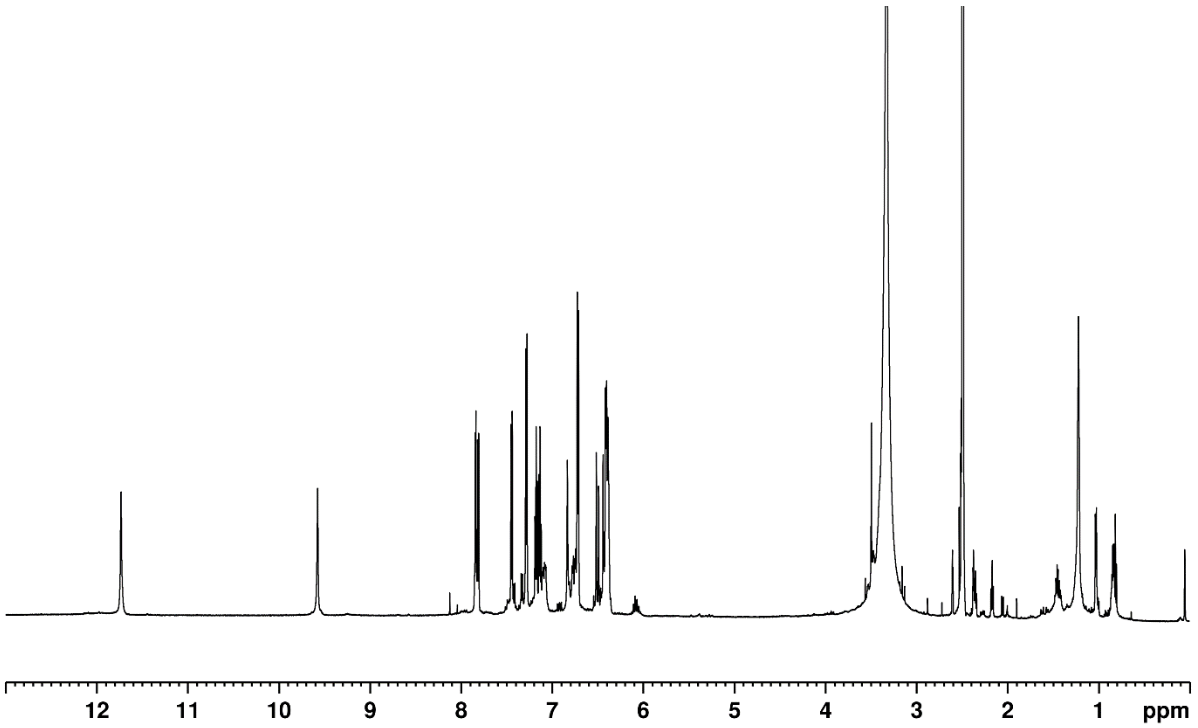

Figure S44. ^{1}H NMR spectrum of celluxanthene B (**2**) in DMSO-d_6 at 300K.

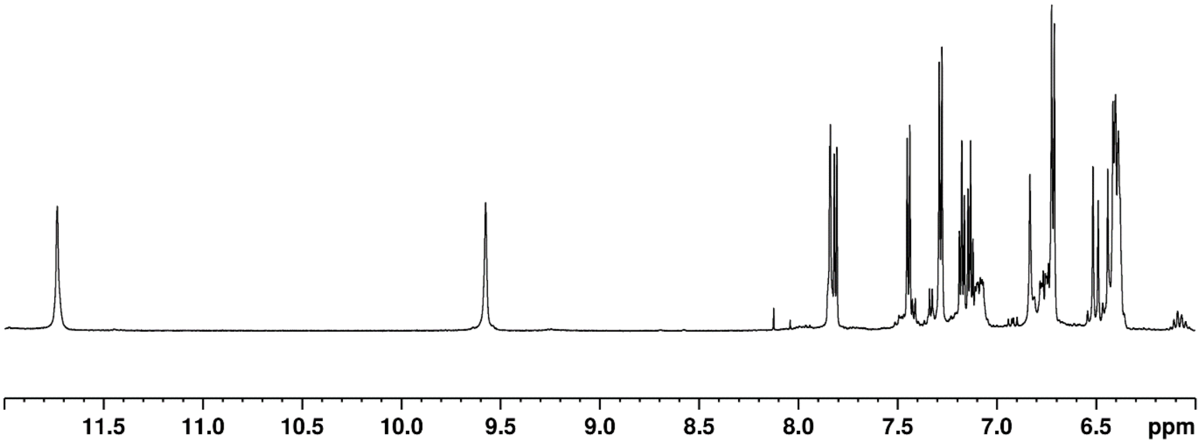

Figure S45. Extended ^{1}H NMR spectrum of celluxanthene B (**2**) in DMSO-d_6 at 300K.

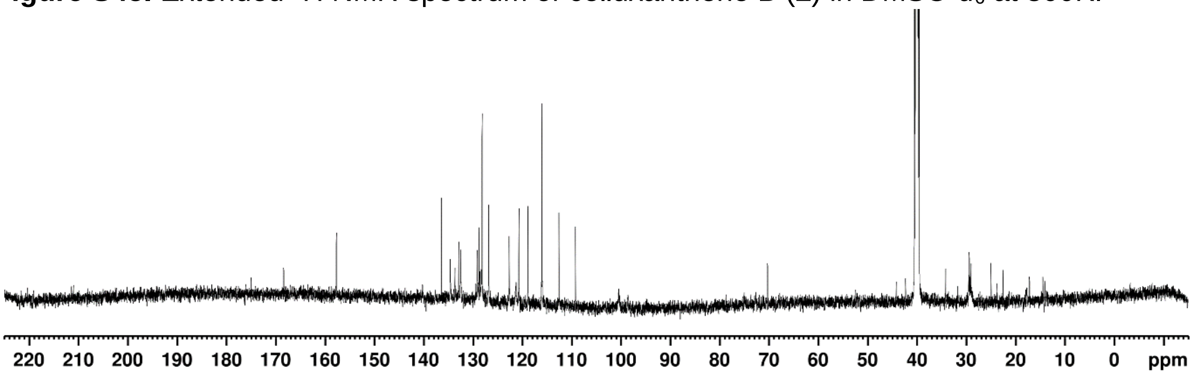

Figure S46. ^{13}C NMR spectrum of celluxanthene B (**2**) in DMSO-d_6 at 300K.

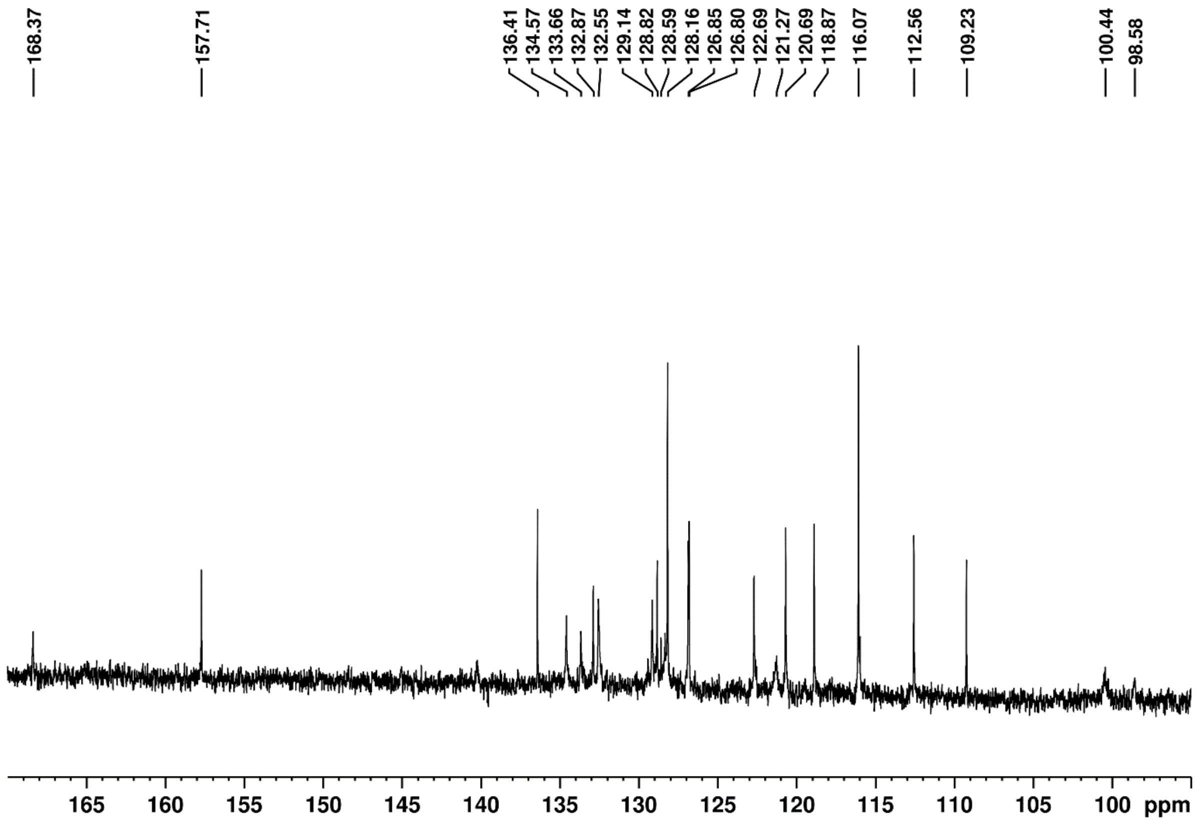

Figure S47. Extended ^{13}C NMR spectrum of celluxanthene B (**2**) in DMSO-d_6 at 300K.

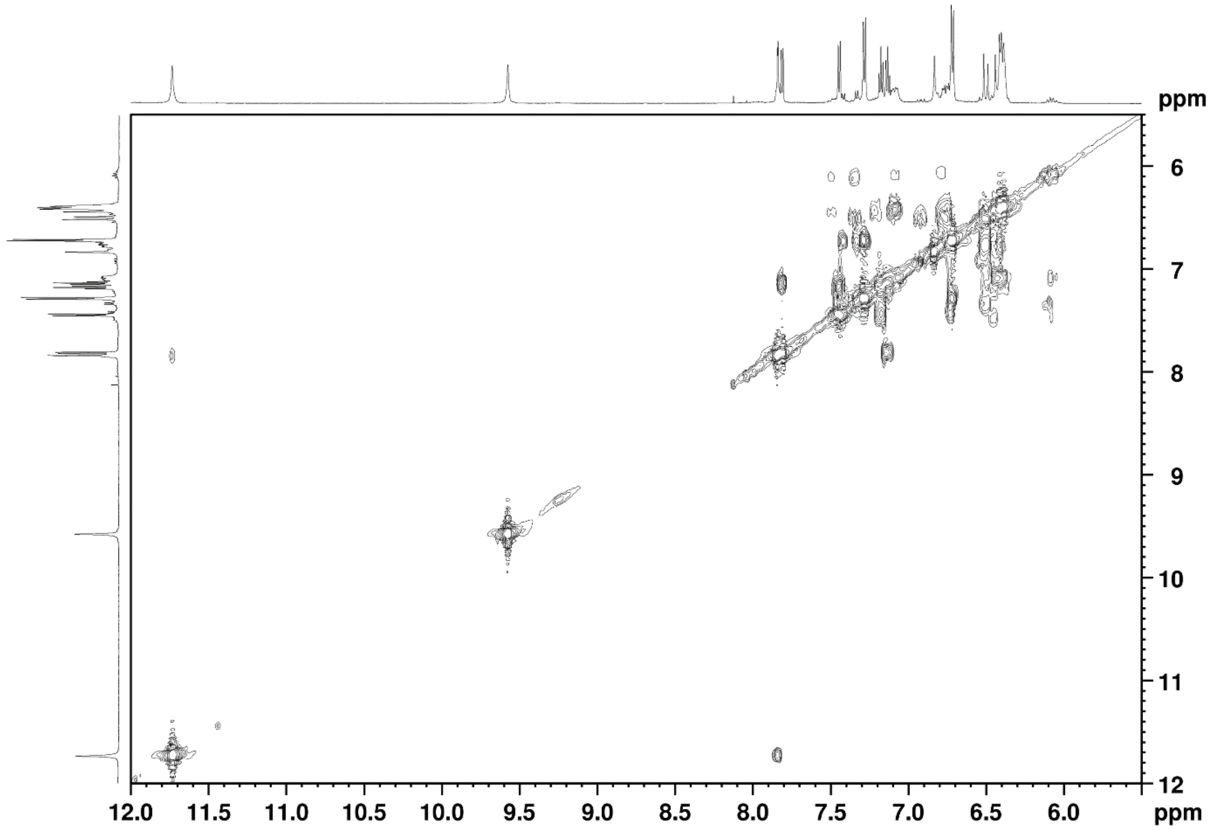

Figure S48. ^{1}H-^{1}H COSY spectrum of celluxanthene B (**2**) in DMSO-d_6 at 300K.

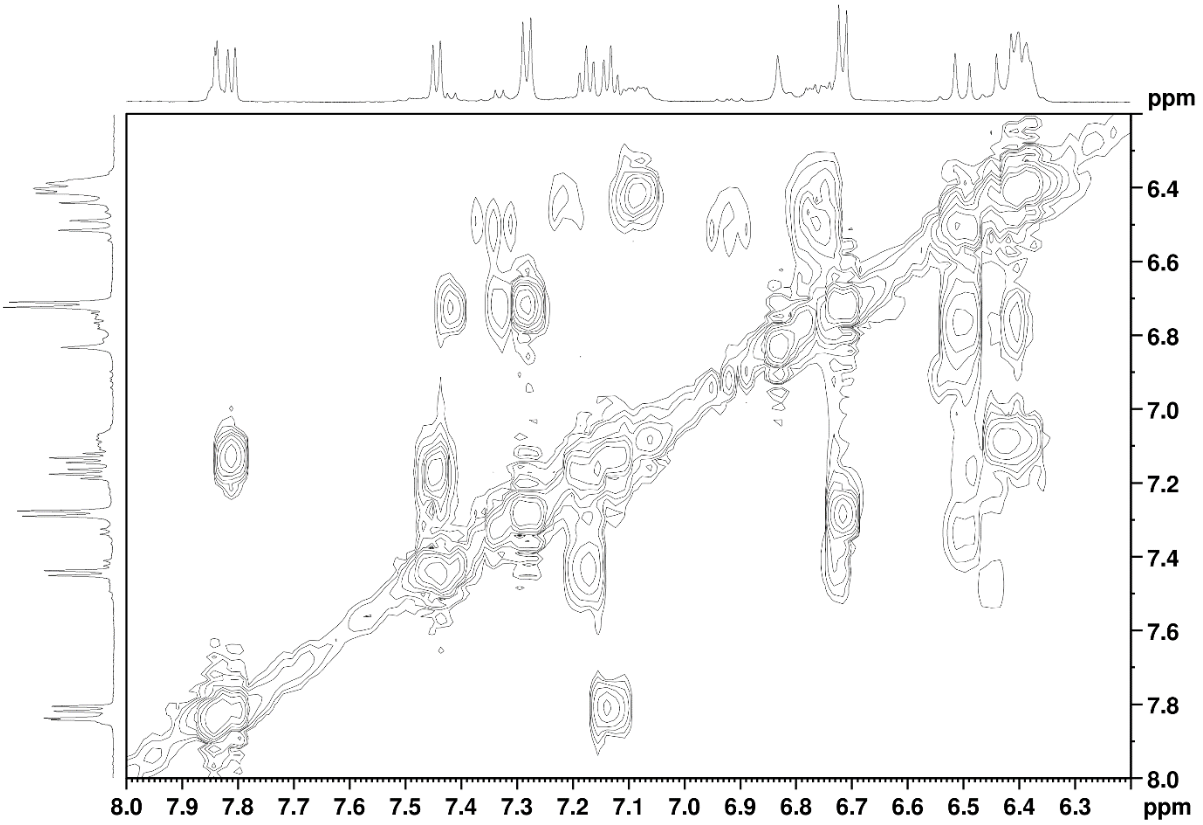

Figure S49. Extended ^{1}H-^{1}H COSY spectrum of celluxanthene B (**2**) in DMSO-d_6 at 300K.

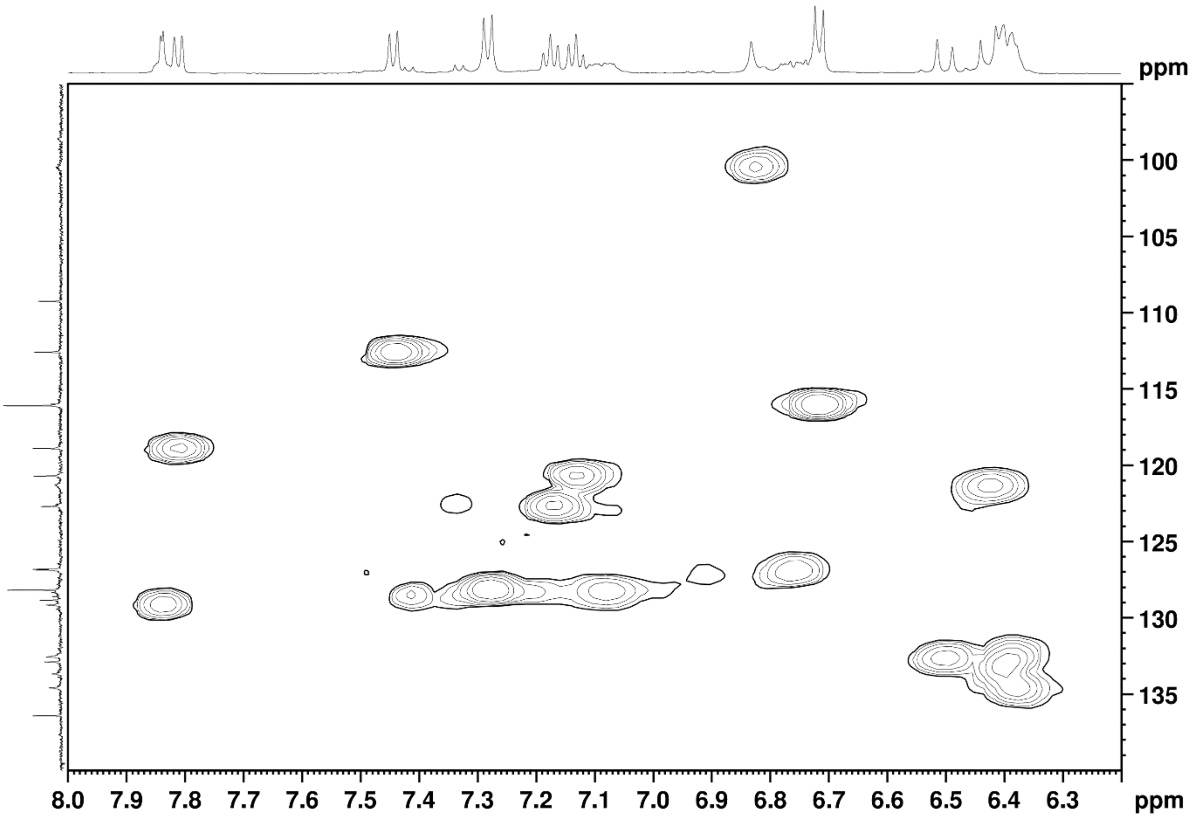

Figure S50. HSQC spectrum of celluxanthene B (**2**) in DMSO-d_6 at 300K.

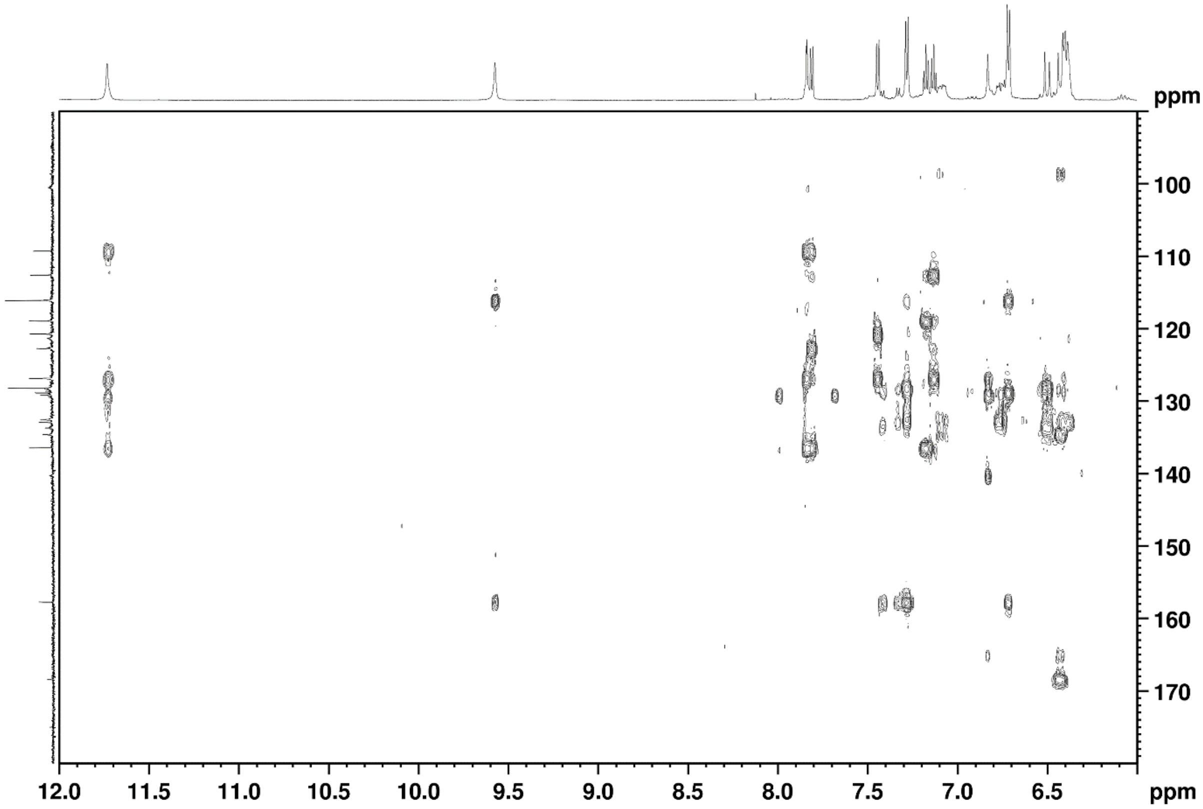

Figure S51. HMBC spectrum of celluxanthene B (**2**) in DMSO-d_6 at 300K.

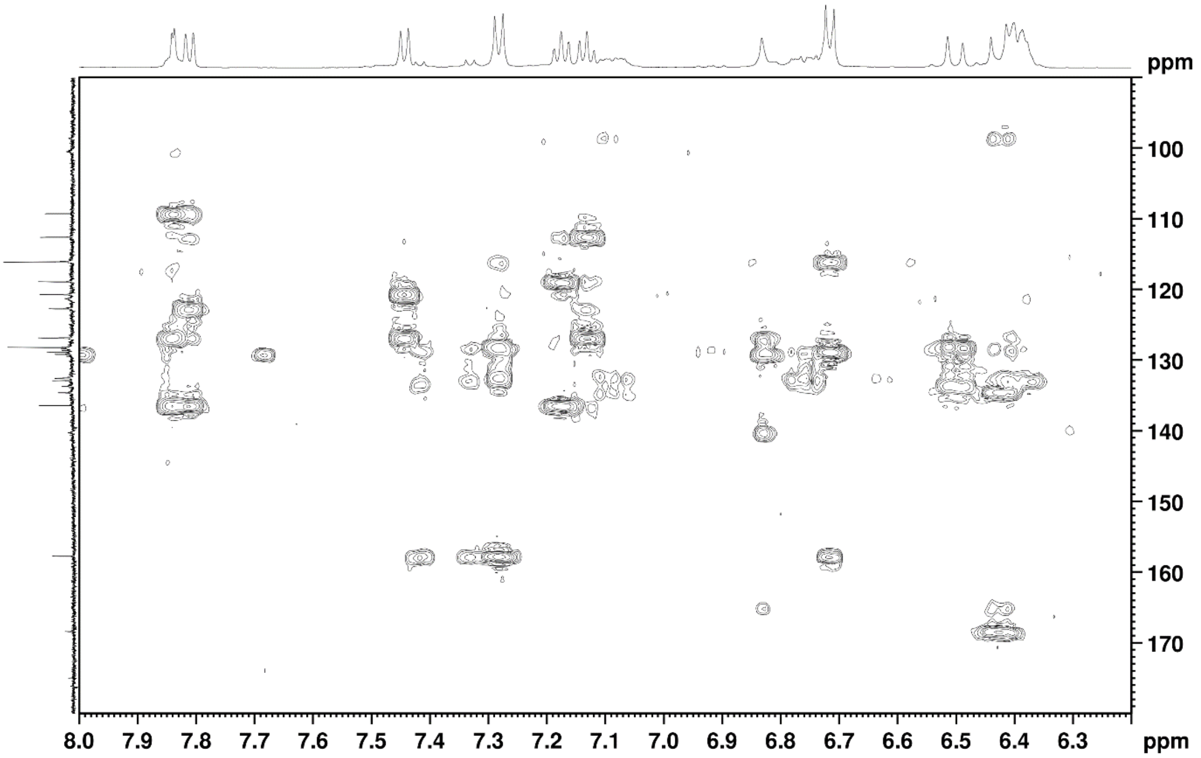

Figure S52. Extended HMBC spectrum of celluxanthene B (**2**) in DMSO-d_6 at 300K.

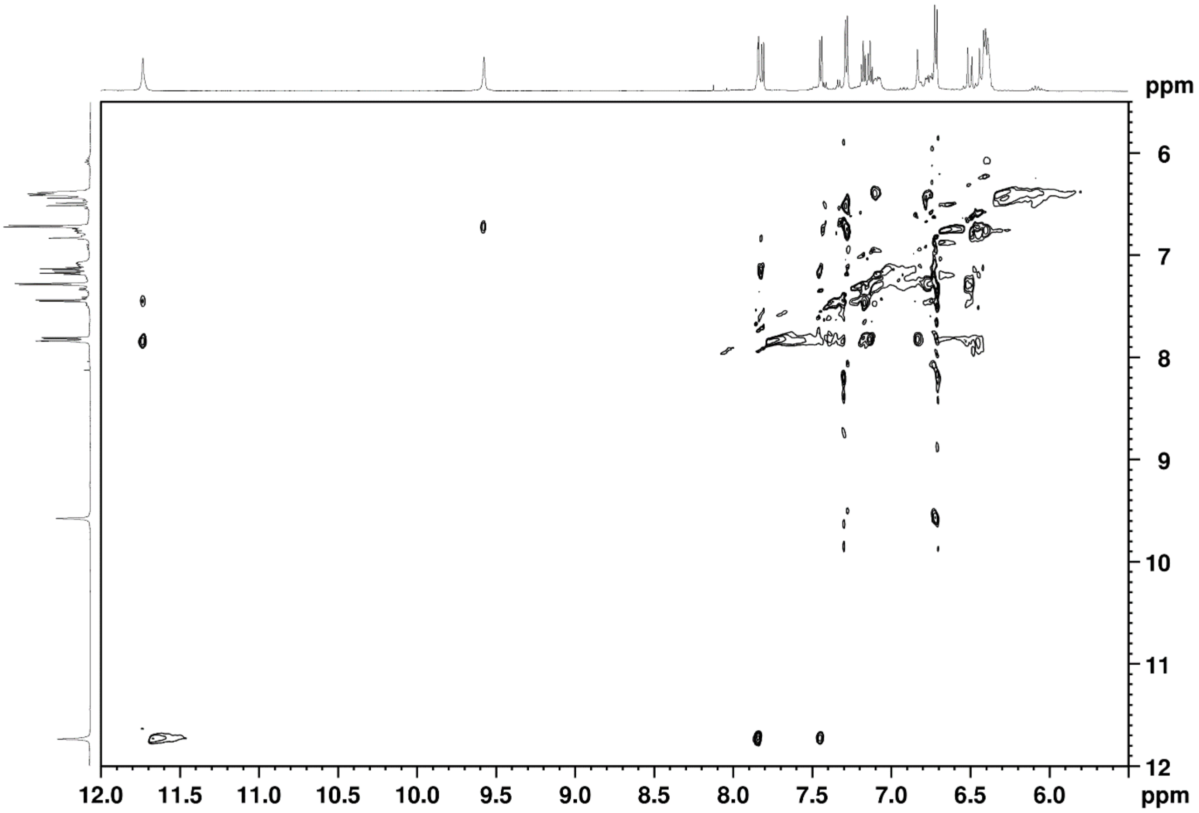

Figure S53. ROESY spectrum of celluxanthene B (**2**) in DMSO-d_6 at 300K.

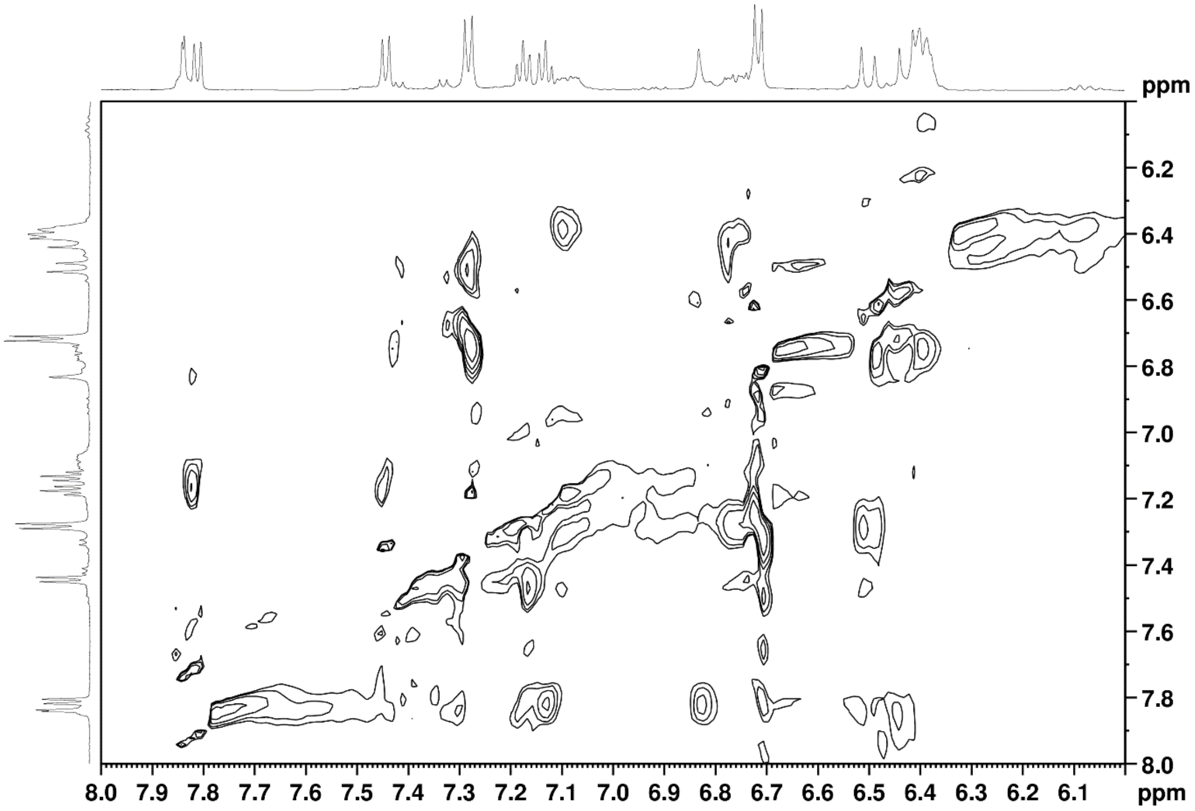

Figure S54. Extended ROESY spectrum of celluxanthene B (**2**) in DMSO-d_6 at 300K.

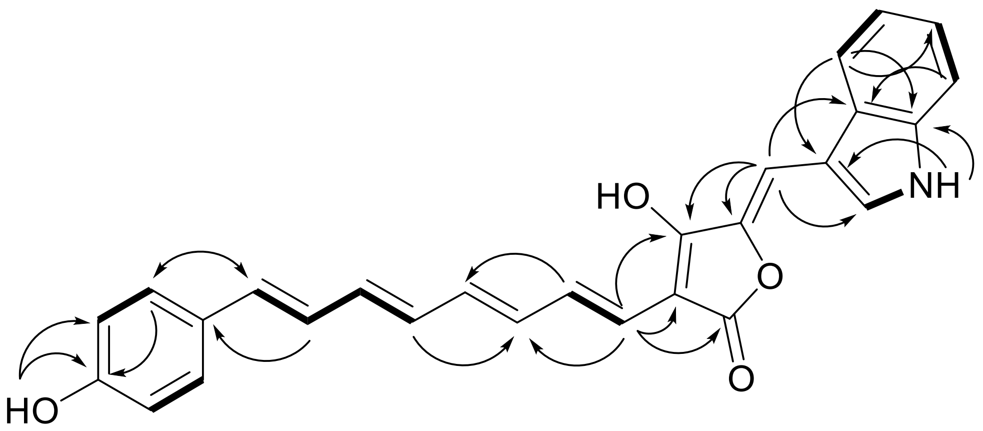

Figure S55. Selected ^{1}H-^{1}H COSY (bold lines) and HMBC (arrows) correlations of **2**.

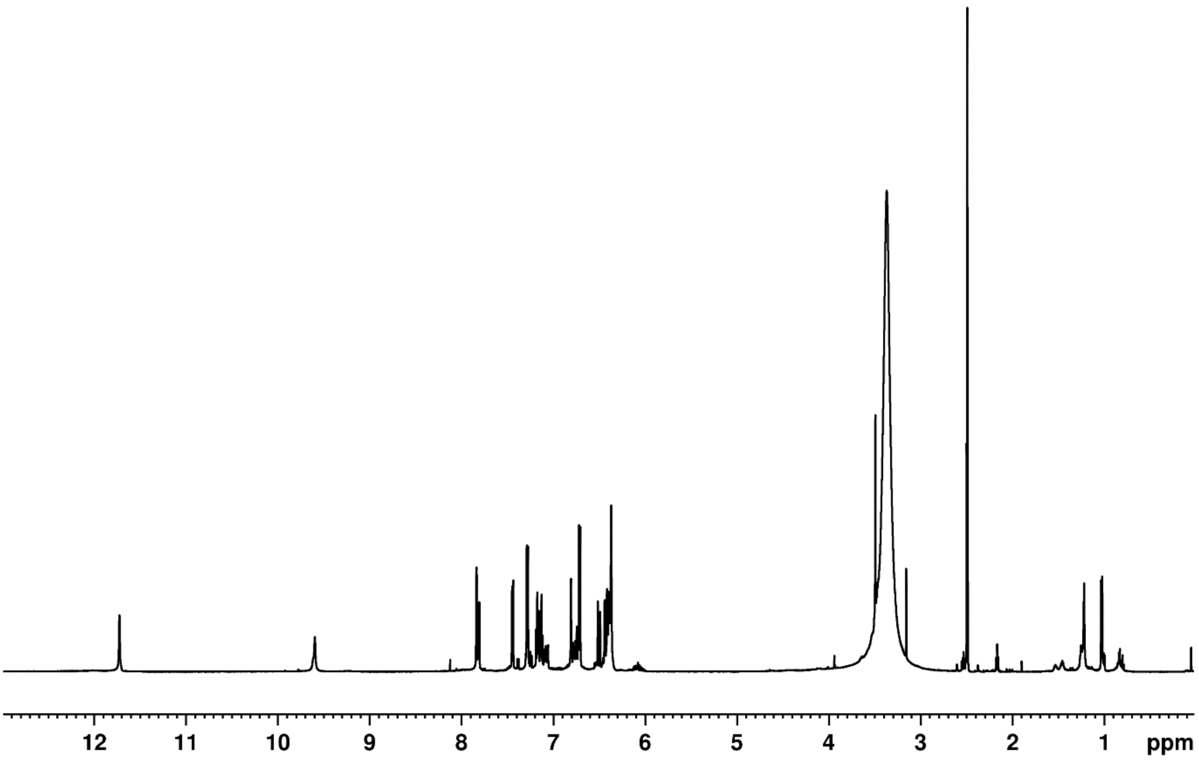

Figure S56. ^{1}H NMR spectrum of celluxanthene D (**4**) in DMSO-d_6 at 300K.

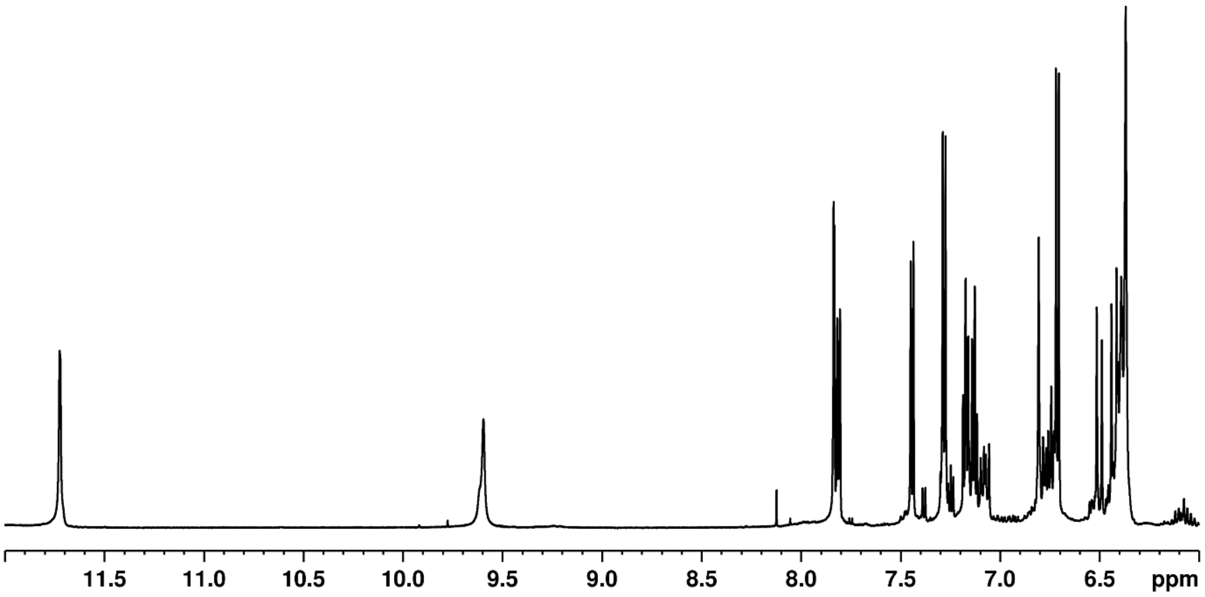

Figure S57. Extended ^{1}H NMR spectrum of celluxanthene D (**4**) in DMSO-d_6 at 300K.

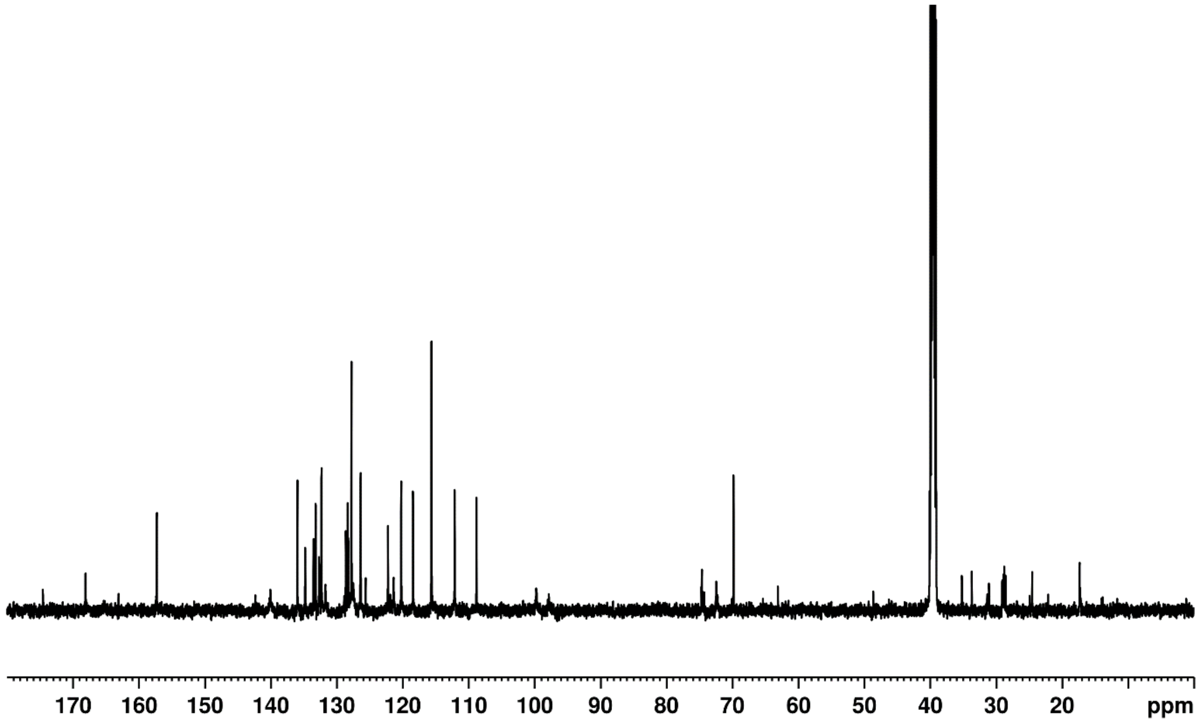

Figure S58. ^{13}C NMR spectrum of celluxanthene D (**4**) in DMSO-d_6 at 300K.

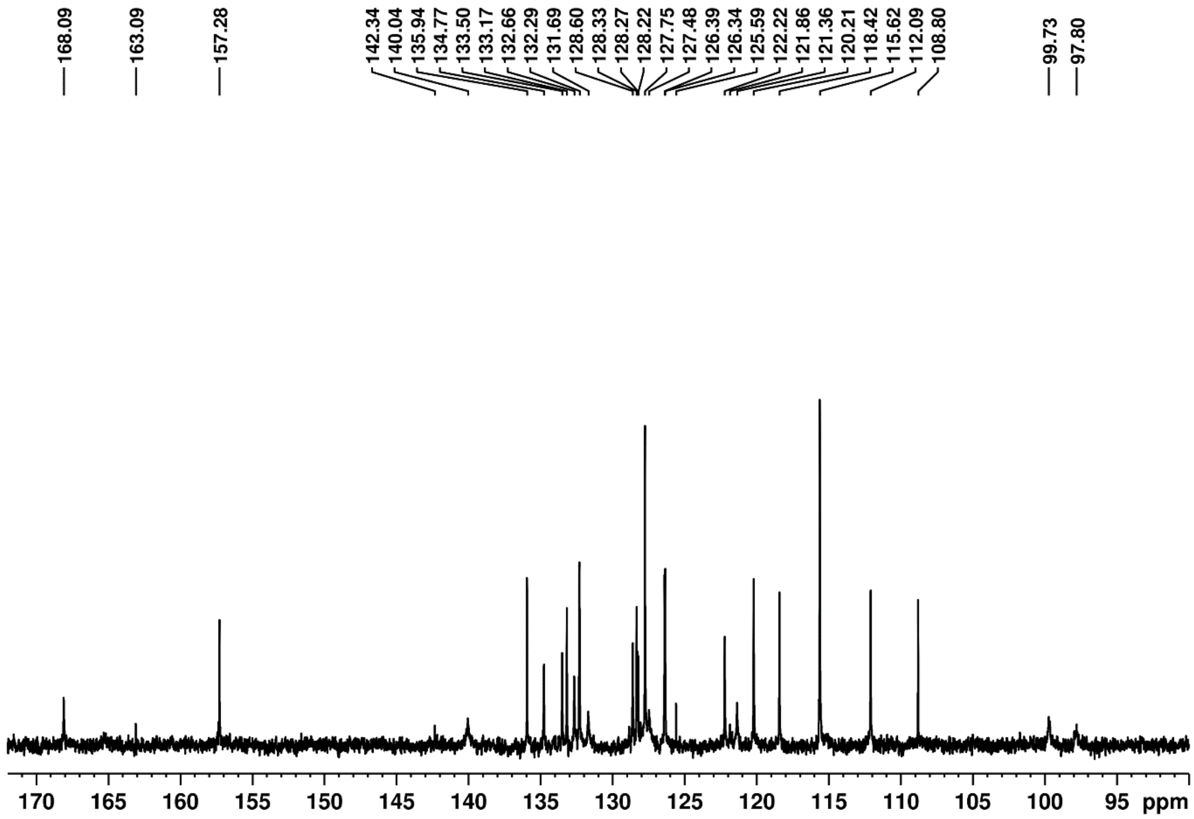

Figure S59. Extended ^{13}C NMR spectrum of celluxanthene D (**4**) in DMSO-d_6 at 300K.

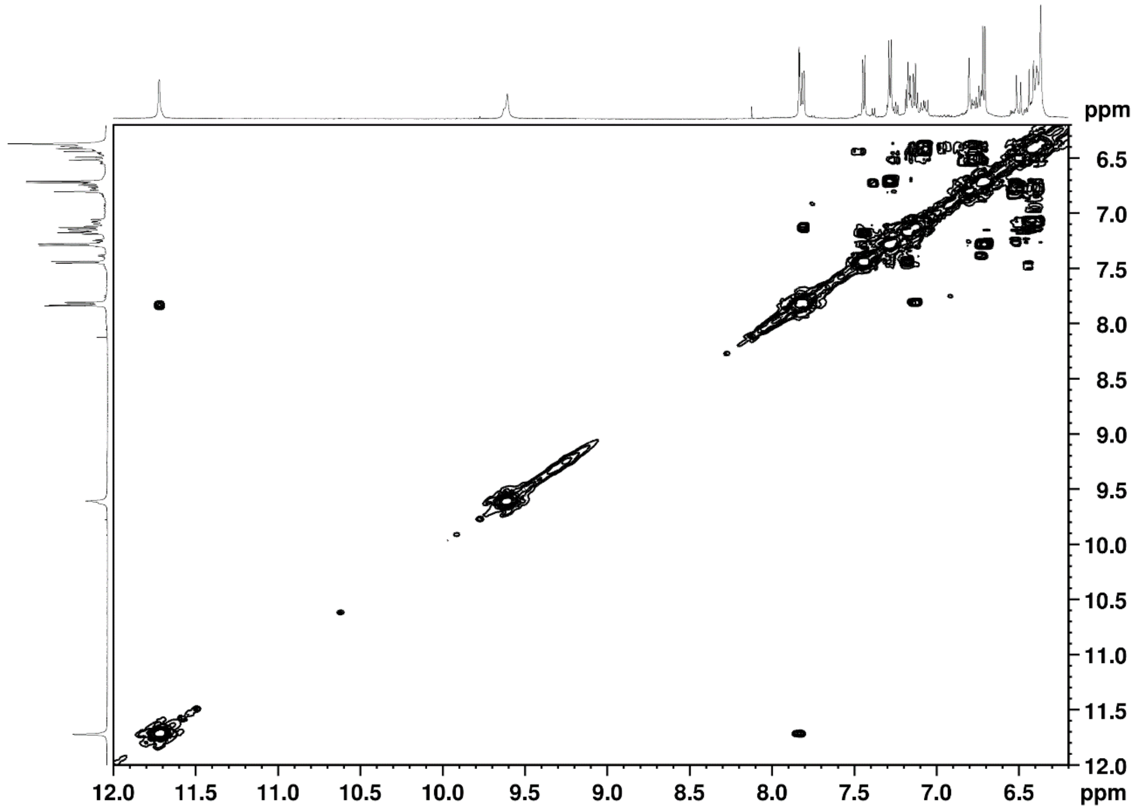

Figure S60. ^{1}H-^{1}H COSY spectrum of celluxanthene D (**4**) in DMSO-d_6 at 300K.

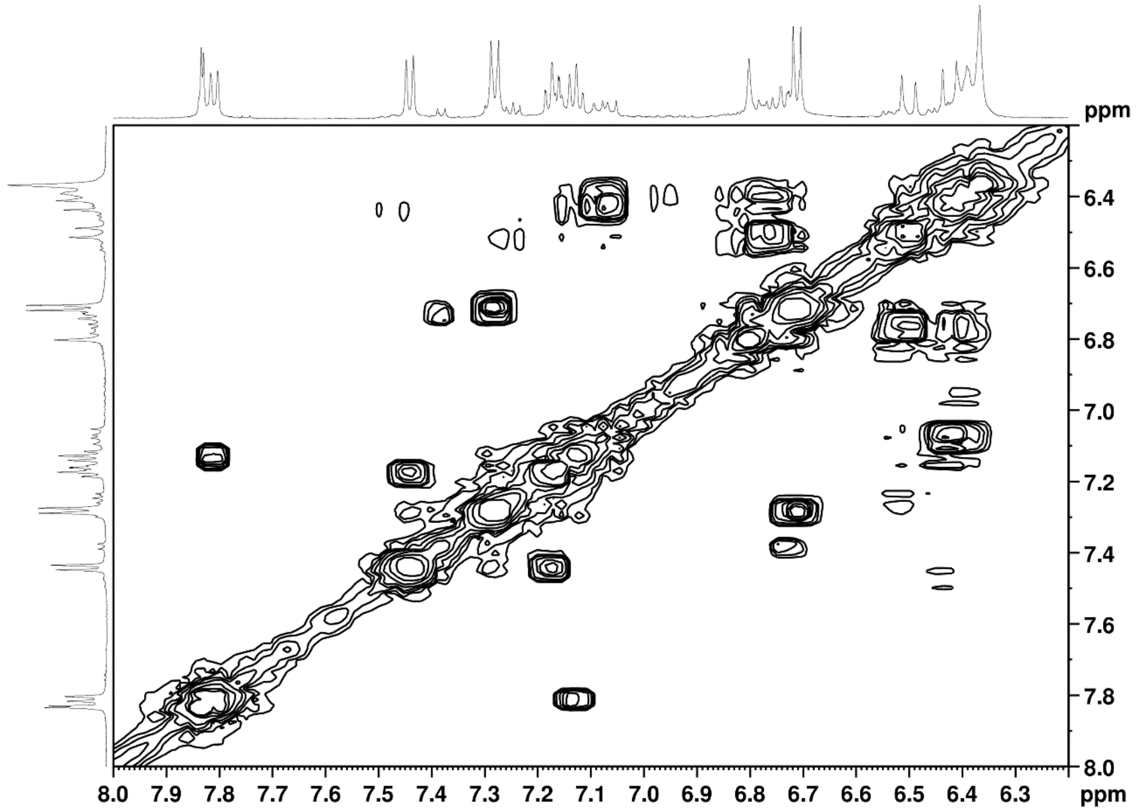

Figure S61. Extended ^{1}H-^{1}H COSY spectrum of celluxanthene D (**4**) in DMSO-d_6 at 300K.

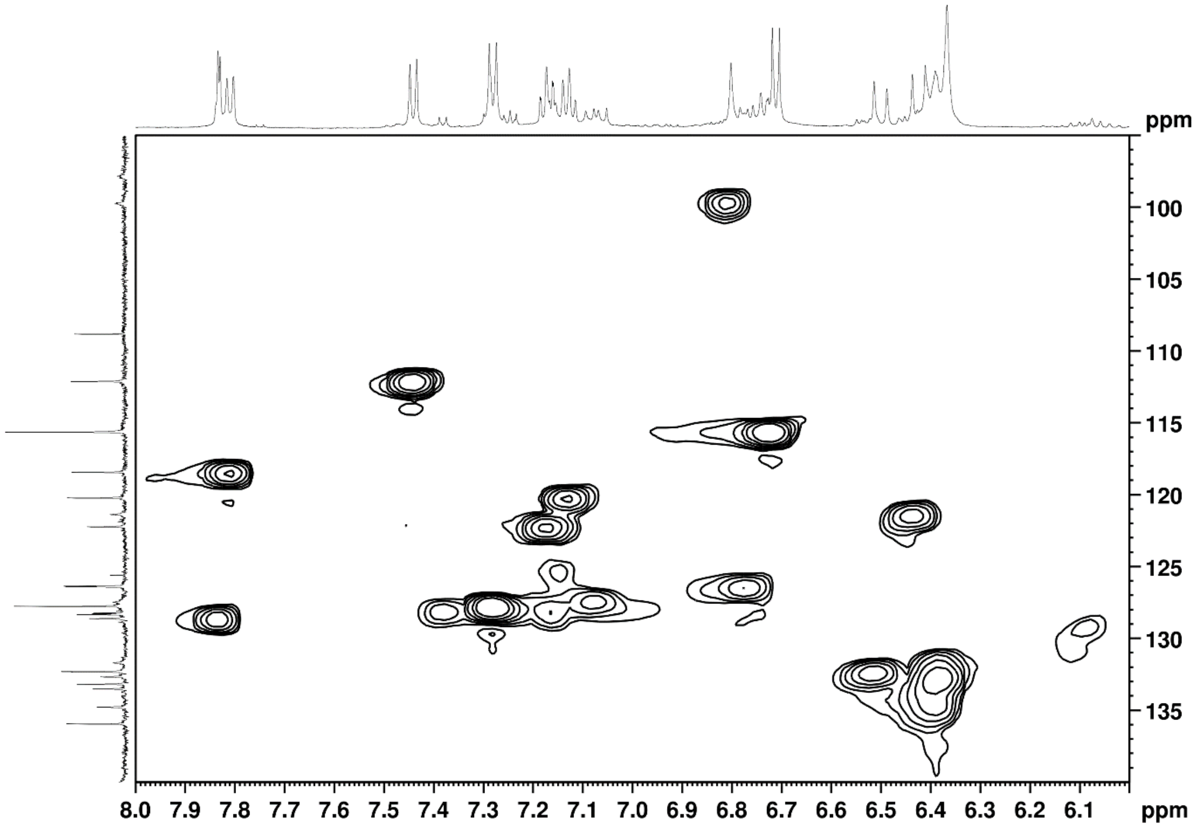

Figure S62. HSQC spectrum of celluxanthene D (**4**) in DMSO-d_6 at 300K.

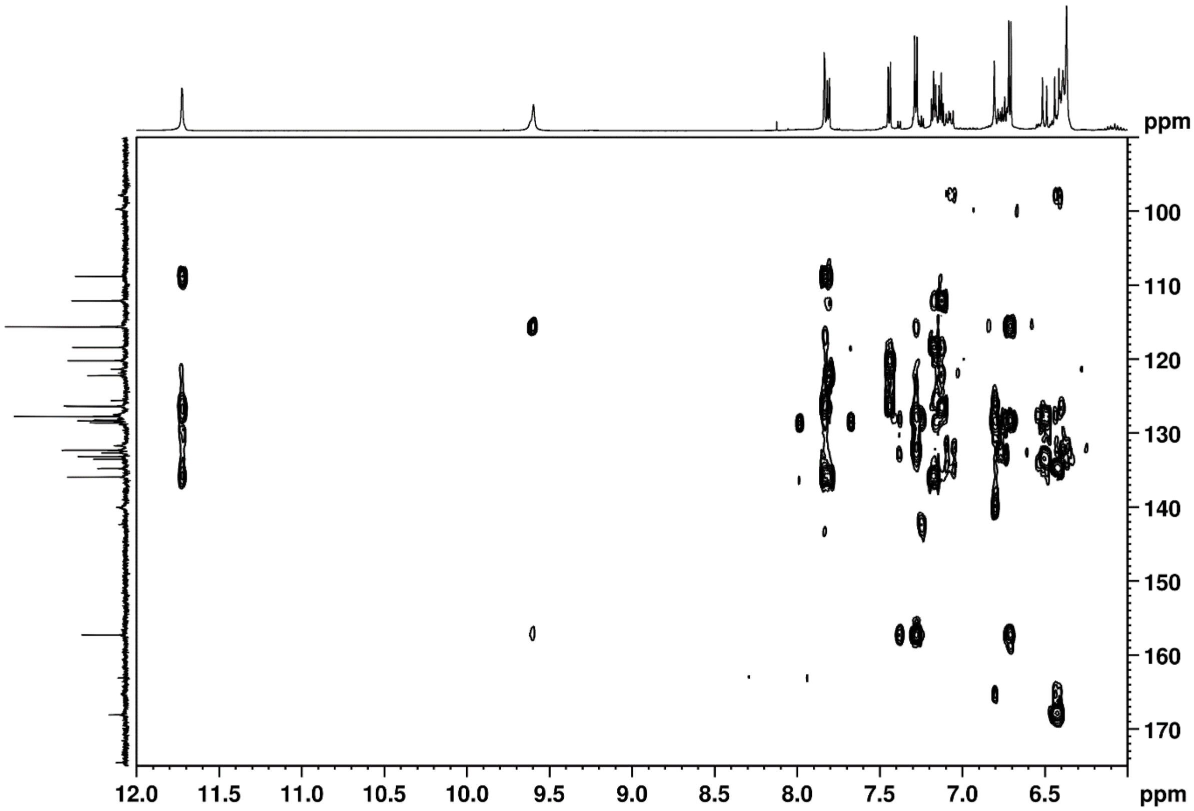

Figure S63. HMBC spectrum of celluxanthene D (**4**) in DMSO-d_6 at 300K.

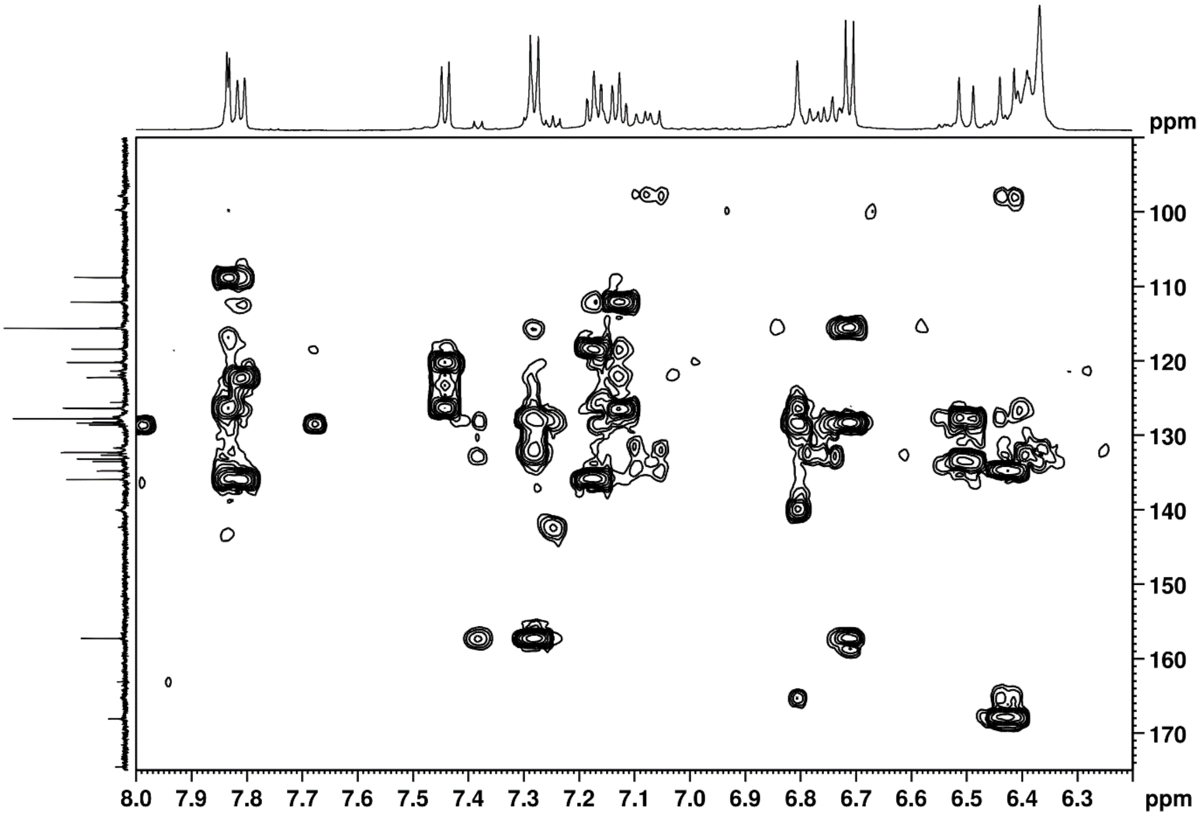

Figure S64. Extended HMBC spectrum of celluxanthene D (**4**) in DMSO-d_6 at 300K.

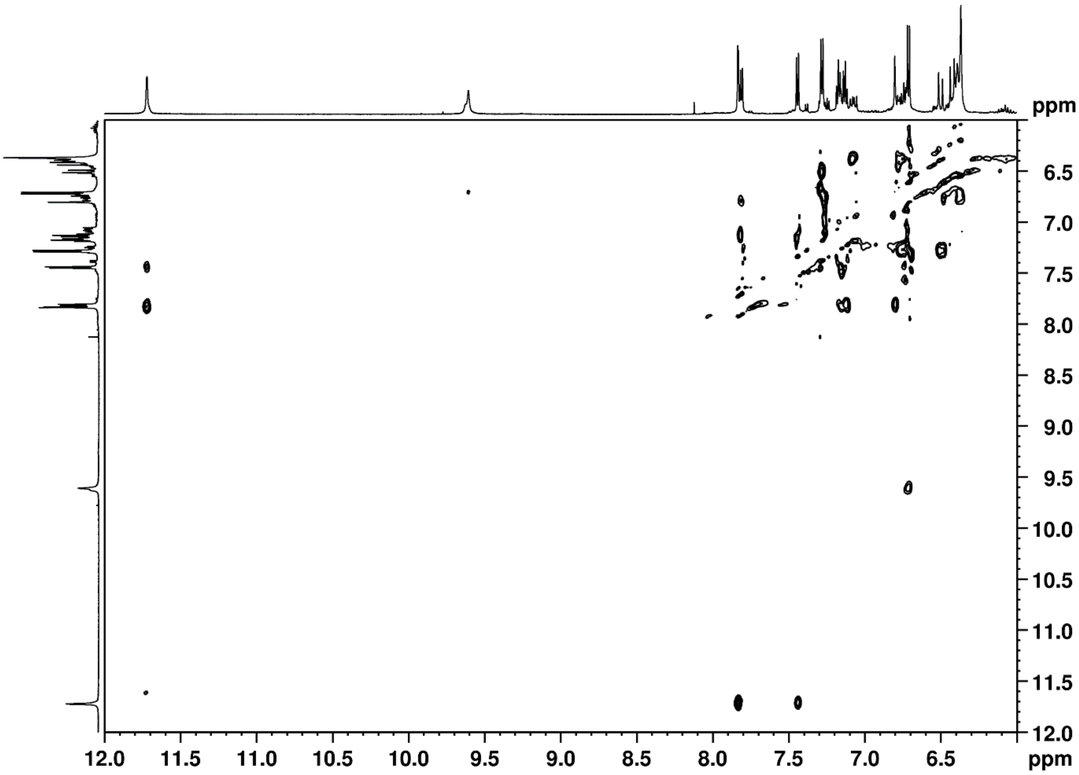

Figure S65. ROESY spectrum of celluxanthene D (**4**) in DMSO-d_6 at 300K.

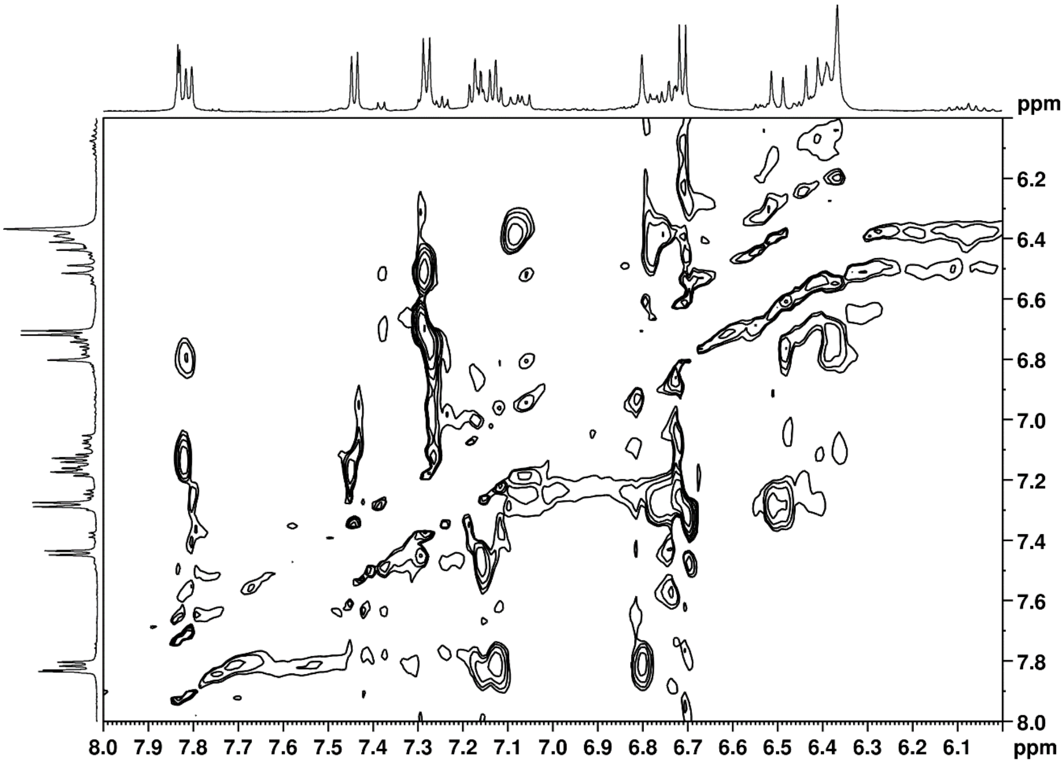

Figure S66. Extended ROESY spectrum of celluxanthene D (**4**) in DMSO-d_6 at 300K.

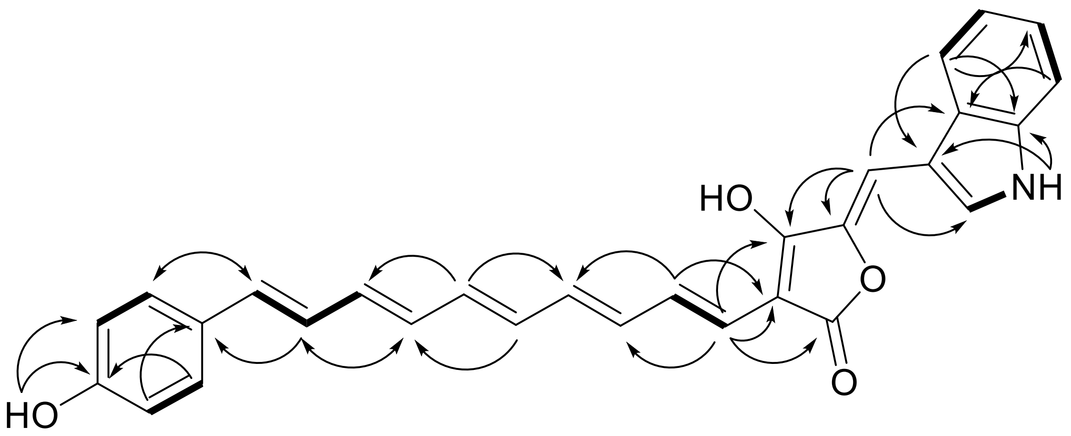

Figure S67. Selected ^{1}H-^{1}H COSY (bold lines) and HMBC (arrows) correlations of **4**.

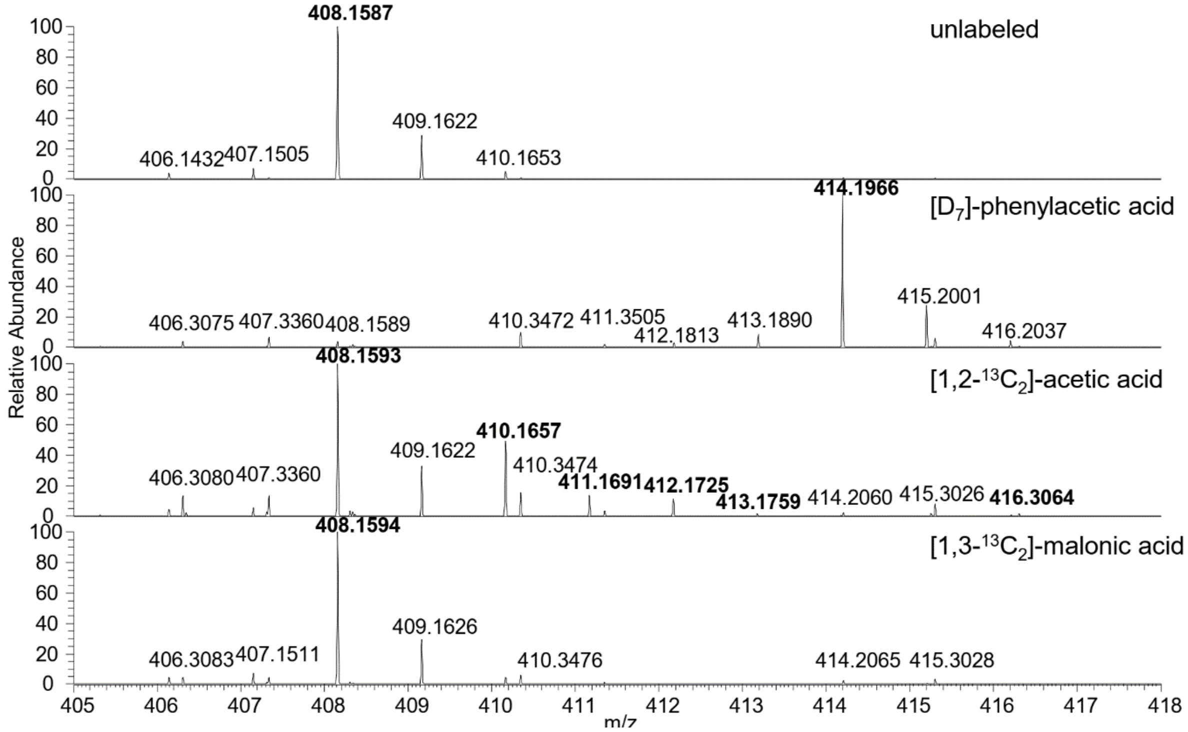

Figure S68. Positive HR-MS profile of celluxanthene A (**1**) of EtOAc extracts obtained from isotope labeled substrate feeding experiments.

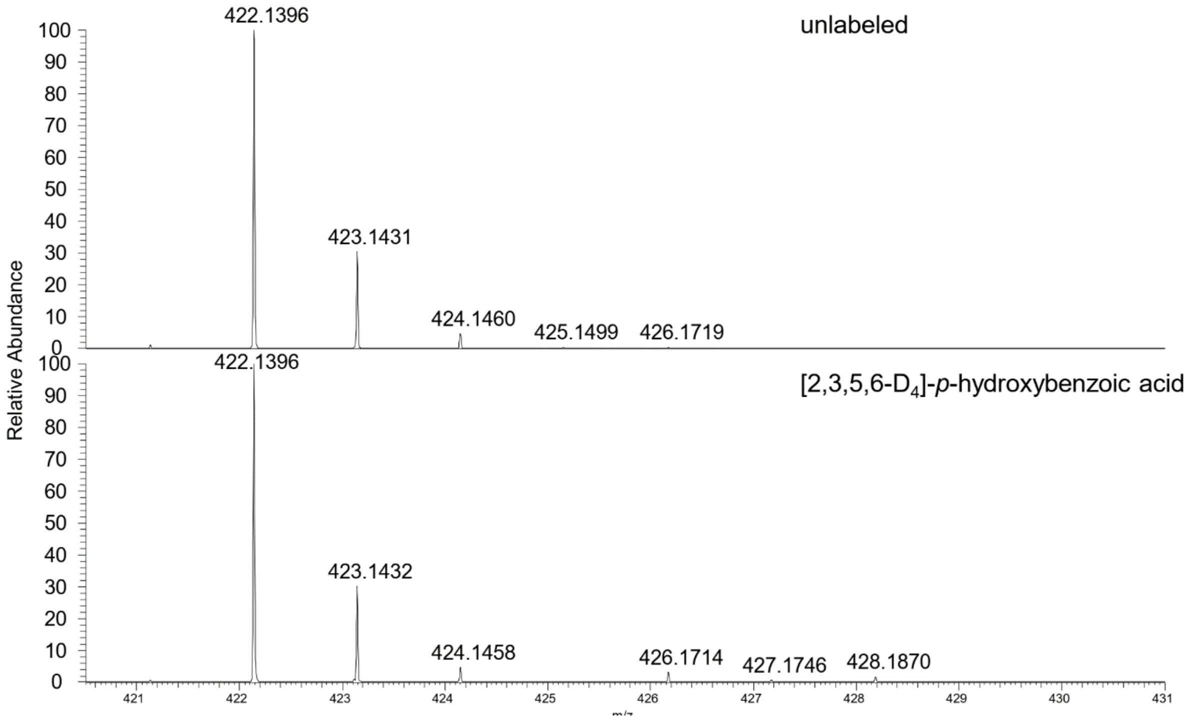

Figure S69. Positive HR-MS profile of celluxanthene B (**2**) of EtOAc extracts obtained from [2,3,5,6-D_4]-*p*-hydroxybenzoic acid feeding experiments.

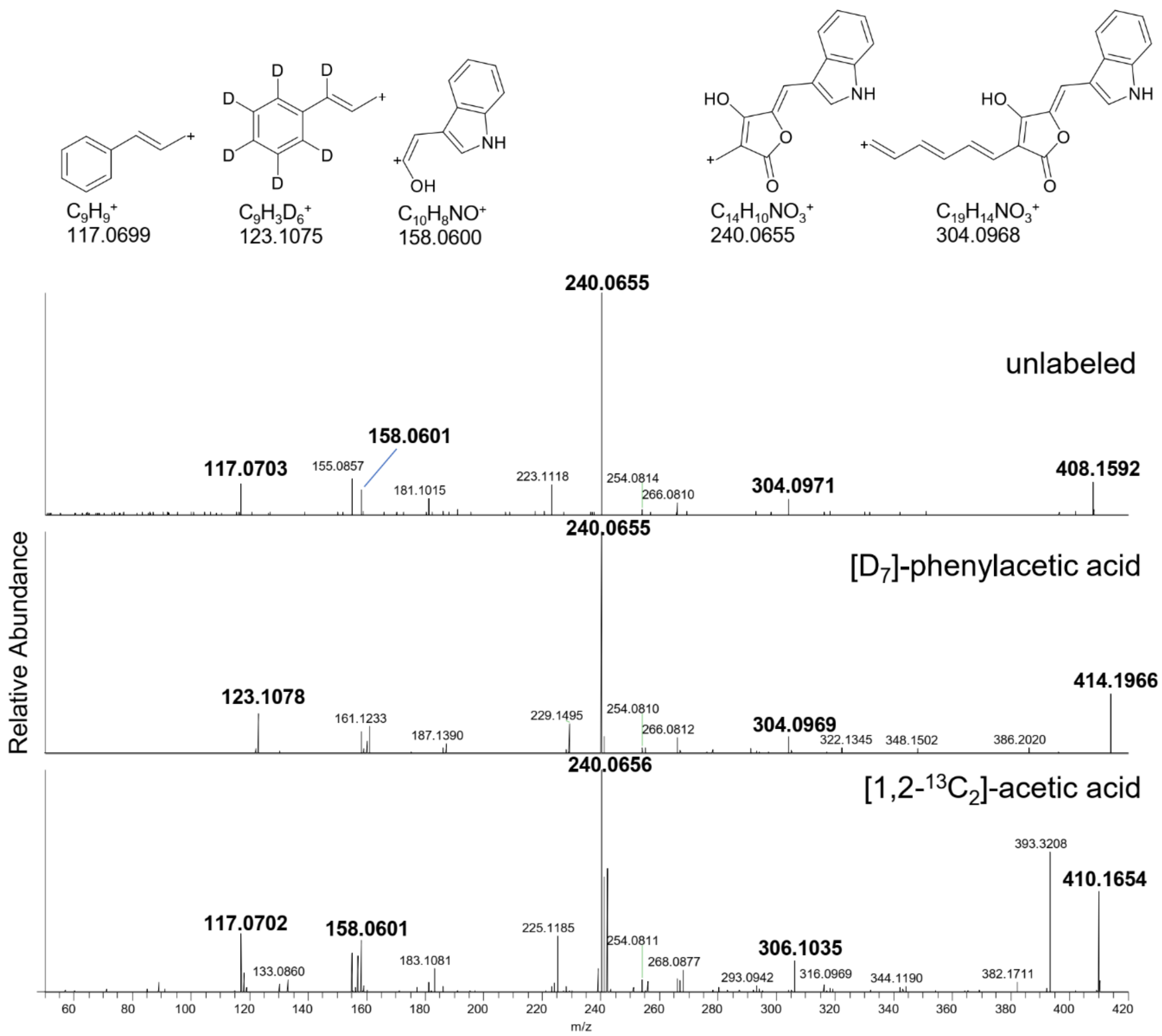

Figure S70. Positive HR-MS/MS profile of celluxanthene A (**1**) of EtOAc extracts obtained from isotope labeled substrates feeding experiments.

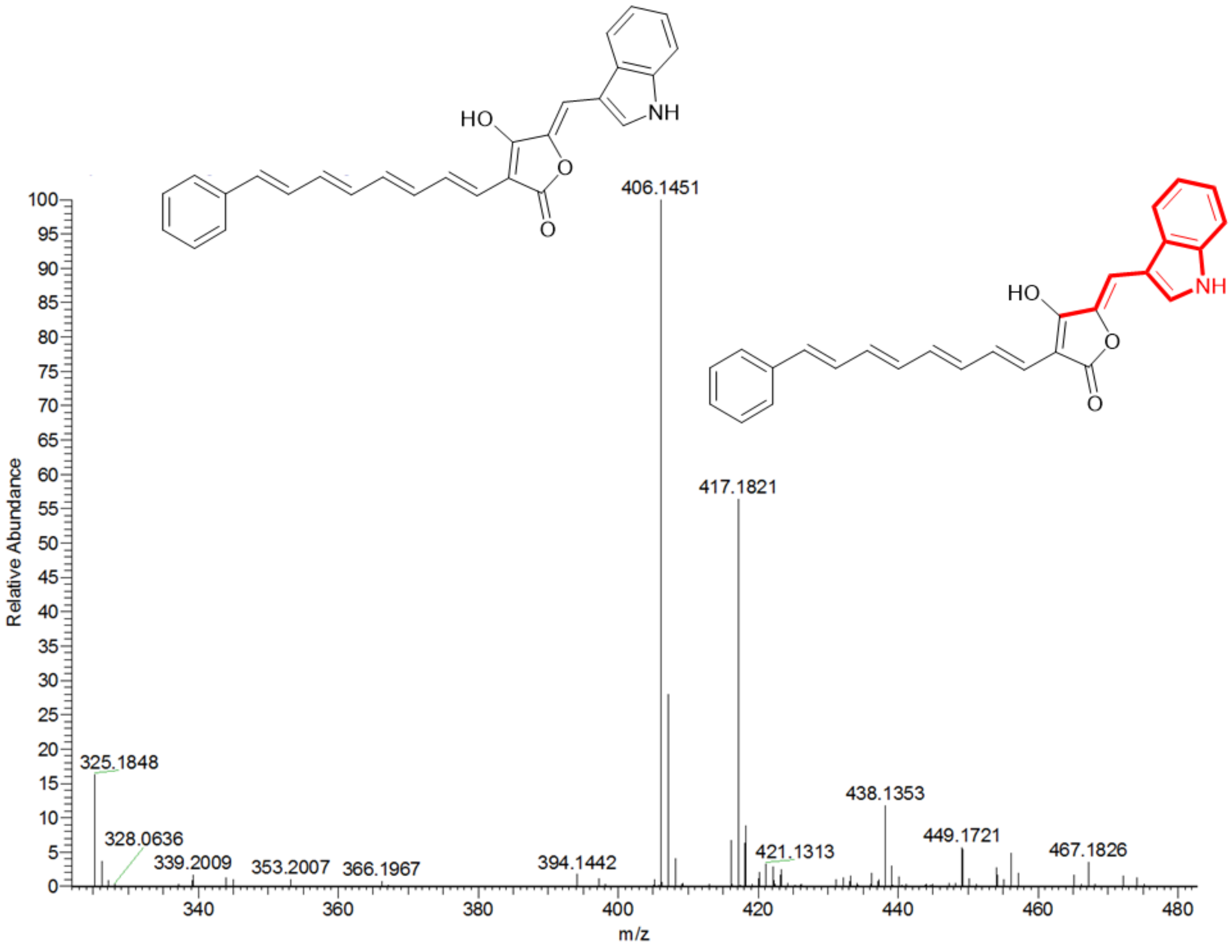

Figure S71. Negative HR-MS profile of celluxanthene A (**1**) of EtOAc extracts obtained from [$^{13}C_{11}$]-L-tryptophan feeding experiments. Red color indicates incorporated tryptophan.

A B

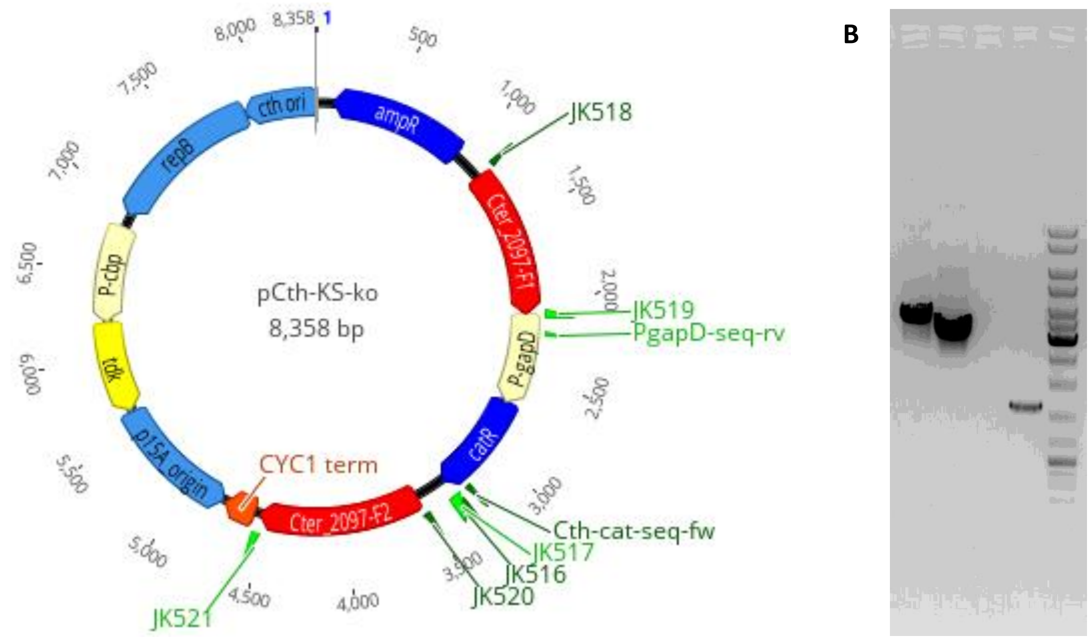

Figure S72. Generation of *C. thermocellum* Δ*cex* mutant. A) Used plasmid map of pCth-KS-ko. B) Uncropped gel for Figure 2D.

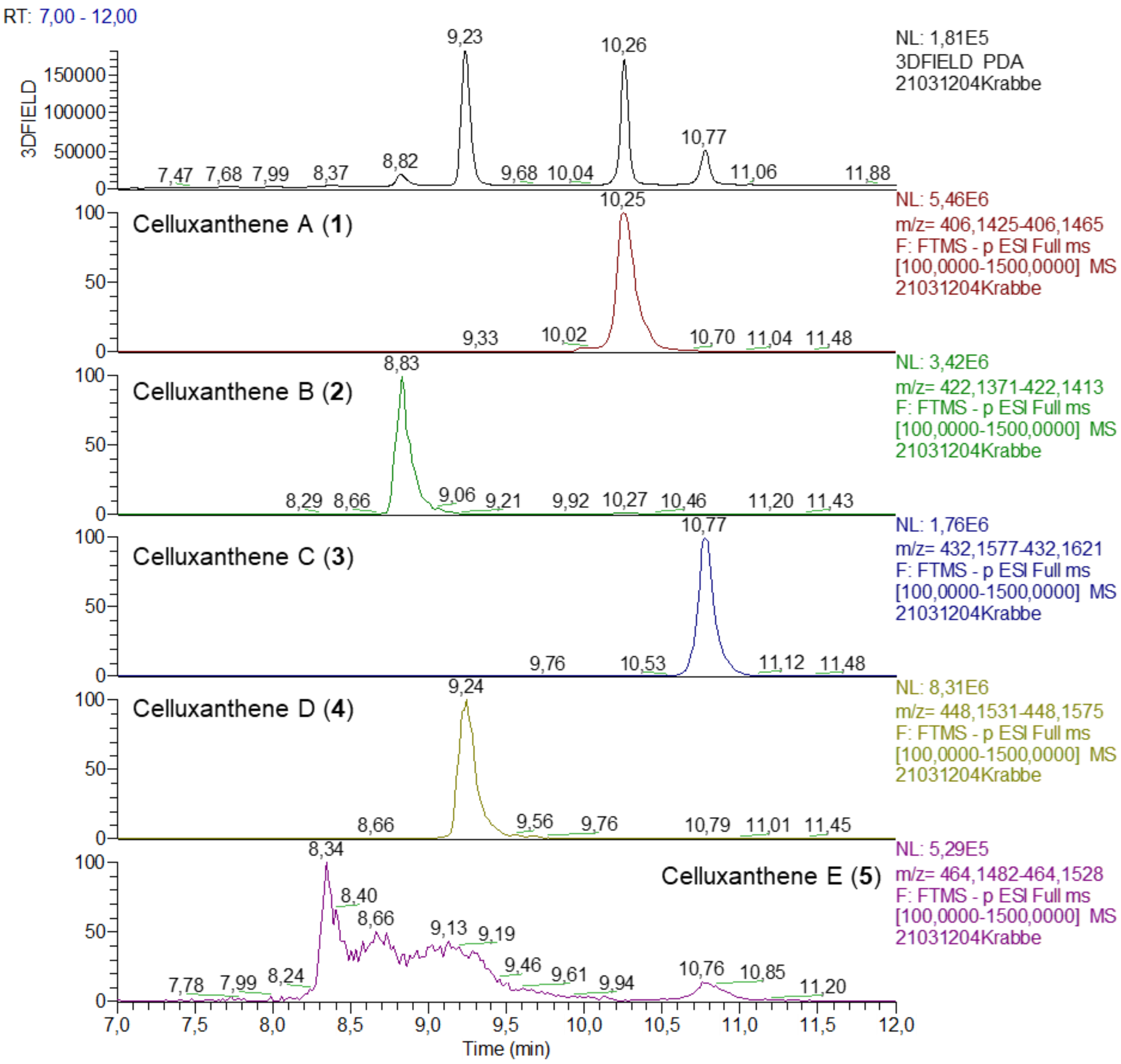

Figure S73. PDA (420–450 nm) and (–) EIC profile of celluxanthene A–E (**1**–**5**) of *Clostridium thermocellum* DSM 1313 extract.

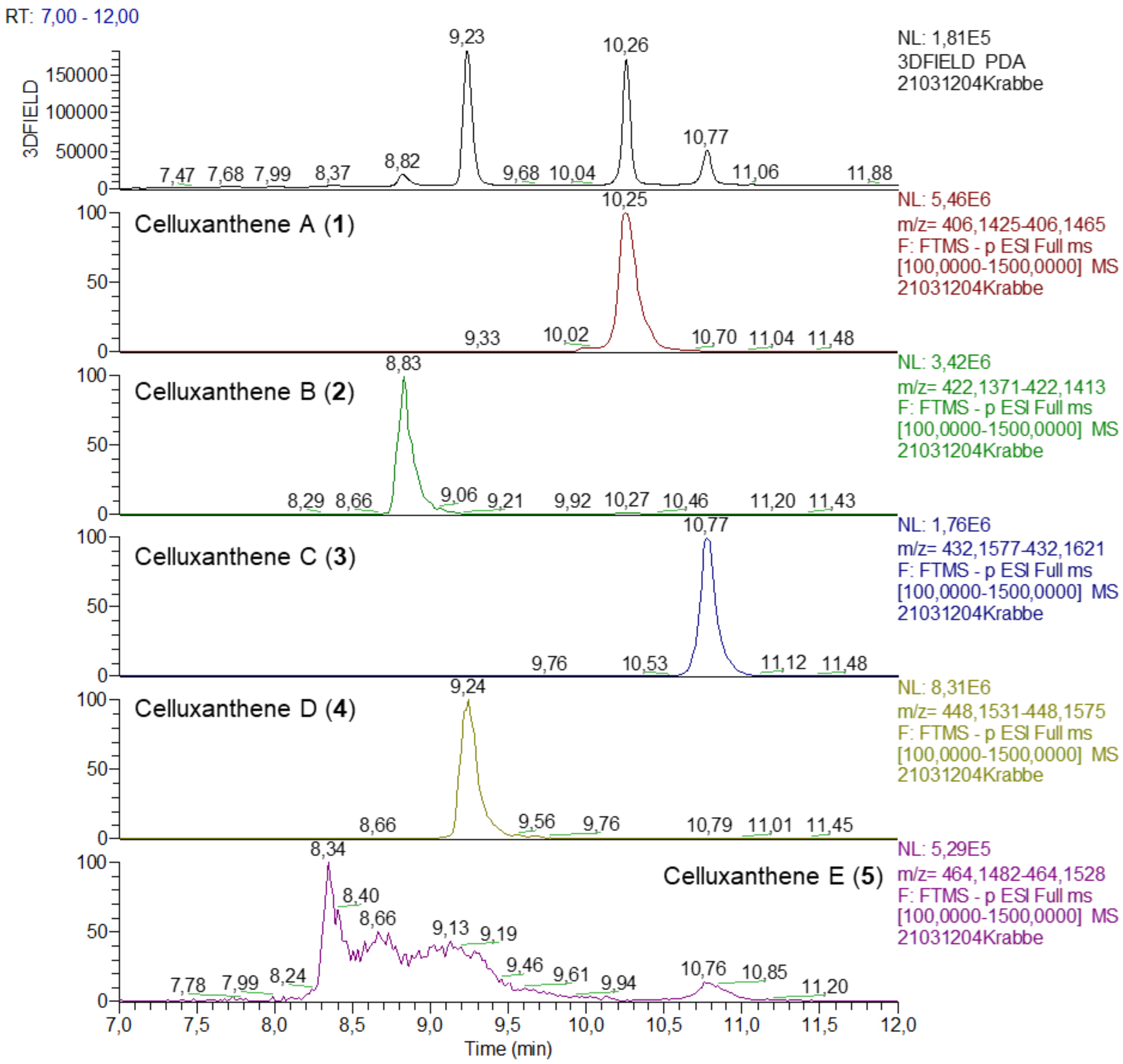

Figure S74. PDA (420–450 nm) and (–)EIC profile of celluxanthene A–E (**1**–**5**) of *Clostridium thermocellum* DSM 4150 extract.

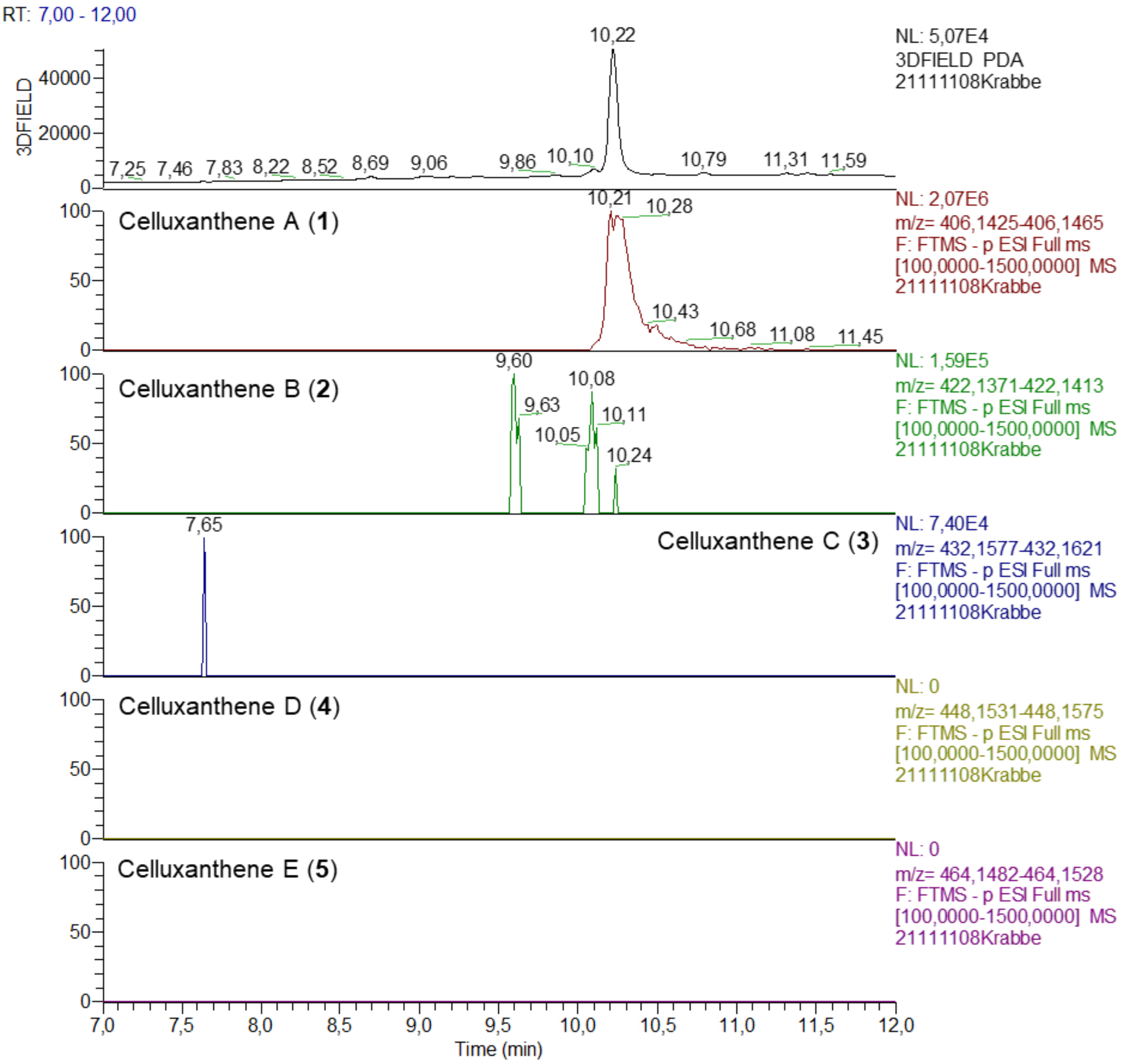

Figure S75. PDA (420–450 nm) and (–)EIC profile of celluxanthene A–E (**1**–**5**) of *Clostridium straminisolvens* DSM 16021 extract.

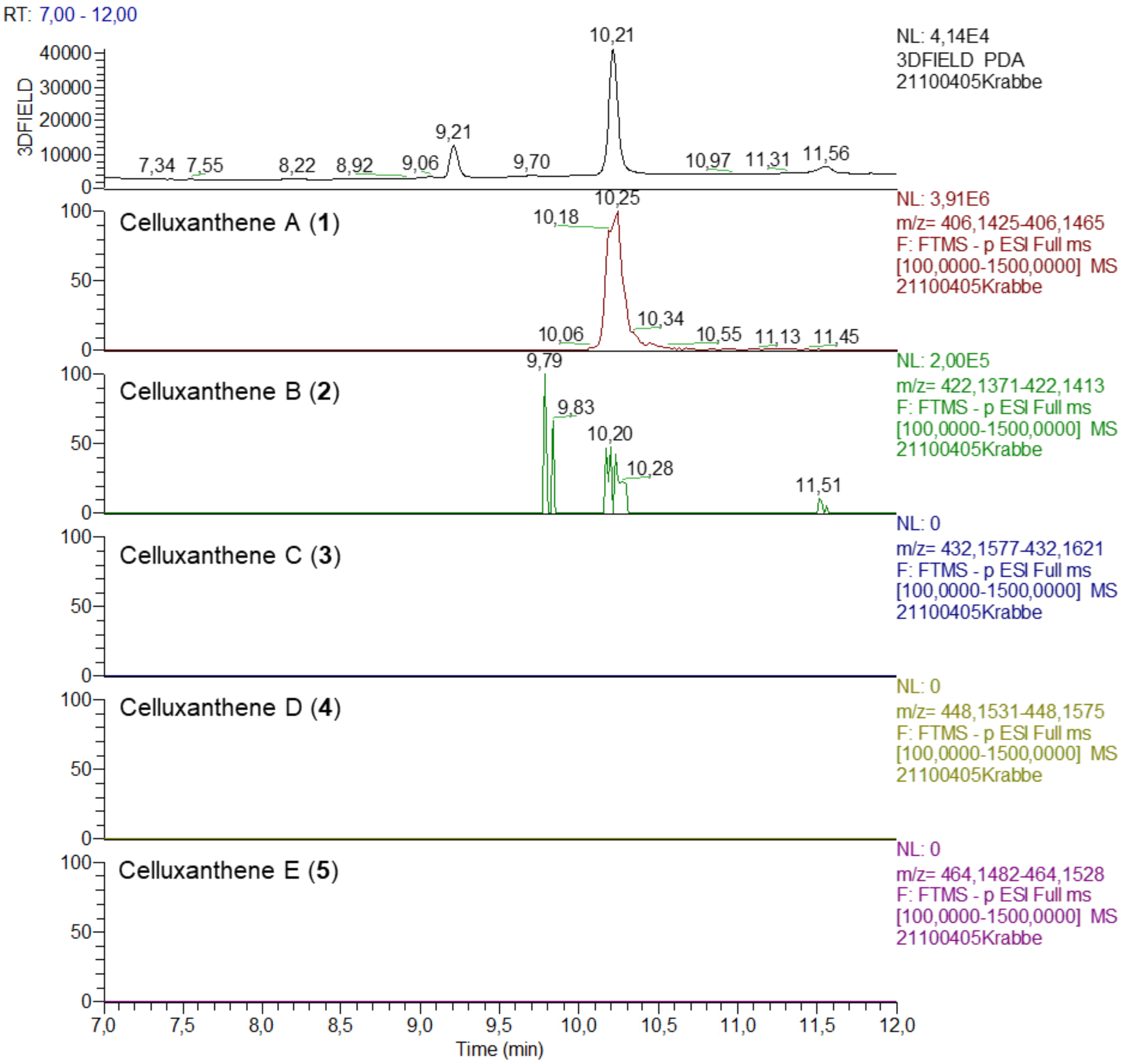

Figure S76. PDA (420–450 nm) and (–)EIC profile of celluxanthene A–E (**1**–**5**) of *Acetivibrio alkalicellulosi* DSM 17461 extract.

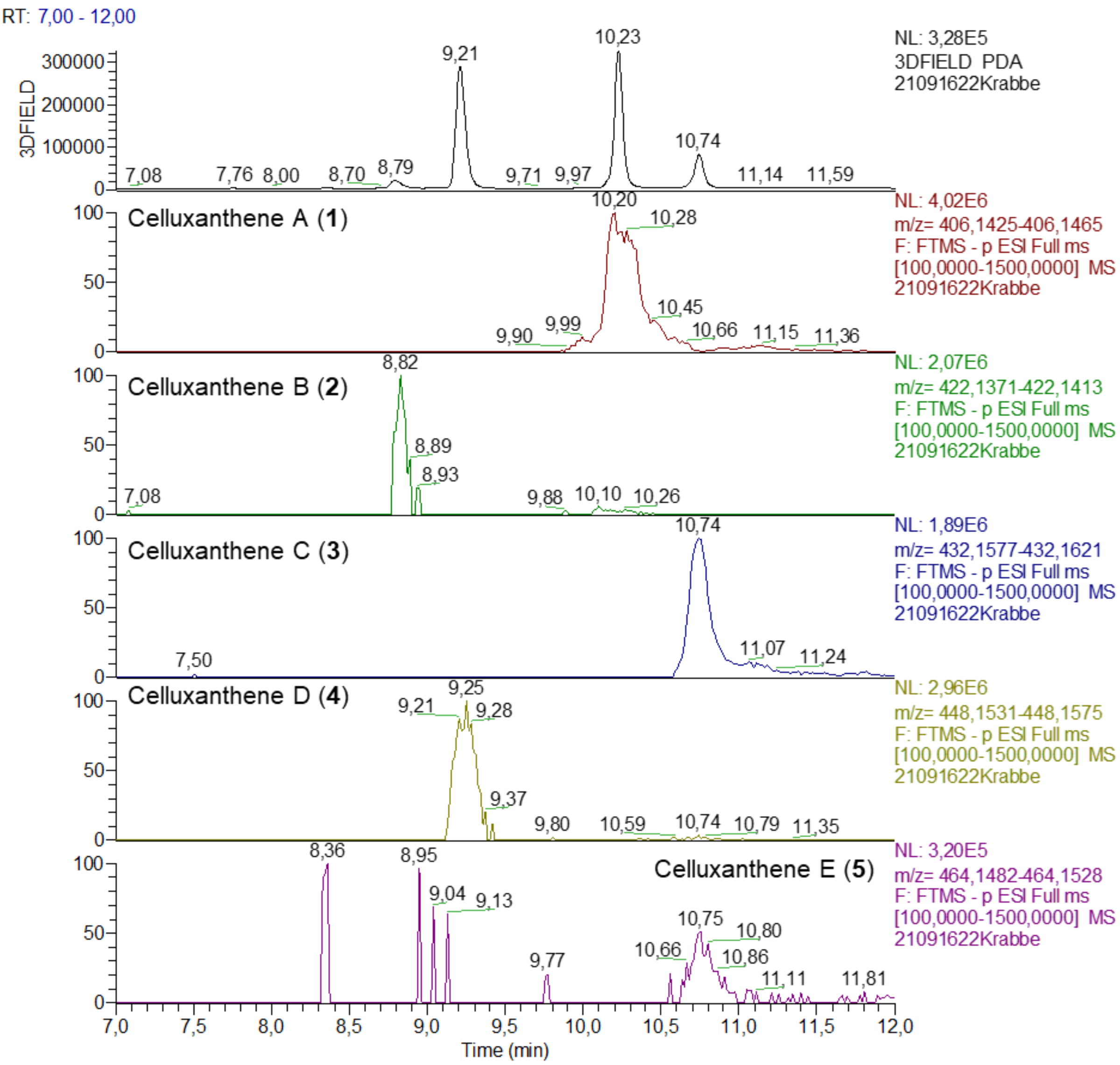

Figure S77. PDA (420–450 nm) and (–)EIC profile of celluxanthene A–E (**1**–**5**) of *Acetivibrio saccincola* DSM 101079 extract.

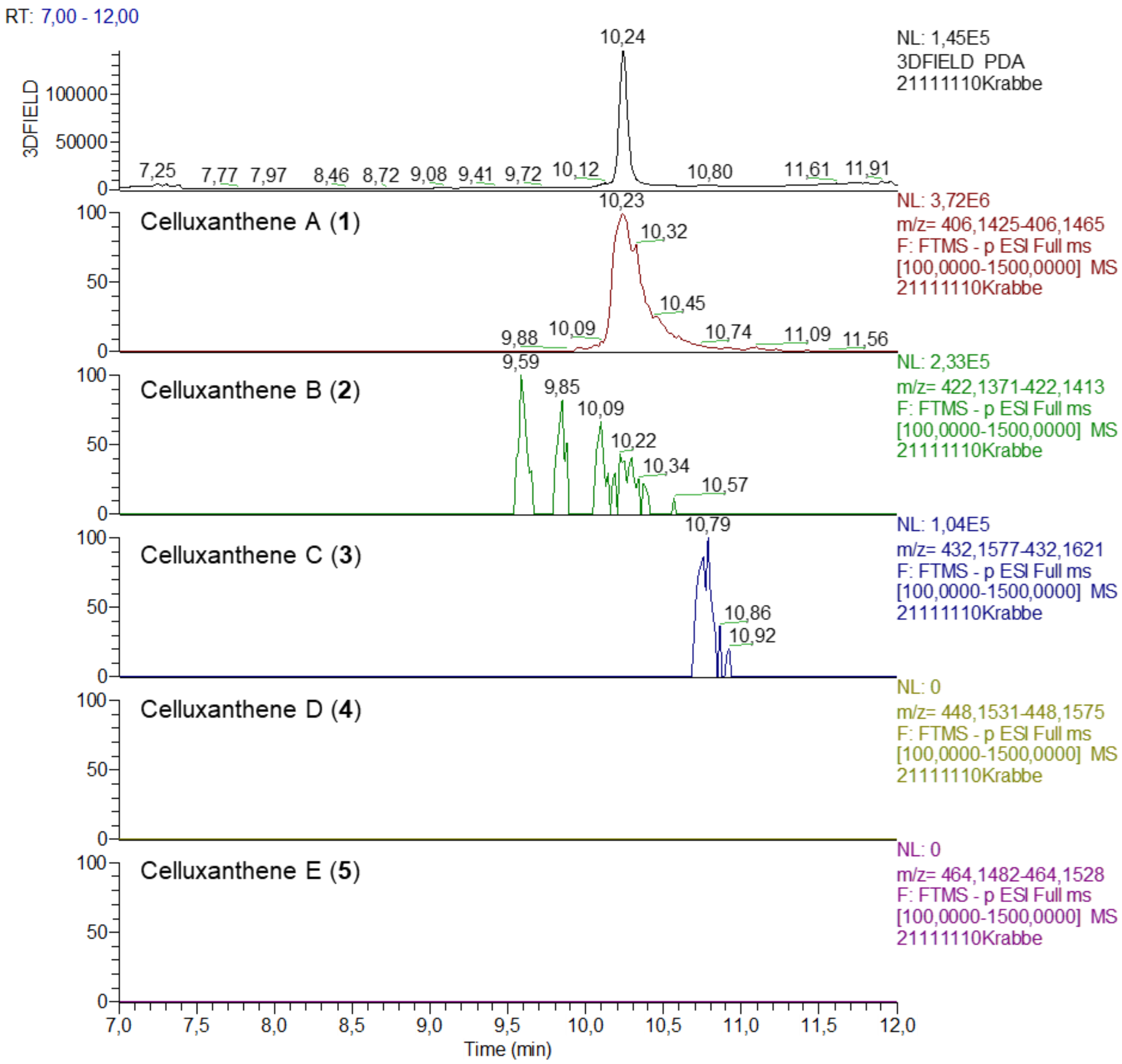

Figure S78. PDA (420–450 nm) and (–)EIC profile of celluxanthene A–E (**1**–**5**) of *Acetivibrio mesophilus* DSM 107956 extract.

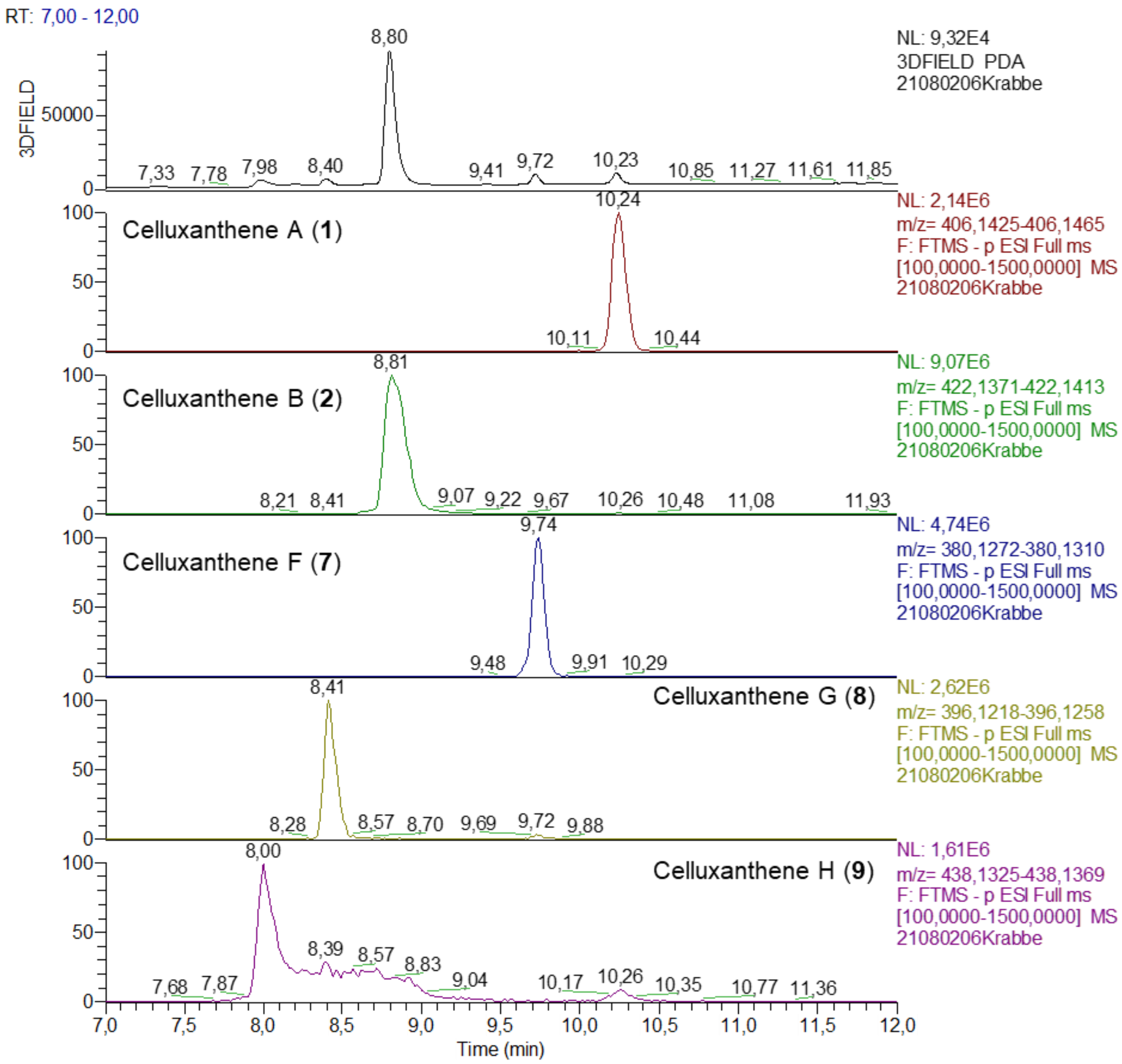

Figure S79. PDA (420–450 nm) and (–)EIC profile of celluxanthene A–B (**1**–**2**) and F–H (**7**–**9**) of *Pseudobacteroides cellulosolvens* DSM 2933 extract.

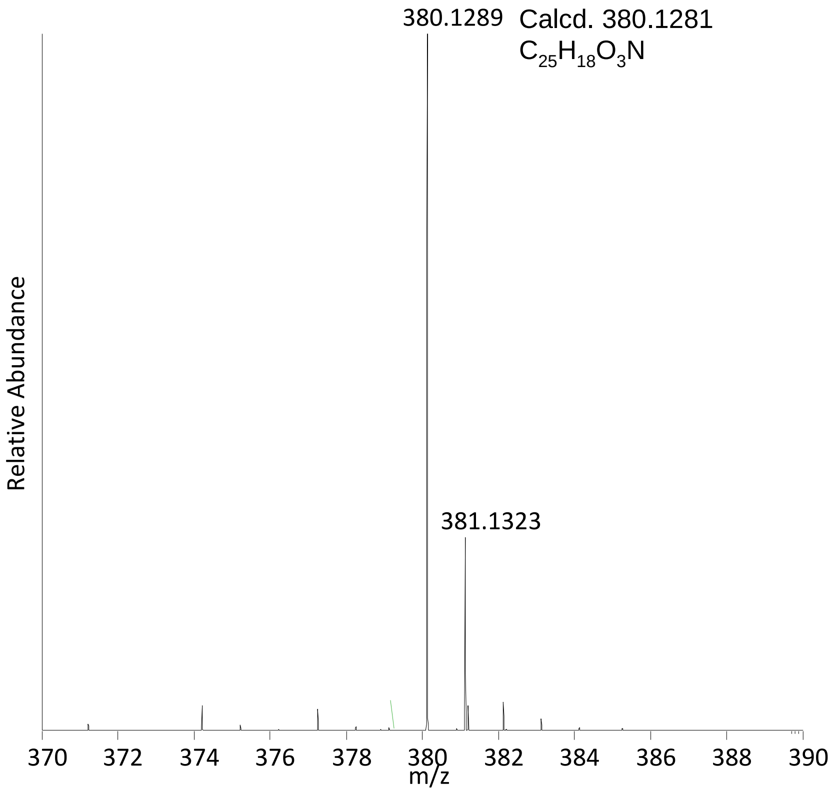

Figure S80. Negative HR-MS spectrum of celluxanthene F (**7**).

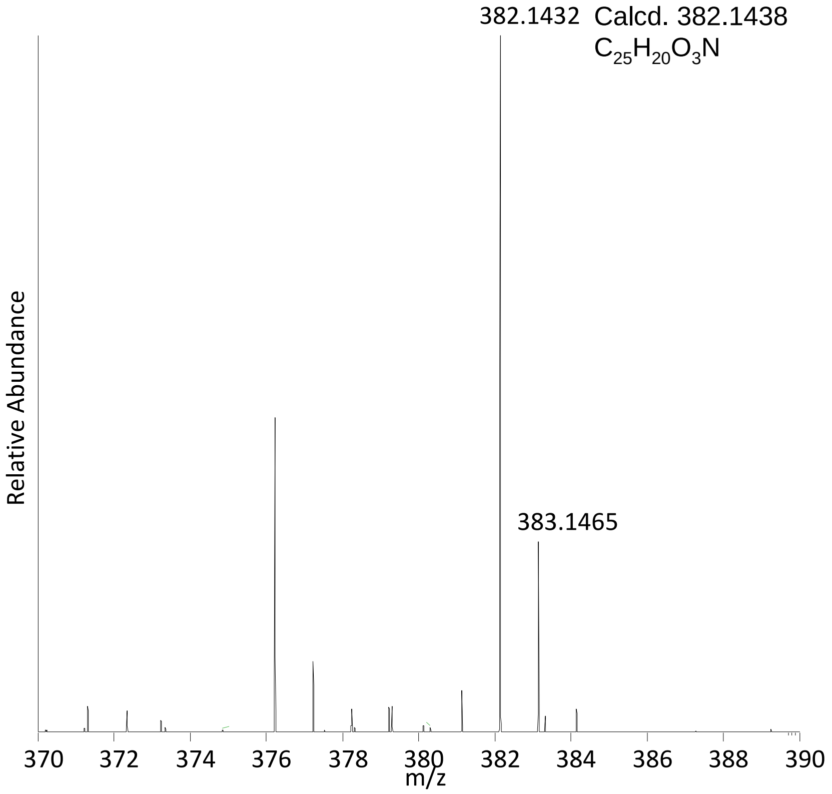

Figure S81. Positive HR-MS spectrum of celluxanthene F (**7**).

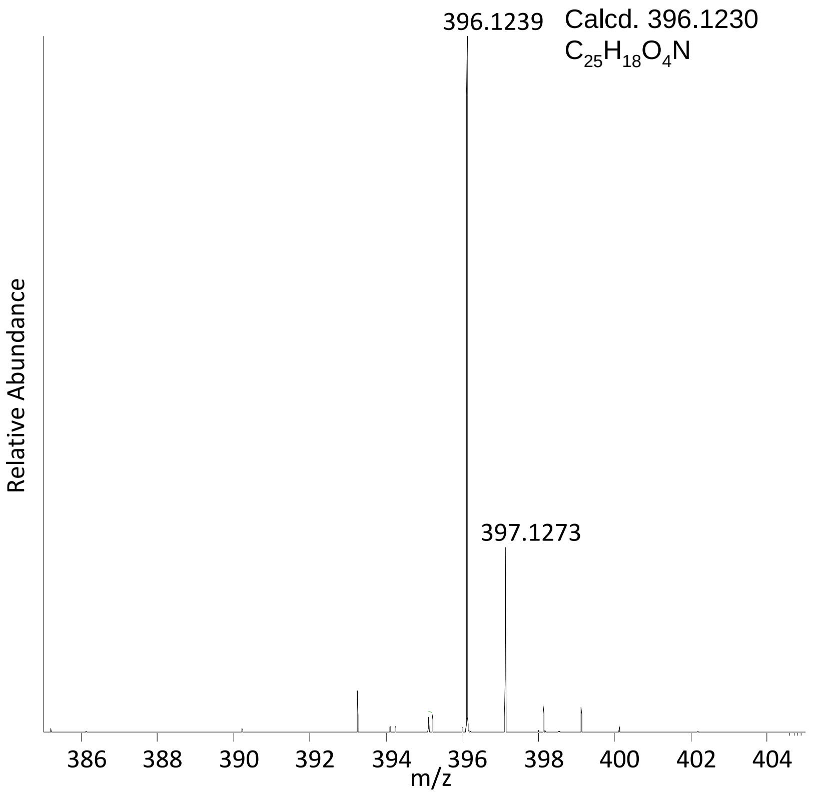

Figure S82. Negative HR-MS spectrum of celluxanthene G (**8**).

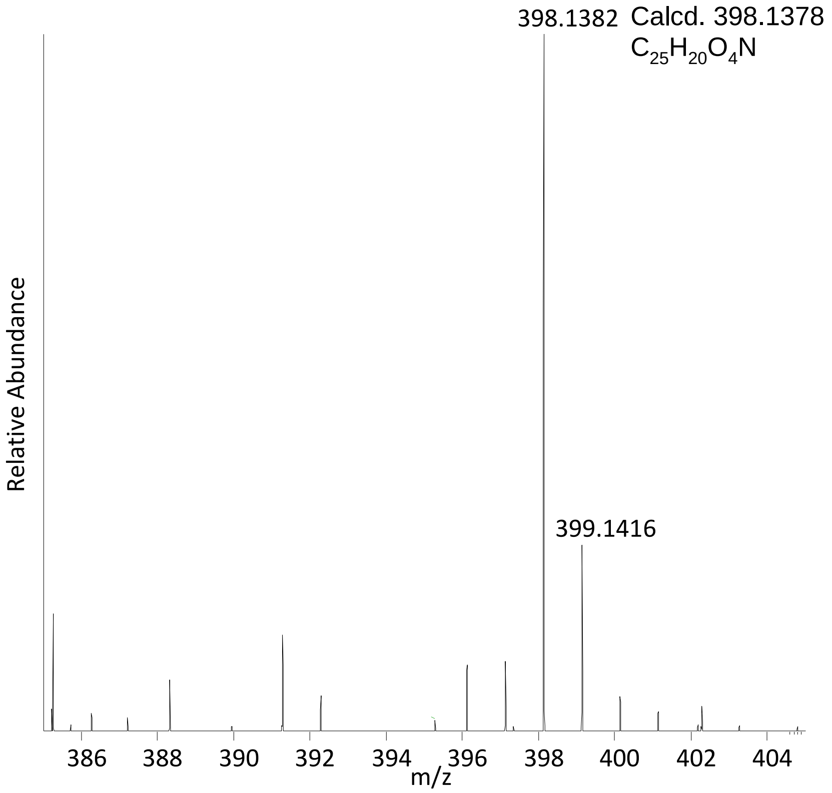

Figure S83. Positive HR-MS spectrum of celluxanthene G (**8**).

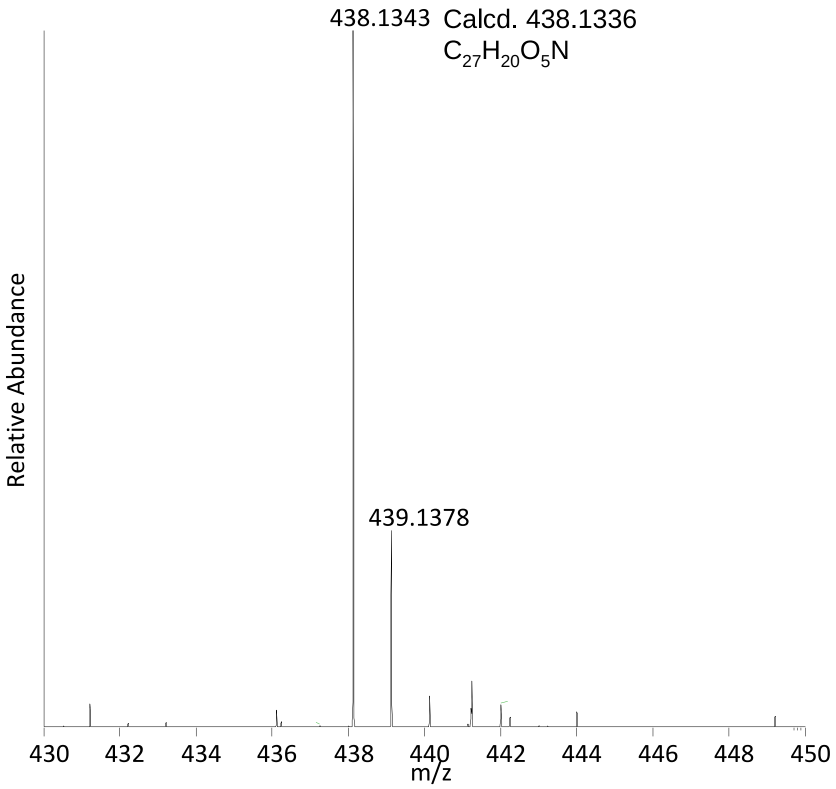

Figure S84. Negative HR-MS spectrum of celluxanthene H (**9**).

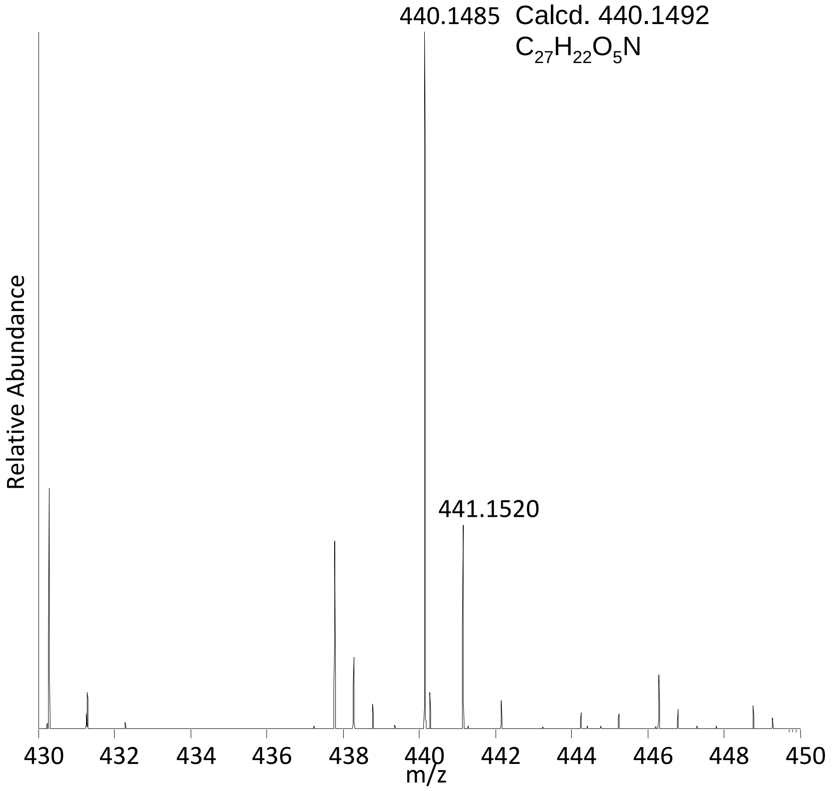

Figure S85. Positive HR-MS spectrum of celluxanthene H (**9**).

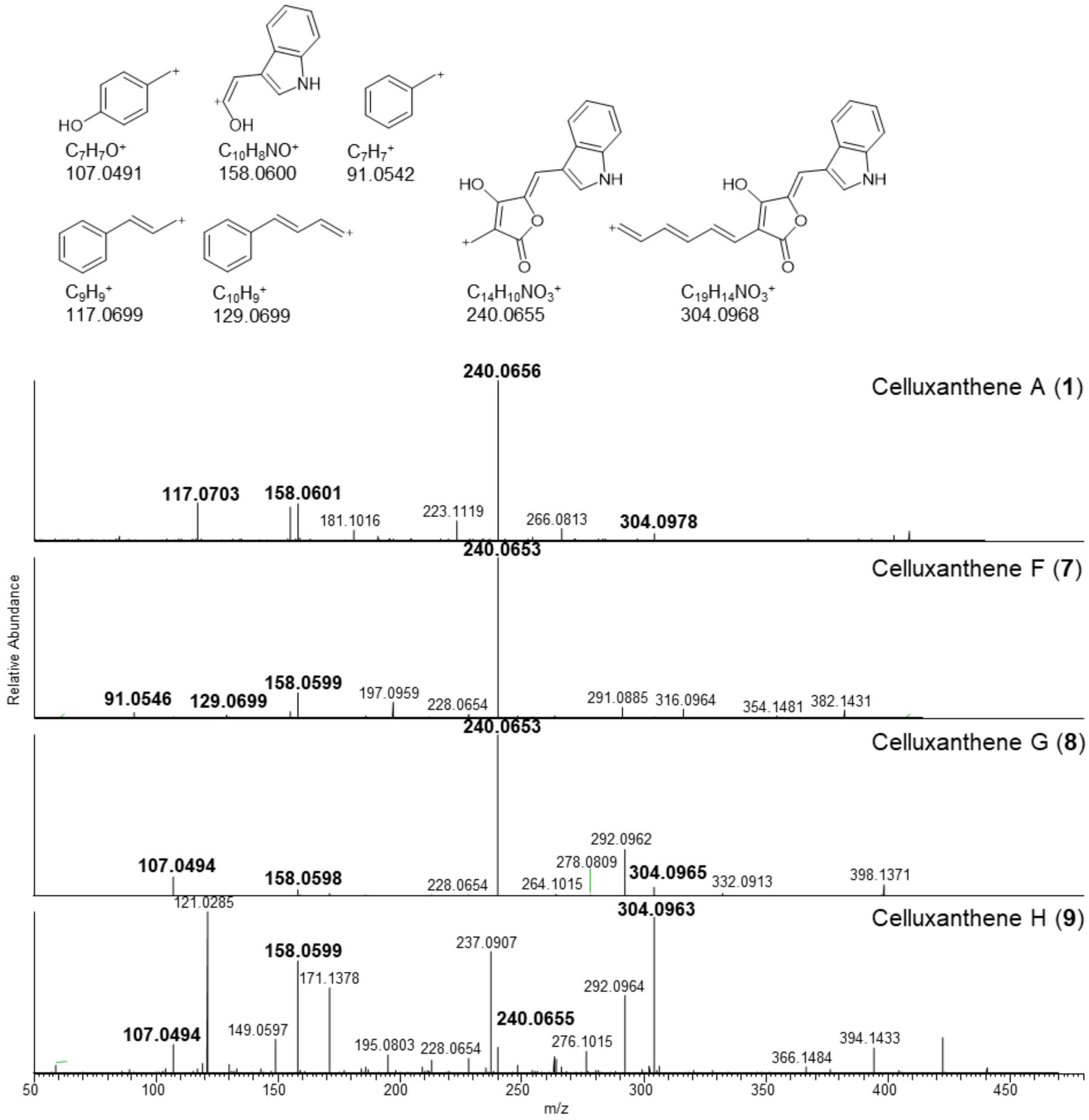

Figure S86. Positive HR-MS/MS profile of celluxanthene A, F–H (**1**, **7–9**).

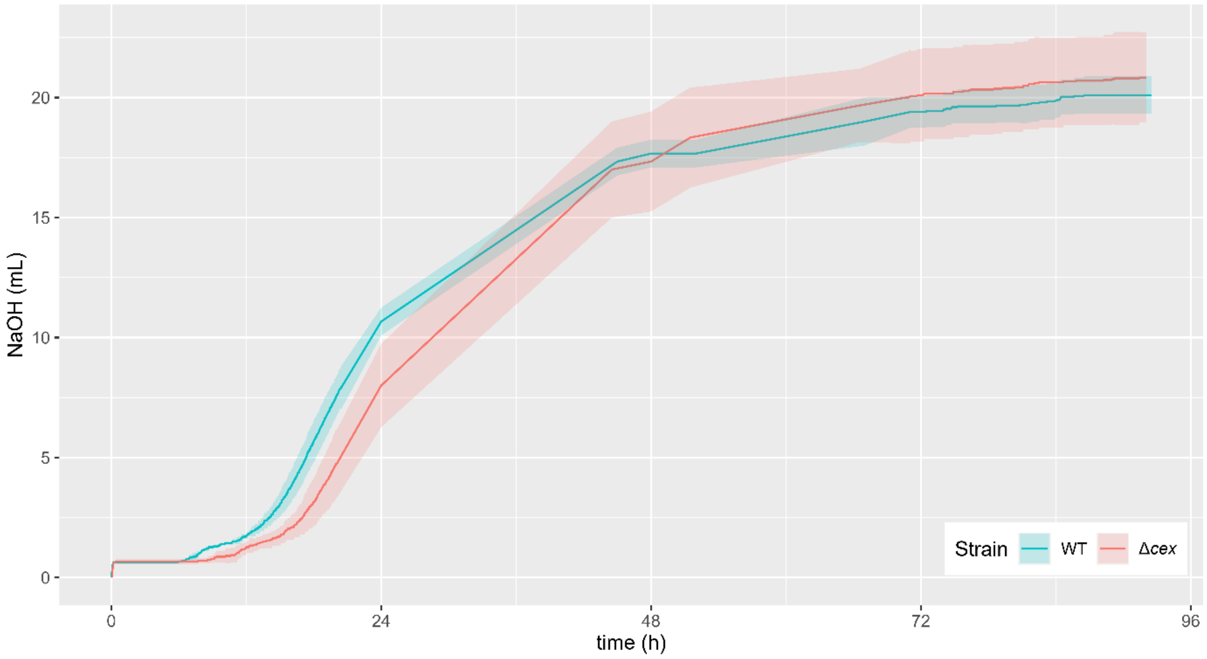

Figure S87. Growth curve of *C. thermocellum* DSM1313 wild type and Δ*cex* mutant strain in a 1L-fermenter on cellulose. Experiment done in triplicates.

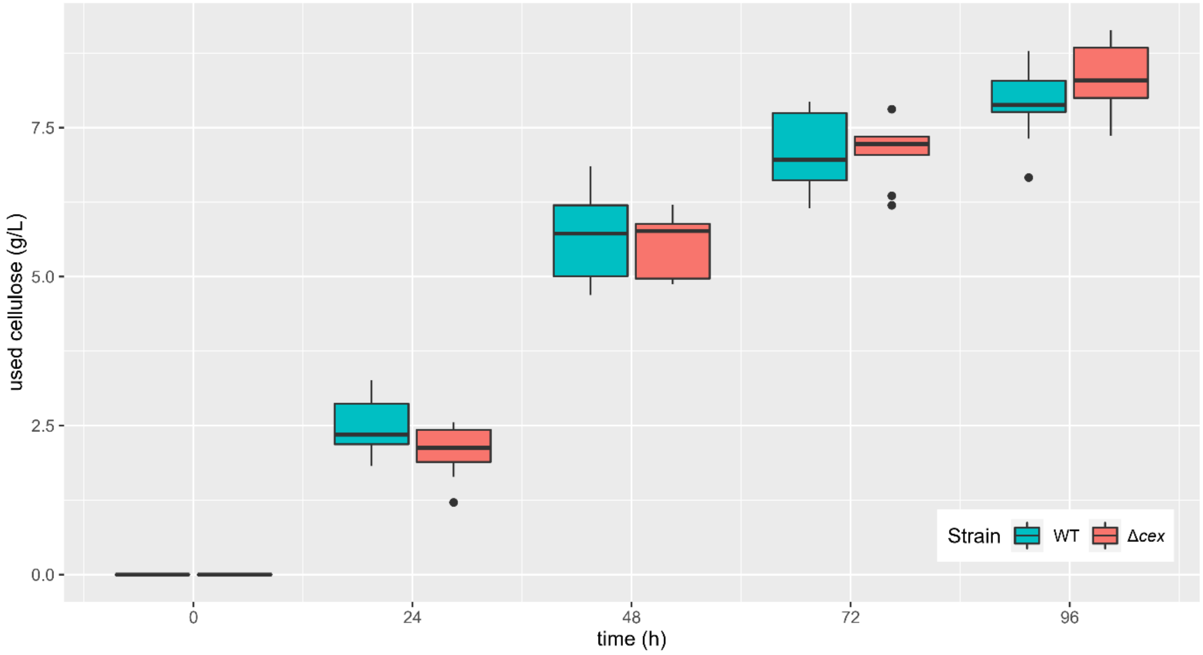

Figure S88. Cellulose consumption of *C. thermocellum* DSM1313 wild type and Δ*cex* mutant strain during growth in a 1L-fermenter on cellulose. From each fermenter three samples were taken at each time point resulting in nine values per strain and time point.

Supplemental References

[60] D. G. Olson, L. R. Lynd,"Transformation of *Clostridium thermocellum* by electroporation" *Methods Enzymol* **2012**, *510*, 317-330.

[61] E. A. Johnson, A. Madia, A. L. Demain,"Chemically defined minimal medium for growth of the anaerobic cellulolytic thermophile *Clostridium thermocellum*" *Appl environ microbiol* **1981**, *41*, 1060-1062.

[62] L. J. Klau, S. Podell, K. E. Creamer, A. M. Demko, H. W. Singh, E. E. Allen, B. S. Moore, N. Ziemert, A. C. Letzel, P. R. Jensen,"The Natural Product Domain Seeker version 2 (NaPDoS2) webtool relates ketosynthase phylogeny to biosynthetic function" *J Biol Chem* **2022**, *298*.

[63] L.-T. Nguyen, H. A. Schmidt, A. Von Haeseler, B. Q. Minh,"IQ-TREE: a fast and effective stochastic algorithm for estimating maximum-likelihood phylogenies" *Mol Biol Evol* **2015**, *32*, 268-274.

[64] S. Kalyaanamoorthy, B. Q. Minh, T. K. Wong, A. Von Haeseler, L. S. Jermiin,"ModelFinder: fast model selection for accurate phylogenetic estimates" *Nat methods* **2017**, *14*, 587-589.

[65] B. Q. Minh, M. A. T. Nguyen, A. Von Haeseler,"Ultrafast approximation for phylogenetic bootstrap" *Mol Biol Evol* **2013**, *30*, 1188-1195.

[66] S. Kumar, G. Stecher, K. Tamura,"MEGA7: molecular evolutionary genetics analysis version 7.0 for bigger datasets" *Mol Biol Evol* **2016**, *33*, 1870-1874.

[67] M. Herisse, K. Ishida, J. Staiger-Creed, L. Judd, S. J. Williams, B. P. Howden, T. P. Stinear, H.-M. Dahse, K. Voigt, C. Hertweck,"Discovery and biosynthesis of the cytotoxic polyene terpenomycin in human pathogenic Nocardia" *ACS Chemical Biology* **2023**, *18*, 1872-1879.

[68] R. Abdou, K. Scherlach, H.-M. Dahse, I. Sattler, C. Hertweck,"Botryorhodines A–D, antifungal and cytotoxic depsidones from *Botryosphaeria rhodina*, an endophyte of the medicinal plant Bidens pilosa" *Phytochemistry* **2010**, *71*, 110-116.

[69] S. Hon, D. G. Olson, E. K. Holwerda, A. A. Lanahan, S. J. Murphy, M. I. Maloney, T. Zheng, B. Papanek, A. M. Guss, L. R. Lynd,"The ethanol pathway from *Thermoanaerobacterium saccharolyticum* improves ethanol production in *Clostridium thermocellum*" *Metab Eng* **2017**, *42*, 175-184.
